# RNA-binding protein RCAN1.1L modulates *ATF2* mRNA stability to promote mitochondrial fission in acute ischemic stroke

**DOI:** 10.1038/s41419-026-08809-8

**Published:** 2026-05-13

**Authors:** Yanbin Ji, Xiaochun Ma, Liyuan Wang, Fangfei Liu, Yao Tang, Xiaxin Yang, Zexin Zhan, Shuangwu Liu, Juan Zhao, Tan Wang, Fuchen Liu, Dexin Yu, Yuguo Chen, Yan Yun, Xiulian Sun

**Affiliations:** 1 Department of Neurology, Qilu Hospital of Shandong University, Cheeloo College of Medicine, Shandong University, Jinan, China; 2 Department of Cardiovascular Surgery, Shandong Provincial Hospital Affiliated to Shandong First Medical University, Jinan, China; 3 Department of Otorhinolaryngology, NHC Key Laboratory of Otorhinolaryngology, Qilu Hospital of Shandong University, Jinan, China; 4 Department of Geriatric, Qilu Hospital of Shandong University, Jinan, China; 5 Department of Neurology, Research Institute of Neuromuscular and Neurodegenerative Diseases, Shandong Key Laboratory of Mitochondrial Medicine and Rare Diseases, Qilu Hospital of Shandong University, Jinan, China; 6 Department of Radiology, Qilu Hospital of Shandong University, Jinan, China; 7 Department of Emergency Medicine, Qilu Hospital of Shandong University, Jinan, China; 8 Shandong Provincial Clinical Research Center for Emergency and Critical Care Medicine, Institute of Emergency and Critical Care Medicine of Shandong University, Chest Pain Center, Qilu Hospital of Shandong University, Jinan, China; 9 Medical and Pharmaceutical Basic Research Innovation Center of Emergency and Critical Care Medicine, China’s Ministry of Education, Shandong Provincial Engineering Laboratory for Emergency and Critical Care Medicine, Key Laboratory of Emergency and Critical Care Medicine of Shandong Province, Key Laboratory of Cardiopulmonary-Cerebral Resuscitation Research of Shandong Province, Qilu Hospital of Shandong University, Jinan, China; 10 NMPA Key Laboratory for Clinical Research and Evaluation of Innovative Drug, Qilu Hospital of Shandong University, Jinan, China

**Keywords:** Stroke, Cell death in the nervous system, Cellular neuroscience

## Abstract

Regulator of calcineurin 1 (RCAN1) is an RNA-binding protein with diverse functions, the regulatory mechanisms underlying mitochondrial function in ischemic neuronal injury remain only partially understood. This study identified significantly elevated plasma RCAN1.1 levels in acute ischemic stroke (AIS) patients and demonstrated that mitochondrial translocation of RCAN1.1L ‌within the ischemic penumbra‌ aggravates cerebral infarction ‌by promoting pathological mitochondrial fission and neuronal apoptosis‌. Mechanistically, in AIS cell and mouse models, multi-omics screening identified *activating transcription factor 2* (*ATF2*) mRNA as a critical downstream target of RCAN1.1L. RCAN1.1L binds to the 2915-2935 nucleotide in the 3’-untranslated region (UTR) of *ATF2* mRNA, stabilizing its expression and promoting the accumulation of mitochondrial ATF2 (mtATF2) protein. MtATF2, in turn, binds to and upregulates mitochondrial fission 1 (FIS1) protein, thereby enhancing mitochondrial fission and driving intrinsic apoptosis. Notably, the RNA aptamer R1SR13 competitively binds to RCAN1.1L protein with *ATF2* mRNA, exerting neuroprotective effects by disrupting the RCAN1.1L-mtATF2-FIS1 axis. These findings identify RCAN1.1L as an upstream regulator of *ATF2* mRNA stability-mediated mitochondrial fission and apoptosis in ischemic penumbra neurons and highlight R1SR13 as a promising therapeutic candidate for preserving neuronal mitochondrial integrity.

## Introduction

Acute ischemic stroke (AIS) is the leading cause of disability and a major cause of mortality worldwide due to sudden cerebral blood flow cessation, leading to neuronal injury and death [1–3]. A central concept in AIS management is the ischemic penumbra region of brain tissue that, while metabolically viable, suffers from critical hypoperfusion, placing it at high risk of progressing to irreversible infarction without timely reperfusion [4, 5]. Despite advances in thrombolytic therapy and endovascular thrombectomy, only a limited number of patients benefit from timely reperfusion due to strict therapeutic windows, hemorrhagic risks and the scarcity of medical resources [6, 7]. Consequently, neuroprotective agents represent a crucial adjunctive strategy to preserve penumbral tissue and extend the window for effective intervention. A comprehensive understanding of the molecular mechanisms underlying AIS pathogenesis is therefore urgently needed to identify novel and clinically viable therapeutic targets [8, 9].

Regulator of calcineurin 1 (RCAN1), also known as Down syndrome critical region 1 (DSCR1), is located on chromosome 21q22 and consists of seven exons and six introns [10, 11]. Alternative mRNA splicing generates two principal transcript variants: *RCAN1.1 and RCAN1.4*. *RCAN1.1* generates two protein isoforms, RCAN1.1L (the long isoform) and RCAN1.1S (the shorter isoform), via alternative in-frame start codons [10]. Our previous studies demonstrated that RCAN1.1L is the predominant isoform in mouse brain and rat neurons and shares functional properties with RCAN1.1S [12, 13]. Multiple prior studies from our research group have confirmed that RCAN1 plays a critical role in the pathogenesis of Down syndrome and Alzheimer’s disease [10, 11, 14]. In the AIS mouse model, upregulation of RCAN1.1L is associated with reduced ATP generation and enhanced caspase-3 activation, indicating that RCAN1.1L represents a novel pathological mechanism of AIS [14–16]. However, the specific regulatory molecular mechanisms remain to be elucidated.

RNA-binding proteins (RBPs) are key post-transcriptional regulators of gene expression, often referred to as the “clothing” of mRNA [17, 18]. In eukaryotic cells, mRNA is transcribed, processed, and packaged into ribonucleoprotein complexes in the nucleus, then transported to specific cytoplasmic regions for spatially regulated translation and function [19]. As integral components of these pathways, RBPs play a central regulatory role throughout the entire process [20, 21]. Recently, our results indicate that RCAN1.1 is a novel RBP that binds to *adenine nucleotide translocator 1* (*ANT1*) mRNA and increases ANT1 protein, thereby causing mitochondrial dysfunction and inducing apoptosis [15, 22]. Comprehensive structural mapping localized the RNA-binding domain (RBD) of RCAN1.1L to amino acid residues 56-158. Through systematic evolution of ligands by exponential enrichment (SELEX) screening, we identified R1SR13 as a high-affinity RNA aptamer specifically interacting with this domain. In parallel, RNA immunoprecipitation followed by next-generation sequencing (RIP-seq) analysis revealed R1MR1-1 as a physiological RNA recognition motif for RCAN1.1L [12, 15]. Under normoxic conditions, R1SR13 inhibits RCAN1-mediated regulation of the NFAT and NF-κB signaling pathways, thereby providing neuroprotection. In both cellular and animal models of AIS, R1SR13 significantly reduces neuronal apoptosis induced by elevated RCAN1.1L expression [12, 15]. R1SR13, identified as a functional antagonist of RCAN1.1 through in vitro screening, likely exerts its effect by competitively binding RCAN1.1L and displacing endogenous mRNAs from their binding sites [23]. However, the specific endogenous mRNAs that naturally interact with RCAN1.1L and participate in this competition remain unclear.

Activating transcription factor 2 (ATF2), a basic leucine zipper protein within the activator protein-1(AP-1) family, plays a critical role in regulating cellular stress responses, hypoxia adaptation, and DNA damage repair [24, 25]. Notably, previous study has shown that phosphorylated ATF2 translocates to mitochondria, where it disrupts the HK1-VDAC1 complex, leading to mitochondrial dysfunction and apoptosis [26]. Under hypoxic conditions, ATF2 is activated and directly interacts with hypoxia inducible factor 1 alpha (HIF-1α), thereby promoting its transcriptional activity [27]. Additionally, ATF2 is also a mitochondrial protein capable of increasing mitochondrial permeability and promoting apoptosis under genotoxic stress [28]. However, the dynamics and functional role of mitochondrial ATF2 (mtATF2) in AIS remain uncharacterized. Therefore, this study aims to investigate the regulatory mechanism by which RCAN1.1L, as an RBP, modulates the translocation and downstream signaling of mtATF2.

Mitochondrial energy supply is crucial for neuronal function and survival, making mitochondria-based neuroprotection a promising strategy for AIS [29, 30]. Mitochondria maintain their morphology through dynamic processes of fission and fusion [31]. Disruption of this balance-particularly through excessive mitochondrial fission-has been strongly implicated in neuronal death during AIS [32]. Fission is now recognized as a precursor to apoptosis, and its inhibition has been shown to delay or prevent neuronal death [32]. Dynamin-related protein 1 (DRP1), a key regulator of fission, is recruited to the outer mitochondrial membrane (OMM) to mediate the fission process [33, 34]. Mitochondrial fission protein 1 (FIS1), an OMM protein, functions upstream of DRP1 in fission [35]. FIS1-mediated fission plays a crucial role in cell survival under stress conditions [36]. Therefore, we sought to determine whether mtATF2 regulates mitochondrial fission through modulation of FIS1.

In the present study, we demonstrate that RCAN1.1 expression is significantly upregulated in plasma samples from AIS patients, supporting its clinical relevance as a regulatory node in pathology. ‌In an AIS animal model, we demonstrated that RCAN1.1L upregulation in the ischemic penumbra promoted cerebral infarct enlargement and exacerbated neurological deficits.‌ Under ischemic conditions, RCAN1.1L binds and stabilizes *ATF2* mRNA, facilitating its translocation to the OMM in penumbra neurons. Subsequently, elevated mtATF2 binds to FIS1 and upregulates its expression, thereby promoting mitochondrial fission and neuronal apoptosis. Therefore, we have identified a novel regulatory mechanism in mitochondrial dynamics involving the RCAN1.1L-mtATF2-FIS1 axis in AIS. Importantly, by specifically interfering with RCAN1.1L-mt*ATF2* mRNA stabilizing activity, R1SR13 improves neurological outcomes in preclinical AIS models. Taken together, we identify a previously unrecognized mechanism whereby RCAN1.1L exacerbates ischemic damage through dysregulation of mitochondrial mRNA targeting. The discovery of the RCAN1.1L-*mtATF2* mRNA interaction as a druggable target and the neuroprotective candidate R1SR13 establishes a new therapeutic strategy for stroke intervention.

## Materials and methods

### Study approval

All human research procedures were approved by the Research Ethics Board of Qilu Hospital, Shandong University (Approval ID: KYLL-202306-074). The clinical trial was registered in the Chinese Clinical Trial Registry (no. ChiCTR2500096396). This study was carried out in full compliance with the principles of the Declaration of Helsinki. All animal procedures were approved by the Institutional Animal Care and Use Committee of Shandong University (Approval ID: DWLL-2021-064). All procedures involving animals were conducted in full compliance with the ARRIVE guidelines and the institutional and national standards for the care and use of laboratory animals.

### Participants

Patients with AIS were recruited from those admitted to Qilu Hospital of Shandong University between July 2023 and May 2024. Inclusion criteria were: (1) aged 18 years or older, and (2) diagnosed with AIS. Exclusion criteria included: (1) transient ischemic attack, (2) AIS onset time exceeding 2 weeks, (3) surgery within the past 3 months, (4) severe heart, lung, or kidney damage, (5) malignant tumors, (6) a history of bleeding disorders, and (7) pregnant or lactating women. Healthy controls (HCs) were required to be:(1) over 18 years old, and (2) without a history of cerebrovascular disease within the past 3 months. HCs were excluded if they had: (1) severe heart, lung, or kidney damage, (2) malignant tumors, (3) a history of bleeding disorders, (4) surgery within the past 3 months, or (5) pregnant or lactating women. Informed consent was obtained from all participants, either directly or through their legally authorized representatives, prior to their inclusion in the study.

Participants comprised 34 HCs and 77 AIS patients, who were further stratified by time from stroke onset into three groups: ≤6 h (*n* = 35), 6–24 h (*n* = 19), and 24 h to 2 weeks (*n* = 23). The severity of AIS in patients was assessed using the National Institutes of Health Stroke Scale (NIHSS). Demographic and clinical characteristics of the recruited AIS patients and HCs are detailed in Table S1.

### Magnetic resonance imaging (MRI) for human participants and mice

MRI data for this study were obtained using 3.0 T scanners, including the uMR 880 (United Imaging Healthcare, China), Prisma (Siemens Healthineers, Germany), and SIGNA Architect (GE Healthcare, USA), all equipped with a 20-channel head coil, at the Department of Radiology, Qilu Hospital of Shandong University. The standard protocol included FLAIR, DWI, T1WI, and T2WI sequences. Human imaging parameters were FLAIR (TR/TE = 8200/91 ms, voxel size=0.3 × 0.3 × 6.0 mm, 18 slices of 6 mm thickness) and DWI (TR/TE = 2790/50 ms, *b*-value = 1000 s/mm², voxel size=1.4×1.4×6.0 mm). For mouse studies, normal and AAV9-injected mice were anesthetized with 3% isoflurane, and MRI was performed on a 9.4 T scanner (BioSpec 94/20 USR, Bruker, Germany), using T2WI (TR/TE = 2500/33 ms, 45 slices of 0.3 mm thickness) and TOF angiography (TR/TE = 18/4.5 ms, slice thickness of 15 mm). Mice were maintained at 1% isoflurane during scanning, with body temperature controlled by a heating blanket and heart rate and respiration monitored via ECG. Brain infarct areas were measured 24 h after sham or MCAO surgery, and imaging data were processed to assess infarct size and vascular changes [37].

### Animals and stereotaxic microinjection adeno-associated virus

Male C57BL/6 mice (25–30 g, 6–8 weeks old) were purchased from Beijing Vital River Laboratory Animal Technology (Beijing, China). The mice were maintained in a controlled environment with a standardized 12-h light/dark cycle and provided with ad libitum access to food and water. Following random group allocation, all surgical procedures and subsequent outcome evaluations were conducted by investigators using a rigorously implemented double-blind protocol. The following adeno-associated viruses were constructed by Vigene Bio, Inc. (Shandong, China): AAV9-hSyn-*CON*-GFP (AAV-*CON*), AAV9-hSyn-*RCAN1.1L*-GFP (AAV-*RCAN1.1L*), AAV9-U6-*shCON*-GFP (AAV-*shCON*), AAV9-U6-*shRCAN1.1*-GFP (AAV-*shRCAN1.1*), AAV9-U6-*R-CON*-GFP (AAV-*R-CON*), and AAV9-U6-*R1SR13*-GFP (AAV-*R1SR13*).

Mice were anesthetized with isoflurane (4% for induction in a chamber and 1.5% for maintenance; R510-22-10, RWD Life Science, Shenzhen, China) prior to stereotaxic microinjection of 2 µL of adeno-associated virus (3 × 10^12^ viral genomes/mL) at a rate of 0.2 µL/min. Two injections were administered into the cortex at the following coordinates: AP: +0.3 mm, ML: −3 mm, DV: -2mm; Point 2: AP: −1.9 mm, ML: -3 mm, DV: −2 mm [12]. A heating blanket set to 37 °C was used to maintain the mice’s body temperature throughout the procedure. After 2 weeks, the injection efficiency was evaluated by measuring GFP fluorescence in brain sections.

### Permanent middle cerebral occlusion (pMCAO) animal model and cerebral blood flow (CBF) test

Mice were anesthetized using isoflurane, and the frontal and parietal scalp regions were shaved. CBF was monitored using Laser Speckle Contrast Imaging (LSCI; RFLSI III, RWD Life Science, Shenzhen, China) with a laser probe, following the manufacturer’s instructions to obtain baseline data. Subsequently, pMCAO was performed as previously described [38]. In brief, under isoflurane anesthesia, focal cerebral ischemia was induced in adult male C57BL/6 mice. A silicone-coated nylon monofilament with a heat-blunted tip was introduced into the external carotid artery (ECA), advanced retrograde to the carotid bifurcation, and then redirected anterograde into the internal carotid artery (ICA) until it occluded the origin of the middle cerebral artery (MCA), thereby inducing permanent focal ischemia. CBF measurements were repeated at 10 min and 24 h post-surgery using LSCI. Mice exhibiting a decrease in CBF of more than 70% compared to baseline were included in the study. Throughout the surgical procedure, mice were kept warm with a 37 °C heating blanket.

### RCAN1.1 measurements in human plasma and mice serum sample

Venous blood samples were obtained from the participants, with particular focus on AIS patients who had not undergone drug thrombolysis or mechanical thrombectomy. The blood samples were collected in anticoagulant tubes coated with ethylenediaminetetraacetic acid (EDTA). After centrifugation at 3000 × *g* for 10 min at 4 °C, human plasma was collected and stored at −80 °C for further analysis. For the mice, before removal of the brains, the hearts were punctured to collect blood for isolating serum. RCAN1.1 protein levels in both human plasma and mouse serum were measured using Enzyme-Linked Immunosorbent Assay (ELISA) kits specifically designed for RCAN1.1, which were obtained from Jiangsu Meimian Co., Ltd (China). Measurements were conducted following the manufacturer’s instructions.

### Assessment of neurological deficits and 2,3,5-Triphenyltetrazolium chloride (TTC) staining

Twenty-four hours after MCAO, neurological deficits were assessed using a 5-point scale, performed by a blinded observer based on established protocols [39]. The scale was as follows: 0, No detectable neurological deficit. 1, Flexion of the torso and contralateral forelimb when the animal is lifted by the tail. 2, Circling towards the contralateral side but normal posture at rest. 3, Reclination towards the contralateral side at rest. 4, Absence of spontaneous motor activity. The mice brains were rapidly frozen and sliced into 2 mm thick coronal sections. The sections were then immersed in a phosphate-buffered saline (PBS) solution containing 0.2% TTC (T8877-10G, Sigma, USA) for staining at 37 °C for 20 min. Following staining, images were captured with a digital camera, and cerebral infarct volume was calculated using ImageJ software.

### Rotarod test

Neuromotor coordination was assessed using a rotarod system (Panlab Harvard Apparatus) [40]. Prior to testing, animals were acclimated to the apparatus in a training session consisting of a single 200-s run at a constant speed of 10 rpm. For the formal test, the rotational speed was progressively increased from an initial 0 rpm to a maximum of 40 rpm over a period of 450 seconds. The primary outcome measures were the latency to fall (in seconds) and the rotational speed at the moment of falling (rpm), both recorded automatically. Only one test trial was conducted per mouse. Trials were excluded and repeated if the mouse completed passive rotations without attempting to walk.

### Corner test

The corner test was performed to assess lateralized turning behavior, as previously described [41]. Briefly, the apparatus consisted of two opaque wooden boards (30 × 20 cm each) joined at a 30° angle to form a corner, with a small opening at the vertex. A mouse was gently guided into the corner until the vibrissae on both sides of its face made simultaneous contact with the two boards, triggering a turning response. Each mouse performed 10 consecutive trials after a 5-min habituation period in the testing room. The direction (left or right) of each turn was recorded and the percentage of turns to each side was calculated.

### Nissl staining

Nissl staining was conducted using the Nissl staining assay kit (C0117, Beyotime) in accordance with the manufacturer’s instructions. Processed frozen sections were first immersed in 4% paraformaldehyde for at least 15 min, followed by two washes with distilled water to remove excess fixative. The sections were then stained with Nissl solution for 5 min, rinsed briefly with 95% ethanol for 5 seconds, and mounted with neutral resin. A layer of neutral resin was also applied to seal the mouse brain sections. For image acquisition and analysis, a panoramic scanning and image analysis system (Olympus VS120, Japan) was utilized to obtain high-definition images of the pathological sections.

### Cell culture, establishing of stable cell lines and siRNA assay

HEK293T and SH-SY5Y cells were cultured in Dulbecco’s Modified Eagle’s Medium (DMEM, high glucose, CM10013, Macgene Technology), supplemented with fetal bovine serum (04-001-1ACS, Biological Industries) and penicillin/streptomycin (P1400, Beijing Solarbio Science & Technology), as previously described [42]. Cells were maintained in a 37 °C incubator with 5% CO_2_. Lentiviral vectors used included Lentivirus-CMV-*CON*-GFP (Lv-*CON*), Lentivirus-CMV-*RCAN1.1L*-GFP-3flag (Lv-*RCAN1.1L*), Lentivirus-U6-*shCON*-GFP (Lv-*shCON*), Lentivirus-U6-*shRCAN1.1*-GFP (Lv-*shRCAN1.1*), Lentivirus-U6-*R-CON*-GFP (Lv-*shCON*), Lentivirus-U6-*R1SR13*-GFP (Lv-*R1SR13*), all of which contain the puromycin resistance gene and were purchased from Vigene Bio Inc. (Shandong, China). SH-SY5Y cells were infected with these lentiviruses at a multiplicity of infection of 1. The medium was replaced with fresh DMEM 24 h post-infection. To establish stable cell lines expressing RCAN1.1L protein (RCAN1.1L cell lines), knockdown RCAN1.1 protein (shRCAN1.1 cell lines), R1SR13 nucleic acid sequence (R1SR13 cell lines), and corresponding controls (CON, shCON, and R-CON cell lines), cells were continuously exposed to 2 μg/ml puromycin (ST551, Beyotime) for 2 weeks. Infection efficiency was assessed by GFP fluorescence and Western blotting analysis.

The ATF2 cDNA was amplified via PCR and inserted into the lentivirus vector pLentivirus-CMV-*ATF2*-RFP from Vigene Bio Inc. The lentiviral expression vector was co-transfected with psPAX2 and pCMV-VSV-G into HEK293T cells to produce lentivirus. After 48 h, lentivirus was harvested from the culture media. SH-SY5Y cells were then infected with Lentivirus-CMV-*ATF2*-RFP. Stable cell lines overexpressing *ATF2* mRNA and protein (ATF2 cell lines) were selected based on puromycin resistance. RFP fluorescence indicated successful infection, and *ATF2* mRNA and protein levels were significantly elevated in the ATF2 cell lines. Stable cell lines were maintained with 1 μg/ml puromycin.

To assess the efficiency of ATF2 knockdown, SH-SY5Y cells were transfected with ATF2 siRNA (*siATF2*: 5’-GGCGAGUCCAUUUGAGAAUTT-3’ and 5’-AUUCUCAAAUGGACUCGCCTT-3’) or a negative control (*siCON*) using Lipofectamine 3000 transfection reagent. After 48 h, cells were harvested, and *ATF2* mRNA and protein levels were detected using RT-PCR and Western blotting.

### Oxygen-glucose deprivation (OGD) treatment

SH-SY5Y cells and their stable derivatives were first removed from glucose-containing DMEM medium and washed twice with phosphate-buffered saline (PBS, pH 7.4, CC008, Macgene Technology). Following the washes, the cells were refreshed with glucose-free DMEM (11966025, Thermo Fisher Scientific). The experimental cells were then placed in an anaerobic chamber maintained at 37 °C, with a gas mixture of 95% N_2_ and 5% CO_2_ for the designated durations. Meanwhile, control cells were maintained in the original glucose-containing DMEM medium within a humidified atmosphere at 37 °C with 5% CO_2_.

### Transmission electron microscopy (TEM)

Ultrastructural analysis of neuronal mitochondria in mouse brain and cell samples was conducted by TEM following an optimized protocol [39]. In brief, fixation involved immersion in 3% glutaraldehyde plus 2% paraformaldehyde in 0.1 M cacodylate buffer (pH 7.4) for 2 h at room temperature, followed by post-fixation in 1% osmium tetroxide for 1 h. The samples were then dehydrated through an ascending ethanol series, embedded in resin, and sectioned into ultrathin slices using a diamond knife ultramicrotome. The sections were visualized and imaged using a Hitachi H-7800 TEM.

### Subcellular fractionation

Cellular proteins were isolated as cytoplasmic and nuclear fractions using the Minute™ Cytoplasmic and Nucleus Isolation Kit (SC-003, Invent Biotechnologies) [43]. Intact mitochondria were isolated from both mouse cerebral cortex and SH-SY5Y cells using the Mitochondria Isolation Kit for Mammalian Cells and Tissues (MP-007, Invent Biotechnologies) [44]. The proteins extracted from the isolated cell fractions were stored at -80°C until further analysis.

### Real-time quantitative PCR (RT-qPCR)

Total RNA was isolated from cells using Trizol reagent (15596018, Thermo Fisher Scientific) according to the manufacturer’s protocol. For the extraction of nuclear and cytoplasmic RNA, the Cytoplasmic & Nuclear RNA Purification Kit (NGB-21000, Norgen Biotek) was used, following the provided instructions. Mitochondrial RNA from SH-SY5Y cells was extracted following a previously established protocol [45]. RT-qPCR analysis was performed using the ABI 7900HT Fast Real-Time PCR System (Applied Biosystems, Foster City, CA, USA) and SYBR Green Realtime PCR Master Mix (QPK-201, Toyobo, Japan). Primer sequences used for qPCR amplification are listed in Table S2.

### Mitochondrial transcriptomics in mice brain

The extraction of mitochondrial mRNA from the mice cerebral cortex followed the previously established protocol [46]. RNA integrity was assessed using the RNA Nano 6000 Assay Kit on the Bioanalyzer 2100 system (Agilent Technologies, CA, USA). Messenger RNA was purified from total RNA using poly-T oligonucleotide-linked magnetic beads. Subsequently, cDNA synthesis was carried out using random hexamer primers after RNA fragmentation. Following end repair, A-tailing, adapter ligation, size selection, amplification, and cDNA purification, the transcriptomic library was prepared. Clustering of the index-coded samples was conducted on a cBot Cluster Generation System using the TruSeq PE Cluster Kit v3-cBot-HS (Illumina), adhering to the manufacturer’s instructions. After cluster generation, library preparations were sequenced on an Illumina NovaSeq platform, generating 150 bp paired-end reads.

Differential expression analysis between two groups, comprising three biological replicates per condition, was performed using the DESeq2 R package (version 1.20.0). A significance threshold was set at *p*-values < 0.05 and an absolute fold change of >0.5. Gene Ontology (GO) enrichment analysis of differentially expressed genes was conducted using the clusterProfiler R package, correcting for gene length bias. GO terms with corrected *p*-values < 0.05 were considered significantly enriched. Additionally, statistical enrichment of differentially expressed genes in KEGG pathways was assessed using the same clusterProfiler R package.

### Western blotting

Proteins were separated using sodium dodecyl sulfate-polyacrylamide gel electrophoresis (SDS-PAGE) and transferred to a Nitrocellulose membrane. The membrane was then incubated with specific primary and secondary antibodies, as outlined in Table S6. Blots were visualized using an Odyssey scanner (LI-COR, Lincoln, NE, USA). Densitometric analysis of the bands was conducted using Image J software to provide a quantitative assessment [13].

### Co-Immunoprecipitation coupled with mass spectrometry (Co-IP/MS)

To identify interacting proteins of ATF2, we performed Co-IP/MS. Mitochondrial proteins were precipitated using an anti-Flag antibody (M2, F1804, Sigma-Aldrich) from ATF2 cell lines treated with OGD for 0 and 6 h, following previously established protocols [47]. Liquid chromatography coupled with tandem mass spectrometry (LC-MS) detection was conducted using an L-3000 HPLC system (RIGOL, China) and an Orbitrap Eclipse mass spectrometer (Thermo Fisher Scientific). Database screening was performed against the Homo sapiens database using Proteome Discoverer 2.4 (Thermo Fisher Scientific). The LC-MS results indicated candidate genes that interacted with the ATF2 protein in the ATF2 cell lines after OGD 6 h, which were not present in the negative control, as detailed in Data S1.

For the validation of protein interactions, HEK 293 T cells were transfected with the plasmids pcDNA3.1-ATF2-3Flag and/or pcDNA3.1-Fis1-6Myc for 48 h. Additionally, SH-SY5Y cells were treated with OGD for either 0 or 6 h. Cells were lysed using western and immunoprecipitation (IP) lysis buffer (IP buffer, P0013J, Beyotime), and the supernatant was collected after centrifugation. The protein sample was incubated with the specific antibody at 4 °C overnight. Following this, Protein A/G Magnetic Beads (B23202, Selleck) were added to the mixture and incubated overnight at 4 °C. After incubation, the beads-bound immune complexes were washed three times with IP buffer, and the samples were subsequently analyzed using western blotting.

### Crosslinking immunoprecipitation followed by high-throughput sequencing (CLIP-seq) analysis

The CLIP assay was performed using a crosslinking-immunoprecipitation kit (Bes3014, BersinBio, China) according to the manufacturer’s instructions to identify specific RNA sequences that bind to the RCAN1.1L protein. Prior to protein-RNA cross-linking, 4-thiouridine (13957-31-8, TargetMol Chemicals Inc.) was added to the RCAN1.1L cell line culture medium at a final concentration of 100 μM to facilitate cross-linking. After 16 h, cells were washed with prechilled 1×PBS and exposed to 365 nm UV light at a dosage of 0.15 J/cm² for 10 minutes to induce cross-linking. The cells were then collected and lysed using cell lysis buffer with RNase T1 digestion.

The lysate was divided into two portions: one was used as the input sample, and the other was incubated with anti-Flag (M2, F1804, Sigma-Aldrich) or anti-IgG (A7028, Beyotime) antibody at 4 °C overnight. Protein-RNA immune complexes were captured using Protein A/G magnetic beads. The complexes were then eluted using DNase digestion and proteinase K (ST533, Beyotime). RNA was extracted from both the input sample and the protein-RNA complexes using Trizol reagent (15596018, Thermo Fisher Scientific) for subsequent RNA high-throughput sequencing analysis. RNA library preparation was performed using the NEB Next® Ultra™ RNA Library Prep Kit (New England Biolabs). Libraries were sequenced on the Illumina NovaSeq platform with a paired-end read length of 150 bp, following standard protocols. MACS2 (version 2.1.0) peak-calling software was utilized to identify regions of IP enrichment over background, with a *p*-value threshold of 0.05. Homer software (v4.9.1) was used to detect de novo sequence motifs and match known motifs. GO enrichment analysis was performed using the GO seq R package.

### RNA immunoprecipitation (RIP) assay

The RCAN1.1L cell lines were washed with prechilled 1×PBS and then lysed in approximately 300 μl of polysome lysis buffer (PLB) containing 0.5% Nonidet P-40 (NP40), 10 mM Hepes (pH 7.0), 0.1 M KCl, 5 mM MgCl_2_, 1 mM DTT, 200 units/ml recombinant RNase inhibitor (M0307L, New England Biolabs, Beijing, China), 0.2% vanadyl-ribonucleoside complex (R0107, Beyotime), 0.2 mM PMSF, and EDTA-free protease inhibitor. The lysate was horizontally mixed at 4 °C for 30 min and then centrifuged at 15,000 × *g* for 15 minutes to eliminate large particles. 30 µl of Protein A/G beads were mixed with 500 µl of NT2 Solution Buffer (NT2 Buffer, 50 mM Tris-HCl, 150 mM NaCl, 1 mM MgCl_2_, 0.05% NP40), and 2 µg of anti-Flag (M2, F1804, Sigma-Aldrich) or IgG (A7028, Beyotime) antibody at 4°C overnight. After centrifugation, the antibody-bound beads were resuspended in 850 µl of pre-chilled NT2 buffer containing 500 units of RNase inhibitor, 400 µM vanadyl ribonucleoside complex, 100 mM DTT, and 20 mM EDTA. The cell lysate was then added to this mixture and rotated at 4 °C for 4 h to allow for binding of Protein-RNA complexes. After incubation, the Protein-RNA complexes were eluted using proteinase K (ST533, Beyotime), and RNA was extracted using Trizol reagent (15596018, Thermo Fisher Scientific) for subsequent RT-PCR analysis.

### RNA pull-down

The biotin-labeled *ATF2* mRNA or biotin-labeled *ATF2* mRNA mutant probe, R1SR13 or R1SR13 mutant was obtained from GenePharma (Shanghai, China) in Table S5. The recombinant RCAN1.1L 56-158-6His protein (purified from Professor Yan Yun in our research team) or protein extract of RCAN1.1L cell lines expressing the RCAN1.1L-3flag fusion protein using pulldown buffer (25 mM Tris pH 7.4, 150 mM KCl, 50 mM NaCl, 5 mM EDTA, 0.5 mM DTT, 0.5% NP40, 10 μg/ml leupeptin, 10 μg/ml pepstatin, 10 μg/ml chymostatin, 1 mM PMSF, 100 U/ml RNase inhibitor) were mixed with biotin-labeled *ATF2* mRNA or biotin-labeled *ATF2* mRNA mutant (50 pmol) at 4 °C overnight. Additionally, the recombinant RCAN1.1L 56-158-6His protein was combined with varying concentrations of biotin-labeled *ATF2* mRNA and R1SR13 or its mutant RNA (1, 5, and 10 times more biotin-labeled RNA) under the same conditions. Following the incubation, BeyoMag™ Streptavidin Magnetic Beads (P2151, Beyotime) were added to each binding reaction and incubated at room temperature for 1 h. The beads were then washed five times with the pulldown buffer to remove unbound components. Proteins were eluted from the beads using 30 µl of 1×SDS protein lysis buffer at 95 °C and subsequently analyzed by western blotting.

### RNA and protein stability assay

The RCAN1.1L-, shRCAN1.1-, and negative control cell lines were treated with Actinomycin D (Act D; 1036-50, BioVision) at a final concentration of 5 μg/mL for 0, 2, 4, and 6 h. Following this treatment, the cells underwent OGD 6 h. Total RNA was extracted using the RNA Fast Extraction Kit (220010, Fastagen, Shanghai, China) according to the manufacturer’s instructions. The extracted RNA was then analyzed using RT-qPCR to evaluate the effects of the treatments. To determine the effect of ATF2 on FIS1 protein degradation, ATF2 and CON stable cell lines were treated with Cycloheximide (CHX; 100 μg/mL, 239763-M, Sigma-Aldrich), which inhibits protein synthesis by interfering with the translocation step. Cells were harvested at 0, 12, 24, and 36 h post treatment, and FIS1 protein levels were assessed by western blotting analysis.

### Trypsin proteolysis assay

A total of 50 μg of recombinant RCAN1.1L 56-158-6His protein was incubated with the in vitro transcribed *ATF2* mRNA (1–4164 nt), *ATF2* mRNA (2865–2963 nt), or *ATF2* mRNA (2964–3077 nt) at 25 °C. The mixture was then treated with trypsin (0.06 μg/μl, T1426, Sigma-Aldrich) for 30 min at 25 °C to digest the proteins. Following digestion, proteins were analyzed by western blotting to identify RCAN1.1L 56-158-6His and its corresponding fragments.

### In vitro RNA transcription

The *ATF2* mRNA 1-4164 bp and its fragments were amplified via PCR from the pUC57-ATF2 plasmid (Genscript. China) using SP6 primers. Refer to Table S3 for details on SP6 primers. The PCR products were separated via agarose gel electrophoresis, purified with Gel Extraction Kit (D2500, Omega Bio-Tek), and utilized as templates for in vitro transcription. RNA products were generated using a 50 μl transcription system containing 40 units SP6 RNA Polymerase (M0207S, New England Biolabs, Beijing, China), 2 μl RNA Polymerase Reaction Buffer, 0.5 mM of each ATP, UTP, GTP, and CTP (N0450S, New England Biolabs, Beijing, China), and 1 μg of DNA template with the SP6 phage promoter at 37 °C for 4 h. The DNA templates were removed with 2 units RNase-free-DNase I (EN0521, Thermo Fisher Scientific) at 37°C for 15 min. The transcribed RNAs were purified using phenol-chloroform extraction followed by ethanol precipitation.

### Surface plasmon resonance (SPR) assay

SPR experiments were conducted using a Biacore™ T200 system (GE Healthcare Europe, Freiburg, Germany) to confirm the interaction between the RCAN1.1L 56–158 protein and *ATF2* mRNA fragment. The *ATF2* RNA fragments were purchased from GenePharma (Shanghai, China). The recombinant RCAN1.1L 56–158 protein was immobilized onto a CM5 chip (29104988, GE Healthcare, Beijing, China) via amine coupling with sodium phosphate. Subsequently, the chip surface was blocked using Ethanolamine-HCl as the blocking agent. The analysis of RNA-protein binding was conducted in HBS-P buffer (8995084, GE Healthcare, Beijing, China). Following RNA injection, the chip surface was regenerated by flowing 3 mM NaOH. The K_D_ of the RNA-protein complexes was determined using Biacore T200 evaluation software.

### RNA electrophoretic mobility shift assay (RNA EMSA)

The RNA EMSA assay was performed as described previously [15]. The in vitro-transcribed *ATF2* mRNA fragment or a Cy3-labeled RNA (sequences provided in Table S4; GenePharma, Shanghai, China) was incubated with recombinant RCAN1.1L-56-158 protein in a 20 μl reaction containing RNA EMSA Binding Buffer. The incubation was performed at room temperature for 30 min. The RNA-protein complexes were separated using an 8% nondenaturing polyacrylamide gel. Subsequently, the gel was stained with 1×GeneGreen Nucleic Acid Dye (RT210, TIANGEN, China) and images were captured using an Tanon 5200 multifunctional gel imaging system (Biotanon, China).

### RNA scope in situ hybridization

The RNA scope in situ hybridization assay was performed using the RNA scope Multiplex Fluorescent Reagent Kit (323100, BioTechne) according to the manufacturer’s instructions. Frozen sections of the mouse cerebral cortex, subjected to either sham surgery or MCAO 24 h, were microinjected with AAV-*CON*, AAV-*RCAN1.1L*, AAV-*shCON*, AAV-*shRCAN1.1*, AAV-*R-CON*, AAV-*R1SR13* viruses. Following treatment with hydrogen peroxide, the sections were fixed and incubated overnight at 4 °C with an anti-TOM20 antibody (612278, BD Transduction Laboratories). Subsequently, the mouse *Atf2* mRNA probe was applied and hybridized at 40 °C for 2 h, with the Opal 570 dye (Red, Perkin Elmer, USA) used to label the probe. Detection of TOM20 protein was achieved using Alexa Fluor® 647 (Purple, ab150115, Abcam). Images were captured with a laser scanning confocal microscopy (LSM980) fluorescence microscope (Carl Zeiss, Jena, Germany) and analyzed using ZEN software.

### Seahorse bioenergetic flux analysis

Mitochondrial respiration was assessed with a Seahorse XFe24 analyzer. Stably transfected SH-SY5Y cells were plated in XF24 microplates (7000 cells/well). The cells underwent a mitochondrial stress test after being treated with either OGD 0 or 6 h. Mitochondrial stress tests were performed sequentially using 1 μM oligomycin, 1 μM Carbonyl cyanide p-(trifluoromethoxy) phenylhydroazone (FCCP), 0.5 μM rotenone, and 0.5 μM antimycin. After the assay, the data were normalized and analyzed using Seahorse wave software based on protein content in each well. Basal oxygen consumption rate, maximal oxygen consumption rate, ATP production, and nonoxidative respiration were measured.

### Mitochondrial Ca^2+^ measurement

Mitochondrial Ca²⁺ was quantified with the fluorescent indicator Rhod-2 AM (S1062S, Beyotime) following the manufacturer’s instructions. SH-SY5Y stable cell lines subjected to OGD 0 or 6 h were loaded with 1×Rhod-2 AM at 37 °C for 30 min, washed twice with PBS, and then visualized under an LSM980 fluorescence microscope (Carl Zeiss, Jena, Germany) and analyzed using ImageJ software.

### Measurement of intracellular reactive oxygen species (ROS) and mitochondrial membrane potential (Δψ_m_)

Intracellular ROS levels and mitochondrial membrane potential were assessed using the reactive oxygen species assay kit (DHE, C1300, Applygen Technologies Inc.) and the mitochondrial membrane potential assay kit with JC-1 (C2006, Beyotime), following the manufacturers’ instructions. The fluorescence intensity of dihydroethidium (DHE) was measured using flow cytometry (Beckman Coulter, USA), with 10,000 events collected per sample, and mean DHE fluorescence intensity analyzed using FlowJo software. For JC-1 staining, cells underwent similar OGD treatment and were incubated with 1 ml of the JC-1 staining working solution at 37 °C for 20 minutes. After two washes with JC-1 staining buffer (1X), images were captured using a fluorescence microscope (Olympus IX73, Japan).

### Dual luciferase assay

The HEK293T cells were co-transfected with pCMV-flag-RCAN1.1L-myc/his, pGL3-Jun2, and their respective negative control plasmids using Lipofectamine 2000. After OGD 6 h, the cells were harvested and then lysed with passive lysis buffer, and luciferase activity was measured using the Dual-Luciferase® Reporter Assay System (E1910, Promega, Madison, WI, USA), as previously described [13].

### Immunofluorescence

Immunofluorescence analysis was performed on treated SH-SY5Y cells and mouse brain slices, following previously established protocols [48]. To label mitochondria, the cells were incubated with 50 nM MitoTracker™ Deep Red (purple, A66440, Thermo Fisher Scientific) for 15 min at 37 °C in the dark. Subsequently, the cells were washed three times with pre-chilled PBS before proceeding with immunofluorescence staining. Analysis of protein fluorescence intensity on mitochondria was performed using ImageJ. The background was subtracted from all images. Mitochondrial regions of interest (ROIs) were then identified by thresholding the combined signals from TOM20 and MitoTracker. The mean fluorescence intensity of the target protein was calculated within these defined ROIs.

### Statistics

All in vitro experiments were performed with 3-4 biological replicates. Each experimental group in the in vivo mouse studies is presented as an individual data point. The sample sizes for the patient data are provided in their respective figures. Statistical analyses were conducted using SPSS 22.0 software (SPSS, Chicago, IL, USA) and Prism (GraphPad Software, Inc., San Diego, CA, USA). Data were evaluated for normal distribution and are presented as mean ± SEM. The correlations were statistically evaluated using Pearson correlation tests. Differences between two groups were assessed using a two-tailed, unpaired Student’s t test, the Mann-Whitney U test, or the chi-squared test, as appropriate. For comparisons among more than two groups, one-way or two-way ANOVA was performed, followed by post hoc Tukey’s test. The values of *P* < 0.05 were considered statistically significant.

## Results

### RCAN1.1L translocates to the OMM in AIS and is elevated in patient plasma

**Fig. 1:**
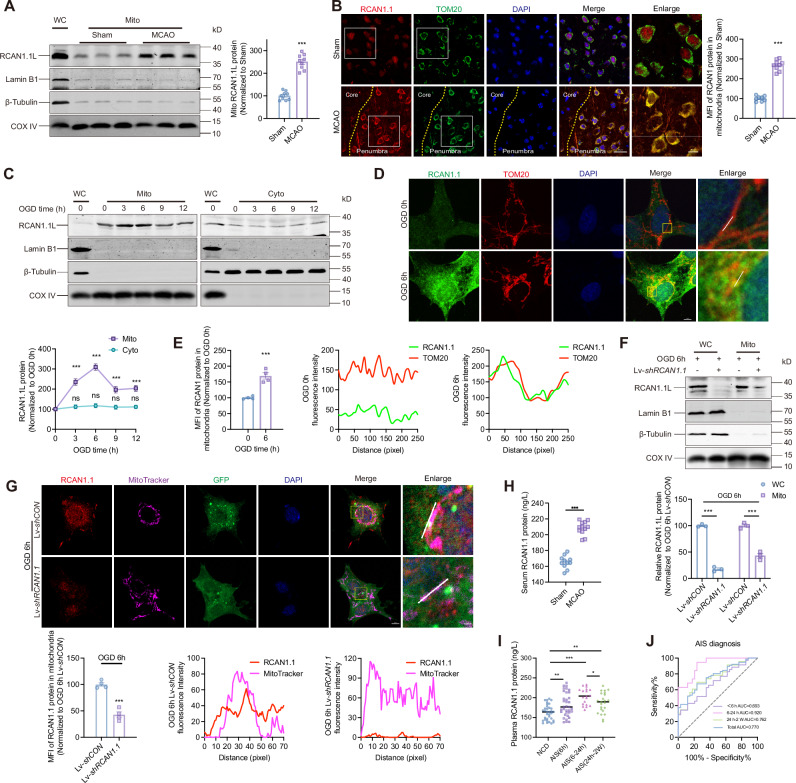
MtRCAN1.1L protein is upregulated in AIS animal and cellular models, and plasma RCAN1.1 shows potential as a diagnostic predictor. **A** Western blotting analysis of mtRCAN1.1L expression in the penumbra of mice subjected to sham surgery or MCAO for 24 h (*n* = 9). **B** Double immunofluorescence of RCAN1.1 (red) and TOM20 (green, OMM marker) in the penumbra of mice subjected to MCAO 24 h. Mean fluorescence intensity (MFI) of mitochondrial RCAN1.1 was analyzed using ImageJ. Images were captured using an LSM980 microscope (*n* = 8-9). Scale bars: 20 μm. **C** SH-SY5Y cells were subjected to OGD for varying durations (0-12 h). Whole-cell lysates (WC) or biochemically fractionated cytoplasmic (Cyto) and mitochondrial (Mito) fractions analyzed for RCAN1.1L protein levels using western blotting. Lamin B1, β-Tubulin, and COX IV served as loading controls for nuclear, cytoplasmic, and mitochondrial fractions, respectively (*n* = 3). **D**, **E** Immunofluorescence analysis of RCAN1.1 (green) and TOM20 (red) expression patterns in SH-SY5Y cells after OGD for 0 and 6 h (**D**). Images were captured using an LSM980 microscope. Fluorescence intensity of RCAN1.1 and TOM20 protein analyzed using line scan analysis in ImageJ (**E**) (*n* = 4). Scale bars: 5 μm. **F** SH-SY5Y cells with shRNA-mediated knockdown of RCAN1.1 (Lv-*shRCAN1.1*) and negative controls were subjected to OGD for 6 h. WC or Mito fractions were analyzed for RCAN1.1L protein levels using western blotting (*n* = 3). **G** SH-SY5Y cells with RCAN1.1 knockdown and negative controls subjected to OGD for 6 h. Cells were stained with MitoTracker (purple), fixed, permeabilized, and stained with DCT3 (RCAN1.1, red) and DAPI (blue) for nucleus. Images were captured using LSM980 microscope. Fluorescence intensity of RCAN1.1 and MitoTracker were analyzed using line scan analysis in ImageJ (*n* = 4). Scale bar: 5 μm. **H** Serum levels of RCAN1.1 protein were measured in MCAO mice using a mouse ELISA kit (*n* = 11-12). **I** Plasma levels of RCAN1.1 protein were assessed using ELISA across HCs, patients with AIS. AIS patients with AIS categorized by onset time intervals: <6 h (*n* = 35), 6-24 h (*n* = 19), and 24 h to 2 weeks (*n* = 23). **J** Receiver operating characteristic (ROC) curve analysis for RCAN1 protein in total AIS patients and those within different onset time intervals. Data presented as mean ± SEM. *P < 0.05, **P < 0.01, ***P < 0.001.

We confirmed widespread blockage of blood flow in the pMCAO model with MRI (Fig. S1A). Subsequent analysis revealed a significant increase in mitochondrial RCAN1.1L (mtRCAN1.1L) levels within the ischemic penumbra of the cerebral cortex in MCAO mice (Fig. 1A, B). In SH-SY5Y cells subjected to OGD, mtRCAN1.1L levels peaked at 310.1 ± 6.68% after 6 h and remained elevated for up to 12 h, while cytoplasmic RCAN1.1L levels were unchanged (Fig. 1C). We confirmed increased RCAN1.1 expression and its colocalization with TOM20 after OGD 6 h using LSM980. (Fig. 1D, E). SH-SY5Y cells were infected with lentivirus and subsequently selected with puromycin. Stable shRCAN1.1-knockdown SH-SY5Y cell lines were established by lentiviral transduction with Lv-*shRCAN1.1* and confirmed by RT-qPCR and western blotting (Fig. S1B, C). In *RCAN1.1*-knockdown SH-SY5Y cells, RCAN1.1L expression was significantly reduced in whole-cell lysates and mitochondrial fractions compared to controls (Fig. 1F, G). After OGD 6 h, RCAN1.1 colocalized with MitoTracker in control cells but not in knockdown cells (Fig. 1G). Thus, both in AIS mouse model ischemic penumbra neurons and in vitro cellular experiments, RCAN1.1 L was transported to the OMM.

To further validate the critical role of RCAN1.1 in the pathophysiological mechanisms of AIS, this study utilized ethically approved and clinically accessible specimens to measure and analyze RCAN1.1 expression levels in the plasma of AIS patients. First, RCAN1.1 expression levels were examined in the serum of MCAO mice. The results demonstrated that RCAN1.1 expression was significantly elevated compared to the sham group (Fig. 1H). Patients were diagnosed with AIS via cranial MRI (Fig. S1D), and detailed information on HCs and AIS patients is provided in Table S1. AIS patients were stratified into three groups based on onset time (Fig. S1E). Plasma RCAN1.1 levels were significantly elevated in AIS patients compared to HCs, with the most marked increase observed within 6–24 h post-onset (Fig. 1I). Receiver operating characteristic curve (ROC) analysis revealed an area under the curve (AUC) of 0.770 (95% CI: 0.68-0.86), suggesting that RCAN1.1 has substantial diagnostic potential (Fig. 1J). Further analysis of RCAN1.1 expression and its diagnostic performance across subgroups revealed that within the 6–24 h post-onset window, RCAN1.1 expression was at its peak, exhibiting excellent diagnostic accuracy with an AUC of 0.920, sensitivity of 94.74%, and specificity of 76.47% (Fig. 1I, J). Collectively, these findings demonstrate abnormally elevated RCAN1.1L expression in AIS and suggest its critical regulatory role.

### Elevated mtRCAN1.1L exacerbates brain injury induced by promoting neuronal mitochondrion-associated apoptosis

**Fig. 2:**
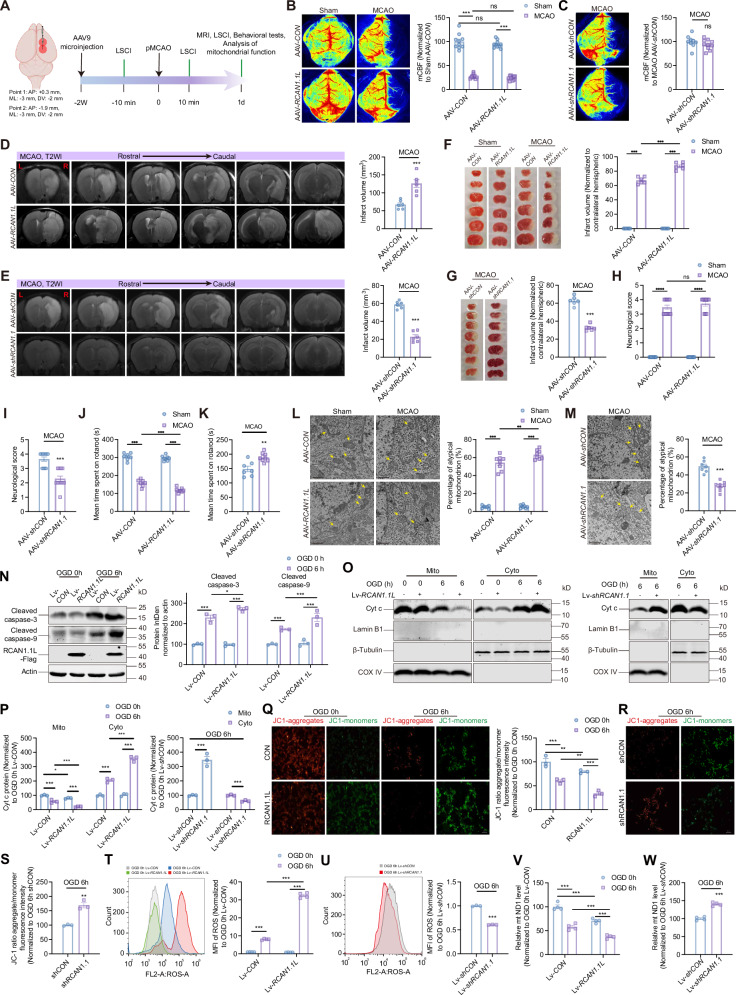
Upregulation of the RCAN1.1L protein level promotes neuronal mitochondrial damage and apoptosis in AIS models. **A** Schematic diagram outlining the mouse study, including stereotaxic microinjection of AAV9-hSyn-*CON*-GFP (AAV-*CON*), AAV9-hSyn-*RCAN1.1L*-GFP (AAV-*RCAN1.1L*), AAV9-U6-*shCON*-GFP (AAV-*shCON*), or AAV9-U6-*shRCAN1.1*-GFP (AAV-*shRCAN1.1*) viruses into the cerebral cortex. The two-site injection strategy is designed to target the cortical infarct area. Two weeks later, mice underwent LSCI assessments both before and after sham or MCAO surgery. Subsequently, MRI, LSCI, behavioral tests and mitochondrial function analyses were performed, respectively. Created in BioRender. ji, Y. (2026) https://BioRender.com/kameq99. Representative 2D LSCI of cerebral blood flow in AAV-*RCAN1.1L* (**B**), AAV-*shRCAN1.1* (**C**) and their control mice subjected to sham or MCAO for 24 h. Mean quantitative results were analyzed using LSCI software (*n* = 9–10). Representative T2WI data from AAV-*RCAN1.1L* (**D**), AAV-*shRCAN1.1* (**E**) and their control mice subjected to MCAO for 24 h (*n* = 6). Infarct volume was assessed using TTC staining in AAV-*RCAN1.1L* (**F)**, AAV-*shRCAN1.1* (**G**) and their control mice subjected to sham or MCAO for 24 h (*n* = 6). Neurological deficit scores for AAV-*RCAN1.1L* (**H**), AAV-*shRCAN1.1* (**I**) and their control mice after sham or MCAO for 24 h (*n* = 10-11). The mean time spent on rotarod was measured using the rotating rod test in AAV-*RCAN1.1L* (**J**), AAV-*shRCAN1.1* (**K**) and their control mice after sham or MCAO 24 h (*n* = 7–8). TEM was used to observe the ultrastructure of neuronal mitochondria in the penumbral region of AAV-*RCAN1.1L* (**L**), AAV-*shRCAN1.1* mice (**M**) and their control mice subjected to sham or MCAO for 24 h. Yellow arrows indicate neuronal mitochondria (*n* = 7-8). Scale bar: 1 μm. **N** RCAN1.1L and control SH-SY5Y cell lines were harvested after OGD 0 or 6 h to detect cleaved caspase-3, and cleaved caspase-9 proteins via Western blotting analysis (*n* = 3). **O**, **P** RCAN1.1L-, shRCAN1.1-, and their control cell lines were subjected to OGD 6 h and then biochemically fractionated into cytoplasmic (Cyto) and mitochondrial (Mito) fractions for analysis of Cyt c levels by Western blotting. Lamin B1, β-Tubulin, and COX IV served as loading controls for nuclear, cytoplasmic, and mitochondrial fractions, respectively (**O**). Protein levels were quantified using ImageJ (P) (*n* = 3). SH-SY5Y cells were transfected with pcDNA3.1-3flag (CON), pcDNA3.1-RCAN1.1L-3flag (**Q**, RCAN1.1L), pSUPER-shCON (shCON), and pSUPER-shRCAN1.1 (**R**, shRCAN1.1) plasmids. After OGD 0 or 6 h, JC-1 signal detected using fluorescence microscopy. Fluorescence intensity measured using ImageJ (**Q**, **S**) (*n* = 3). Scale bar: 20 μm. RCAN1.1L- (**T**), shRCAN1.1- (**U**), and control SH-SY5Y cell lines were treated with OGD 0 or 6 h, followed by measurement of ROS levels using flow cytometry (*n* = 3–4). Data are presented as mean ± SEM. *P < 0.05, **P < 0.01, ***P < 0.001. RCAN1.1L- (**V**), shRCAN1.1- (**W**), and their corresponding negative control SH-SY5Y cell lines were analyzed for mtND1 levels via RT-qPCR after OGD 0 or 6 h (*n* = 4). Data presented as mean ± SEM. *P < 0.05, **P < 0.01, ***P < 0.001.

The pathological role of elevated mtRCAN1.1L levels in the AIS mouse model was investigated by evaluating AIS outcomes and neuronal mitochondrial function (Fig. 2A). The cerebral cortex is a major site of ischemic injury in AIS, leading to motor, sensory, and cognitive deficits that underpin patient disability [49, 50]. Cortical infarction therefore critically determines functional prognosis, motivating our focus on the cortex [51, 52]. Leveraging its strong neurotropism, Adeno-associated virus 9 (AAV9) enables direct cortical delivery in mice for targeted RCAN1.1L modulation (AAV-*RCAN1.1L* and AAV-*shRCAN1.1*, respectively). The efficiency of AAV9 injection was confirmed 2 weeks post-injection by measuring GFP fluorescence in brain sections and verifying expression via western blotting (Fig. S2A, B). Following sham or MCAO surgery, regional CBF (rCBF) in the cortex was measured using LSCI (Figs. 2B, C and S2C). No significant differences in mean rCBF were observed between the AAV-*RCAN1.1L* and control groups post-MCAO (Fig. 2B). Similarly, there were no significant differences in the AAV-*shRCAN1.1* group compared to controls (Fig. 2C). Therefore, RCAN1.1L does not exert a detectable effect on blood flow as measured by LSCI.

For the AIS animal model, the outcomes include infarction volume and neurological function. 9.4 T MRI and TTC staining showed a significant increase in infarct volume in AAV-*RCAN1.1L* mice compared to controls (Fig. 2D–F and S2D), while AAV-*shRCAN1.1* mice exhibited a significant reduction in infarct areas 24 h post-MCAO (Fig. 2E–G). Neurological function scores in the AAV-*RCAN1.1L* group did not significantly differ from those of the control group, likely due to the high baseline scores (≥4 points) observed in the MCAO model controls (Fig. 2H). Conversely, AAV-*shRCAN1.1* mice exhibited significantly lower neurological function scores 24 h post-MCAO (Fig. 2I). Motor function, including the rotarod test and the corner test, was further assessed. In the rotarod test, the mean time spent on the rotarod was significantly shorter in the AAV-*RCAN1.1L* group than in the control group (Fig. 2J). Conversely, AAV-*shRCAN1.1* mice exhibited a significantly longer rotarod retention time after MCAO (Fig. 2K). In the corner test, no significant difference in left-turn percentage was observed between the AAV-*RCAN1.1L* group and the control group following MCAO (Fig. S2E). However, the left-turn percentage was significantly lower in AAV-*shRCAN1.1* mice after MCAO (Fig. S2F). Thus, RCAN1.1L upregulation worsens AIS mouse model outcomes. Subsequently, Nissl staining was employed to precisely delineate the ischemic penumbra.‌ In the AAV-*RCAN1.1L* group, the core infarct area expanded, and the penumbral zone shifted 24 h post-MCAO (Fig. S2G). By comparison, these effects were reversed in the AAV-*shRCAN1.1* group (Fig. S2H).

TEM analysis of mitochondrial morphology in the ischemic penumbra showed that, compared with the control group, AAV-*RCAN1.1L* mice exhibited a significantly higher percentage of atypical mitochondria following MCAO. These mitochondria displayed characteristic pathological features, including swelling, membrane rupture, and cristae fragmentation (Fig. 2L). In contrast, the AAV-*shRCAN1.1* group showed a marked reduction in the proportion of atypical mitochondria, along with improved ultrastructural morphology (Fig. 2M). Our prior studies demonstrated that RCAN1.1L promotes neuronal apoptosis in vitro [15]. Immunofluorescence analysis of the ischemic penumbra in the cerebral cortex of AAV-*RCAN1.1L* mice 24 h post-MCAO revealed significantly increased protein levels of cleaved caspase-3, and cleaved caspase-9 (Fig. S2I), indicating the activation of mitochondrial-mediated intrinsic apoptotic pathways in response to RCAN1.1L overexpression. A stable SH-SY5Y cell line overexpressing RCAN1.1L was established by lentiviral transduction of Lv-*RCAN1.1L*, and the results were verified by RT-PCR and Western blotting (Fig. S2J–L). Consistent results were observed in vitro, where Western blot analysis showed elevated levels of cleaved caspase-3 and cleaved caspase-9 in stable RCAN1.1L-overexpressing SH-SY5Y cells following OGD 6 h (Fig. 2N). However, in the shRCAN1.1 stable cell line, the levels of cleaved caspase-3 and cleaved caspase-9 were significantly decreased after OGD (Fig. S2M). Cytochrome c (Cyt c) serves as a pivotal mediator and indicator of mitochondrial-mediated apoptosis, activating the downstream caspase-9/3 apoptotic cascade [53–55]. In this study, Cyt c was released from mitochondria into the cytoplasm in RCAN1.1L-overexpressing cells after OGD 6 h; in contrast, RCAN1.1 knockout abolished this translocation (Fig. 2O, P).

The detrimental effects of RCAN1.1L on mitochondrial function were systematically investigated in cellular models, focusing on the following key parameters: (1) mitochondrial morphology; (2) mitochondrial energy metabolism, assessed through respiratory chain activity; (3) mitochondrial Ca²⁺ handling and Δψ_m_ measured by JC-1 staining; (4) ROS production; and (5) indicators of mitochondrial biogenesis, including PGC-1α expression and mitochondrial DNA copy number (mtDNA) [56–60]. TEM was employed to further examine the mitochondrial morphology in RCAN1.1L and shRCAN1.1 stably transfected SH-SY5Y cell lines following OGD 6 h. The results were consistent with those observed in animal models, thereby confirming the reliability of the research findings (Fig. S3A, B). To investigate the potential impact of RCAN1.1L on mitochondrial respiratory function, we measured the oxygen consumption rate (OCR) using an extracellular flux analyzer in stably transfected SH-SY5Y cells under OGD conditions. The results showed that basal respiration, ATP production, and maximal respiration were significantly reduced in RCAN1.1L-overexpressing cells, whereas these deficits were reversed in RCAN1.1-knockdown cells (Fig. S3C–E). Consistent with impaired respiratory function, western blot analysis revealed decreased protein levels of mitochondrial respiratory chain complexes I-IV in RCAN1.1L-overexpressing cells following OGD (Fig. S3F). In contrast, RCAN1.1 knockdown enhanced the expression of these complexes (Fig. S3G). Furthermore, we conducted fluorescence-based analysis to evaluate mitochondrial Ca²⁺ handling after OGD 6 h. Compared to the control group, mitochondrial Ca²⁺ levels were significantly elevated in RCAN1.1L-overexpressing cell lines following OGD, while RCAN1.1-knockdown cell lines showed a marked reduction in mitochondrial Ca²⁺ accumulation. (Fig. S3H, I). Then, overexpression of RCAN1.1L significantly impaired Δψ_m_ in SH-SY5Y cells exposed to OGD (Fig. 2Q), while RCAN1.1 knockdown effectively mitigated this decline (Fig. 2R, S).

Excessive ROS production was detected in SH-SY5Y cells following OGD 6 h (Fig. S3J). In the RCAN1.1L overexpression group, ROS levels were significantly elevated (Fig. 2T), while this increase was attenuated in RCAN1.1-knockdown cells (Fig. 2U). Western blot analysis of mitochondrial biogenesis markers after OGD 6 h revealed that, relative to control cells, PGC-1α protein levels were significantly reduced in RCAN1.1L-overexpressing cell lines; conversely, PGC-1α expression was elevated upon RCAN1.1 knockdown (Fig. S4A, B). MtDNA, assessed by measuring mtND1 levels, progressively decreased with prolonged OGD exposure, showing a significant reduction of 37.67 ± 6.47% at 12 h compared to baseline (Fig. S4C). In RCAN1.1L-overexpressing cells, mtND1 levels were significantly reduced (Fig. 2V), whereas shRCAN1.1 cells exhibited higher mtND1 levels after OGD (Fig. 2W).

Collectively, these findings indicate that mtRCAN1.1L upregulation exacerbates cerebral infarct volume and facilitates mitochondria-associated apoptosis by activating the Cyt c/caspase-mediated apoptotic cascade.‌

### RCAN1.1 L binds to *ATF2* mRNA and recruits it to OMM in AIS

**Fig. 3:**
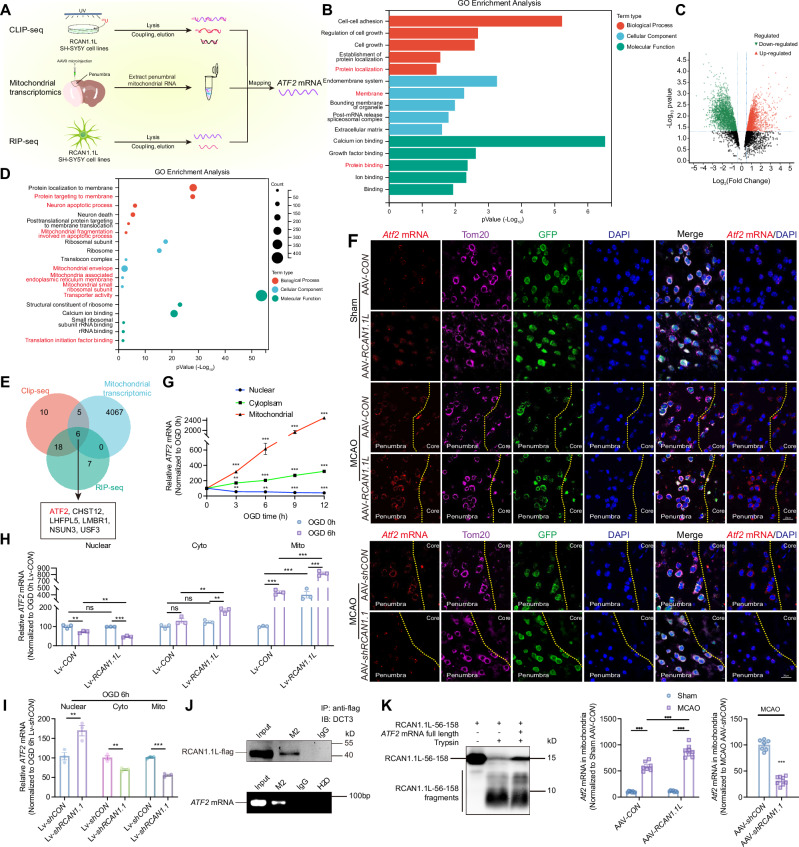
RCAN1.1L protein binds and accumulates *ATF2* mRNA on mitochondria under MCAO/OGD conditions. **A** Experimental design for identifying *ATF2* mRNA binding to RCAN1.1L protein based on CLIP-seq, mitochondrial transcriptomics, and RIP-seq. Created in BioRender. ji, Y. (2026) https://BioRender.com/hwxfxil. **B** Bar plots showing GO enrichment analysis of biological processes, cellular components, and molecular functions derived from CLIP-seq data. **C**, **D** Mitochondrial transcriptomics analysis of the penumbra of AAV-*RCAN1.1L* and control mouse cerebral cortex after MCAO for 24 h. Volcano plot of differentially expressed genes (**C**) and GO enrichment analysis of biological processes, cellular components, and molecular functions (**D**). **E** Venn diagram comparing the identified G-rich CM1 and R1MR1 motifs with the NCBI mRNA database and mitochondrial transcriptomics, revealing potential in vivo RCAN1.1L-binding mRNAs. **F** RNA scope in situ hybridization revealing the localization of *Atf2* mRNA to mitochondria after sham or MCAO in mice that received a microinjection of AAV-*CON*, AAV-*RCAN1.1L*, AAV-*shCON*, and AAV-*shRCAN1.1*. Mitochondrial *Atf2* mRNA fluorescence intensity was measured using ImageJ (*n* = 7-8). Scale bar: 20 μm. **G** SH-SY5Y cells were treated with OGD for indicated times (0-12 h). Cells were harvested and biochemically fractionated into nucleus, cytoplasm, and mitochondria. *ATF2* mRNA levels were detected using RT-qPCR. Homo sapiens S14, U2, and D-Loop were used as RT-PCR controls for cytoplasmic, nuclear, and mitochondrial fractions, respectively (*n* = 4). RCAN1.1L- (**H**), shRCAN1.1- (**I**), and negative control SH-SY5Y cells treated or not with OGD for 6 h. Cells were harvested and biochemically fractionated into nuclear, cytoplasmic, and mitochondrial fractions. *ATF2* mRNA levels were detected using RT-qPCR (*n* = 3). **J** RCAN1.1L cells were harvested, and the RCAN1.1L-RNA complex was pulled down using anti-Flag antibody for RIP. Western blotting (top) and RT-qPCR (bottom) were used to detected RCAN1.1 L protein and *ATF2* mRNA in the RNA-protein complex, respectively. **K** Recombinant protein RCAN1.1 L 56–58-6His incubated with in vitro-transcribed full-length *ATF2* mRNA, digested with trypsin. Western blotting was used to detect RCAN1.1 L 56–158 and its fragments (anti-His antibody). Data presented as mean ± SEM. *P < 0.05, **P < 0.01, ***P < 0.001.

To investigate whether RCAN1.1 L specifically regulates mitochondrial function through its RNA-binding capabilities, mitochondrial transcriptomic and CLIP-seq experiments were conducted in SH-SY5Y cells stably overexpressing RCAN1.1L (Fig. 3A and S5A). CLIP-seq results showed that GO analysis revealed its role in protein localization and association with membrane structures (Fig. 3B). Motif analysis identified a G-rich motif (CM1) with high affinity for RCAN1.1L (Fig. S5B).

Mitochondrial transcriptomic analysis in RCAN1.1L-overexpressing SH-SY5Y cells revealed significant dysregulation of 1726 upregulated and 2341 downregulated mRNAs (Fig. 3C). GO analysis highlighted processes related to protein targeting to membranes and neuronal apoptosis, with enrichment in the mitochondrial envelope, ribosomes, transporter activity, and translation initiation factor binding (Fig. 3D).

Comparing CM1 and R1MR1 motifs with the NCBI mRNA database, *ATF2* mRNA demonstrated high sequence alignment within its 3’-untranslated region (UTR) and was upregulated in the mitochondrial transcriptome (Fig. 3E). RNA scope in situ hybridization confirmed colocalization of mitochondrial *Atf2* mRNA with the OMM marker TOM20 under high RCAN1.1L expression after MCAO (Fig. 3F). In the MCAO model, elevated RCAN1.1L increased *Atf2* mRNA colocalization on mitochondria in the penumbral area, while RCAN1.1 knockdown reduced this colocalization (Fig. 3F).

In SH-SY5Y cells subjected to prolonged OGD, cytoplasmic *ATF2* mRNA levels increased, while nuclear *ATF2* mRNA levels decreased (Fig. 3G). Mitochondrial *ATF2* (*mtATF2*) mRNA levels also increased over time (Fig. 3G). After OGD 6 h, RCAN1.1L overexpression significantly increased cytoplasmic *ATF2* mRNA and decreased nuclear *ATF2* mRNA compared to controls (Fig. 3H). Conversely, shRCAN1.1 cell lines exhibited opposite trends (Fig. 3I). Mitochondrial extraction confirmed that RCAN1.1L overexpression increased *mtATF2* mRNA levels, while knockdown reduced these levels (Fig. 3H, I).

RIP experiments verified direct binding between RCAN1.1L protein and *ATF2* mRNA. RT-PCR detected *ATF2* mRNA in the co-precipitated complex (Fig. 3J). Trypsin digestion assays showed that full-length *ATF2* mRNA was protected from degradation after co-incubation with the purified RCAN1.1L-56-158, a truncated fragment of the RCAN1.1L protein containing the RBD (Fig. 3K). The data presented above indicate that RCAN1.1L, upon translocating to the OMM, binds to *ATF2* mRNA and modulates its expression level.

### RCAN1.1L modulates *ATF2* mRNA stability through direct binding to its 3’-UTR

**Fig. 4:**
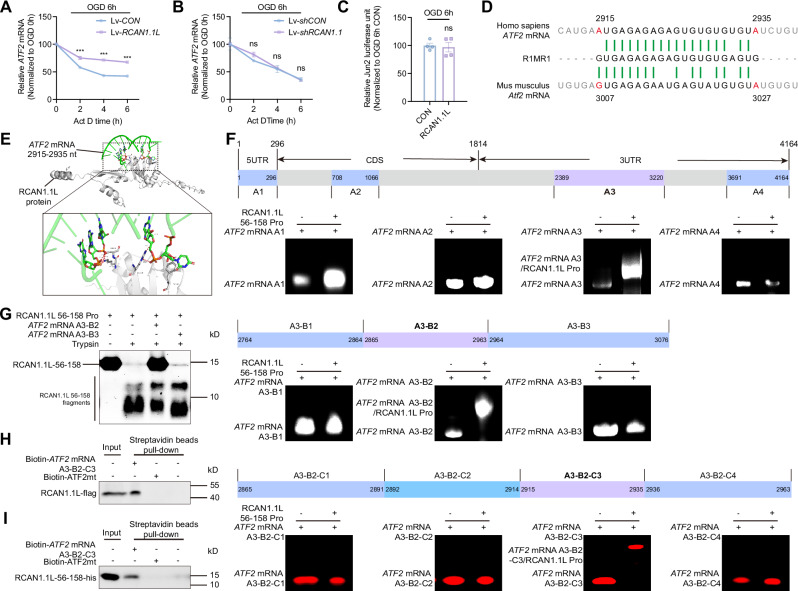
Verification of RCAN1.1L’s role in modulating *ATF2* mRNA stability and identification of RCAN1.1L binding sites within the 2915-2935 nt region of the *ATF2* mRNA 3’-UTR. RCAN1.1L- (**A**), shRCAN1.1- (**B**), and negative control SH-SY5Y cells were treated with actinomycin D for 0, 2, 4, and 6 h, followed by OGD for 6 h (*n* = 4). **C** HEK293 cells co-transfected with pGL3-basic-Jun2 Luc containing Jun2 promoter responsive elements or control plasmid and pcDNA3.1-RCAN1.1L or control plasmid. Cells were treated with OGD for 6 h, followed by a dual luciferase assay 48 h post transfection (*n* = 4). **D** Alignment of R1MR1 with Homo sapiens and Mus musculus *ATF2* mRNA transcripts using Clustal Omega. **E** Molecular docking overview of RCAN1.1L protein binding to *ATF2* mRNA 2915-2935 nt. The inset shows a magnification of the binding site; red dashed lines indicate hydrogen bonds. **F** Schematic diagram of *ATF2* mRNA, including the 5′-UTR, coding, 3′-UTR, and narrow regions. Blue and purple blocks represent in vitro-transcribed *ATF2* mRNA fragments. EMSA revealed protein-RNA migration bands at positions A3, A3-B2, and A3-B2-C3 of *ATF2* mRNA, not at other regions. **G** Recombinant protein RCAN1.1L-56-158-6His was incubated with in vitro-transcribed A3-B2 and A3-B3 regions of *ATF2* mRNA, followed by trypsin digestion. Western blotting was used to detect RCAN1.1L-56-158 and its fragments (anti-His antibody). RNA pulldown assay showed that RCAN1.1L-3FLAG protein (**H**) from extracts of RCAN1.1L SH-SY5Y cells or recombinant protein RCAN1.1L-56-158-6His (**I**) were pulled down by biotin-labeled RNA containing the RCAN1.1L-binding sequence in *ATF2* mRNA 2915–2935 nt (biotin-*ATF2* mRNA) and a random mutation sequence (biotin-ATF2mt, negative control). RCAN1.1L and RCAN1.1L-56-158 were detected using anti-Flag and anti-His antibodies in the RNA-protein complex pulled down with streptavidin beads. Data presented as mean ± SEM. *P < 0.05, **P < 0.01, ***P < 0.001.

Under Act D exposure and varying OGD durations, *ATF2* mRNA degradation was significantly delayed in the high RCAN1.1L expression group, starting from OGD 2 h (Fig. 4A). Conversely, *ATF2* mRNA degradation rates did not substantially differ between shRCAN1.1 and shCON cell lines (Fig. 4B). However, after OGD 6 h, RCAN1.1L overexpression did not enhance JUN2 luciferase activity (Fig. 4C), indicating that RCAN1.1L does not regulate *ATF2* transcriptional activity through transcriptional modulation.

Clustal Omega was used to align the R1MR1 sequence, and potential binding sites were identified at positions 2915-2935 nucleotide (nt) region within the 3’-UTR of Homo sapiens *ATF2* mRNA and 3007-3027 nt in Mus musculus *Atf2* mRNA (the first nucleotide of each full-length mRNA was designated as +1; Fig. 4D). AlphaFold 3 generated protein and RNA structures for molecular docking simulations [61]. The highest-scoring complex was selected for analysis. PyMOL revealed that the RBD of RCAN1.1L spans amino acids 56-158, including lysine 104, serine 105, arginine 108, and tyrosine 139 (Fig. 4E), which form hydrogen bonds with *ATF2* mRNA at 2915–2935 nt.

To verify the aforementioned prediction, RNA EMSA experiments were carried out. Truncated fragments of *ATF2* mRNA were transcribed from regions 1-297 nt (A1), 708-1067 nt (A2), 2389-3221 nt (A3), and 3693-4164 nt (A4) using SP6 RNA polymerase (Fig. 4F). RNA EMSA results showed a shifted RNA-protein band upon co-incubation of purified RCAN1.1L 56-158 fragment with A3, indicating a binding site within A3. Further refinement identified binding within the 2865-2963 nt region (A3-B2). Only A3-B2 exhibited binding to RCAN1.1L 56-158 (Fig. 4F). Incubation of A3-B2 with RCAN1.1L 56-158 protected it from trypsin degradation (Fig. 4G). Four smaller fragments, A3-B2-C1 to A3-B2-C4, labeled with CY3 fluorescent groups, were incubated with RCAN1.1L 56-158. Only A3-B2-C3 exhibited binding affinity (Fig. 4F). SPR confirmed *K*_D_ values of 7.231 × 10^−3^, 2.754 × 10^−3^, 6.892 × 10^−6^, and 3.275 × 10^−2^ M, respectively, highlighting the high affinity of A3-B2-C3 for RCAN1.1L 56-158 (Table 1).

**Table 1:** The analysis of the interaction between recombinant protein RCAN1.1L 56-158 and *ATF2* mRNA using SPR.

Name	Nucleotide sequence	Location	Binding affinity (*K*_D_, M)
*ATF2* mRNA A3-B2-C1	CAGUAUCUAUCUUUAGAACAAUGUAAU	2865–2891 nt	7.231 × 10^−3^
*ATF2* mRNA A3-B2-C2	UAUAAUGUGGGAAGUGUGCAUGA	2892–2914 nt	2.734 × 10^−3^
*ATF2* mRNA A3-B2-C3	AUGAGAGAGAGUGUGUGUGUA	2915–2935 nt	6.831 × 10^−6^
*ATF2* mRNA A3-B2-C4	UCUGUGUGUGUGUGCGCGUGUGUGUGUG	2936–2963 nt	3.274 × 10^−2^

An RNA pull-down assay using biotin-labeled A3-B2-C3 successfully pulled down RCAN1.1L and RCAN1.1L 56-158 from total protein extracts of RCAN1.1L-overexpressing SH-SY5Y cells and recombinant RCAN1.1L 56-158-6His. Mutant *ATF2* mRNA (ATF2mt) did not bind RCAN1.1L or RCAN1.1L 56–158 (Fig. 4H, I). Collectively, these findings establish *ATF2* mRNA 2915–2935 nt as the specific binding site for RCAN1.1L protein.

### Upregulated mtRCAN1.1L enhances mtATF2 protein under AIS conditions

**Fig. 5:**
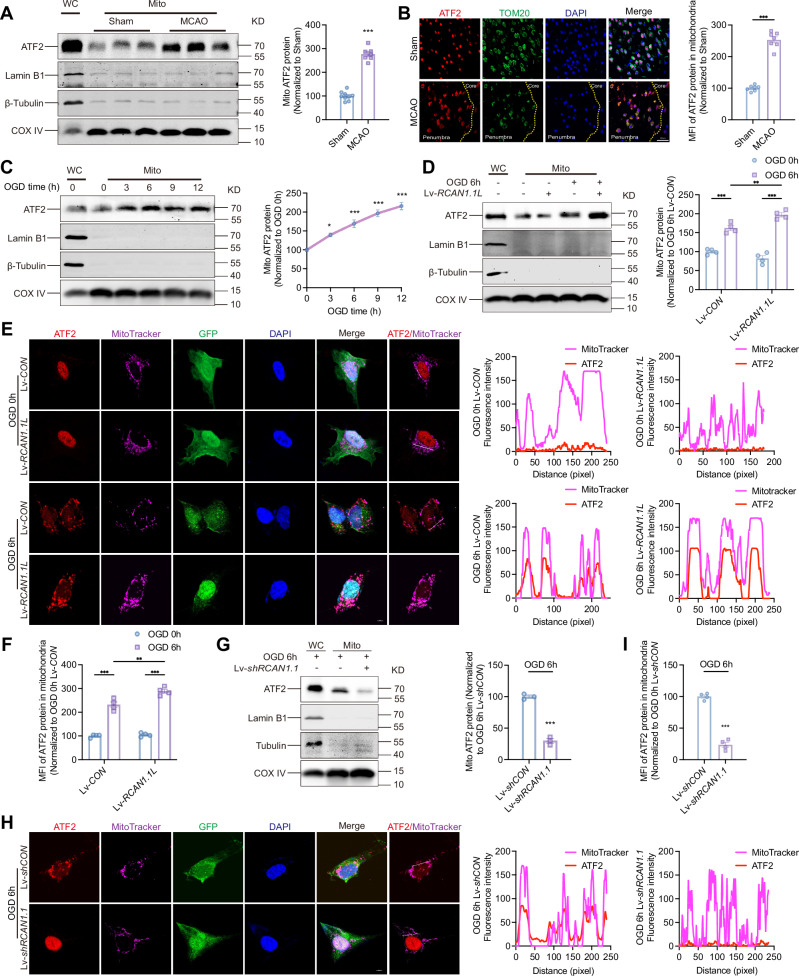
Upregulation of mtRCAN1.1L modulates mtATF2 protein under AIS conditions. **A** Western blotting analysis of mtATF2 protein expression in the penumbra of mice subjected to MCAO for 24 h (*n* = 7-9). **B** Immunofluorescence of ATF2 protein in the penumbra of mice subjected to MCAO for 24 h. Double immunofluorescence staining of ATF2 (red) and TOM20 (mitochondrial marker, green), with nuclei stained with DAPI (blue). Images were captured using an LSM980 microscope. Mitochondrial ATF2 fluorescence intensity was measured using ImageJ (*n* = 7). Scale bars: 20 μm. **C** SH-SY5Y cells were treated with OGD for indicated times (0–12 h), then harvested for WC and Mito fractions. ATF2 protein levels were detected using Western blotting. Lamin B1, β-tubulin, and COX IV served as loading controls for cytoplasmic, nuclear, and mitochondrial fractions, respectively (*n* = 3). RCAN1.1L- (**D**), shRCAN1.1- (**G**), and negative control cell lines were treated with OGD for 0 or 6 h. Cells were harvested for WC and Mito fractions. The ATF2 proteins levels were detected using Western blotting (*n* = 3–4). **E**, **F**, **H**, **I** RCAN1.1L- (**E**), shRCAN1.1- (**H**), and negative control SH-SY5Y cells were subjected to OGD for 0 or 6 h. Colocalization between MitoTracker and ATF2 was examined and intensity profiles of the indicated proteins were analyzed using line scan analysis in ImageJ. Mitochondrial ATF2 fluorescence intensity was also measured using ImageJ (**F**, **I**). Images were captured using an LSM980 microscope. Scale bar: 5 μm. Data presented as mean ± SEM. *P < 0.05, **P < 0.01, ***P < 0.001.

Western blotting and immunofluorescence revealed significant increases in ATF2 protein levels within the mitochondrial fraction of the penumbra in MCAO mice (Fig. 5A, B). MtATF2 protein levels progressively increased in SH-SY5Y cells subjected to OGD, with significant increases at 3, 6, 9, and 12 h post-OGD (Fig. 5C).

In SH-SY5Y cells overexpressing RCAN1.1L, mtATF2 levels markedly increased after OGD 6 h (Fig. 5D–F). Conversely, RCAN1.1 knockdown significantly decreased mtATF2 levels (Fig. 5G–I). Therefore, after RCAN1.1L binds to and upregulates *mtATF2* mRNA, it enhances ATF2 protein expression. These findings collectively suggest that upregulated mtRCAN1.1 L interacts with *ATF2* mRNA, facilitating its accumulation in mitochondria and subsequently leading to an increase in mtATF2 protein levels.

### In AIS, upregulated mtATF2 binds to and enhances FIS1 protein, activating mitochondrial fission

**Fig. 6:**
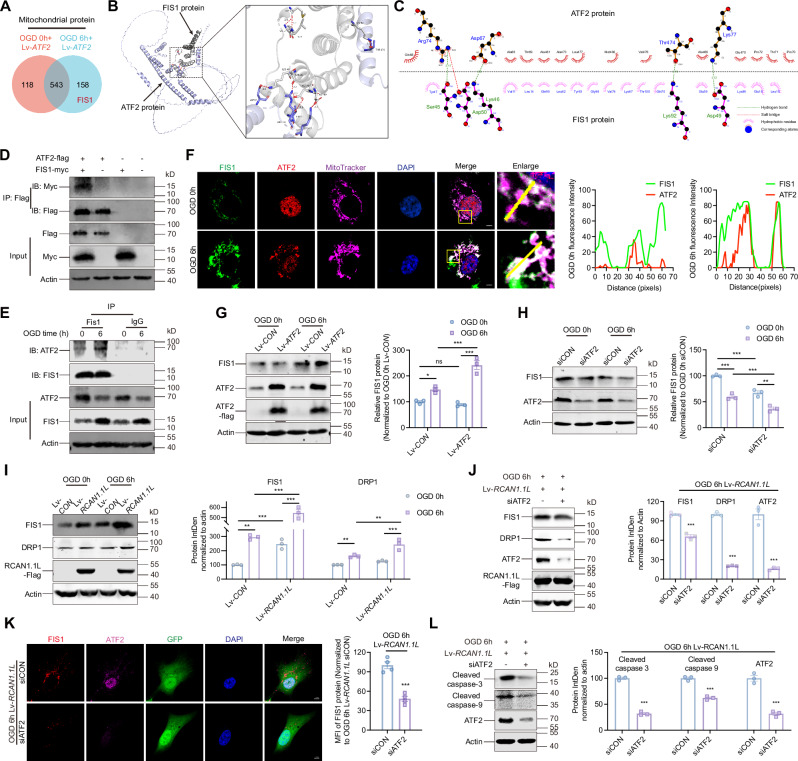
RCAN1.1L protein promotes mitochondrial fission and apoptosis by the interaction between mtATF2 and FIS1 protein. **A** Venn diagram of the number of ATF2-interacting candidate proteins identified by LC-MS analyses in ATF2 SH-SY5Y cells subjected to OGD for 0 or 6 h (*n* = 3). Molecular docking showed specific binding of ATF2 and FIS1. 3D diagrams illustrating ATF2 binding to the FIS1 channel (**B**) and 2D diagrams demonstrating interactions between ATF2 and FIS1 via hydrogen bonds and salt bridges (**C**). **D** Co-IP analysis of the exogenous interaction of ATF2 with FIS1 in HEK293T cells transfected with plasmids expressing ATF2-3Flag and FIS1-6Myc. **E** Co-IP analysis of the endogenous interaction of ATF2 with FIS1 protein in SY5Y cells treated with OGD 0 or 6 h. **F** Representative LSM980 images showed ATF2 (red), FIS1 (green), and MitoTracker (purple) in SH-SY5Y cells subjected to OGD for 0 or 6 h. Colocalization between ATF2 and FIS1 was examined and intensity profiles of the indicated proteins were analyzed using line scan analysis in ImageJ. Scale bars: 5 μm. **G** ATF2 and negative control SH-SY5Y cell lines were harvested after OGD for 0 or 6 h to detect FIS1 protein levels using Western blotting (*n* = 3). **H** FIS1 protein levels were detected in SH-SY5Y cells treated with siCON or siATF2 after OGD for 0 or 6 h using Western blotting (*n* = 3).**I** RCAN1.1L and negative control SH-SY5Y cells were harvested after OGD for 0 or 6 h to detect FIS1, DRP1, and RCAN1.1L proteins using Western blotting. Quantification was conducted using ImageJ (*n* = 3). **J** Western blot analysis of FIS1 and DRP1 protein levels in RCAN1.1L cells treated with siATF2 or siCON following OGD 6 h, and quantified using ImageJ (*n* = 3). **K** Representative images of ATF2 (red) and FIS1 (green) protein in the RCAN1.1L cells treated with siATF2 or siCON after OGD for 6 h. Scale bar: 5 μm. **L** Cleaved caspase-3 and cleaved caspase-9 expression was determined by western blotting in RCAN1.1L cell lines following siRNA-mediated knockdown of ATF2 (vs. siCON) and exposure to OGD 6 h. (*n* = 3). Data presented as mean ± SEM. *P < 0.05, **P < 0.01, ***P < 0.001.

SH-SY5Y cells were infected with lentivirus-CMV-ATF2-RFP (Fig. S6A–C) to overexpress mtATF2 (Fig. S6D). Under hypoxic conditions, LC-MS identified 158 proteins specifically interacting with mtATF2; no such interactions were observed under normoxic conditions (Fig. 6A and Data S1). The comparative analysis indicates that FIS1, located in the OMM, is a highly promising candidate.

UniProt sequences of human ATF2 and FIS1 were modeled and subjected to molecular docking using AlphaFold 3. The highest-scoring complexes were visualized through 3D simulations (Fig. 6B). Ligplot analysis revealed that arginine 74, aspartic acid 67, lysine 77, and threonine 474 within ATF2 form hydrogen bonds with FIS1. Additionally, arginine 74 in ATF2 forms a salt bridge with aspartic acid 50 in FIS1, along with numerous hydrophobic interactions (Fig. 6C). Co-IP assays confirmed direct interaction between ATF2 and FIS1 in vitro (Fig. 6D).

To explore endogenous interactions under hypoxic conditions, SH-SY5Y cells were analyzed after OGD 6 h. Significant binding between ATF2 and FIS1 was observed only after OGD 6 h (Fig. 6E). Immunofluorescence confirmed colocalization of mtATF2 and FIS1 after OGD 6 h (Fig. 6F). Overexpression or knockdown of ATF2 in SH-SY5Y cells resulted in increased (Fig. 6G) or decreased (Fig. 6H) FIS1 levels, respectively. As ATF2 and FIS1 exhibit a positive correlation in expression following co-localization, we further examined the degradation kinetics of the proteins to elucidate the underlying regulatory mechanism. The stability of the FIS1 protein was assessed using a CHX chase assay. The results showed that FIS1 protein stability was significantly increased in ATF2-overexpressing cells compared to controls. Thus, following co-localization, upregulation of ATF2 slows down the degradation rate of the FIS1 protein (Fig. S6E).

FIS1 serves as a marker protein for mitochondrial fission, and pathologically enhanced mitochondrial fission acts as a precursor to mitochondrial-mediated apoptosis [62]. DRP1 is another protein marker involved in mitochondrial fission. Significant increases in FIS1 and DRP1 were observed in RCAN1.1L-overexpressing SH-SY5Y cells after OGD 6 h, confirming RCAN1.1L-induced mitochondrial fission (Fig. 6I).

ATF2-specific siRNA effectively reduced *ATF2* mRNA and protein levels (Fig. S6F, G). Knockdown of ATF2 in RCAN1.1L-overexpressing cells decreased FIS1 and DRP1 levels, indicating suppressed mitochondrial fission after OGD 6 h (Fig. 6J, K). Mitochondrial fission and fusion jointly maintain mitochondrial homeostasis [62]. To investigate whether RCAN1.1L affects these additional mitochondrial processes, we examined key marker proteins associated with mitochondrial fusion, including OPA1, MFN1. Western blot analysis revealed that overexpression of RCAN1.1L in SH-SY5Y cells did not significantly alter the levels of these proteins (Fig. S6H). While ATF2 knockdown increased mitochondrial biogenesis in RCAN1.1L stable cell lines compared to the control after OGD, it did not alter mitochondrial fusion or autophagy (Fig. S6J, K). Additionally, ATF2 knockdown reduced cleaved caspase-3, and cleaved caspase-9 levels, suggesting attenuated intrinsic apoptosis in RCAN1.1L-overexpressing SH-SY5Y cells after OGD (Fig. 6L). Thus, under ischemic stress, mtATF2 is upregulated and co-localizes with FIS1 as a result of RCAN1.1L translocation to the OMM. These findings confirm that RCAN1.1L influences FIS1 protein via ATF2 on mitochondria, thereby promoting mitochondrial fission and inducing apoptosis.

### The RCAN1.1L-binding aptamer R1SR13 protects mitochondria by inhibiting the binding of *ATF2* mRNA

**Fig. 7:**
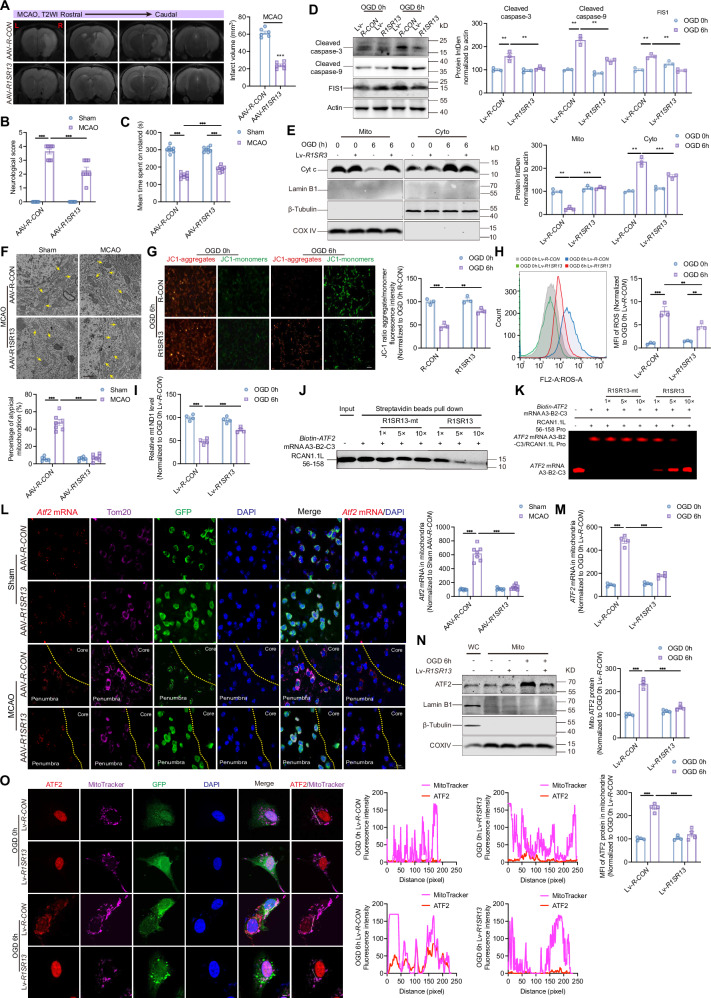
R1SR13 mitigates brain injury by competing with *ATF2* mRNA for binding to RCAN1.1 L protein in AIS. **A** Representative T2WI data from AAV-*R-CON* and AAV-*R1SR13* mice subjected to MCAO for 24 h (*n* = 6). **B** Neurological deficit scores for AAV-*R-CON* and AAV-*R1SR13* mice after sham or MCAO 24 h (*n* = 7–8). **C** The mean time spent on rotarod was measured using the rotating rod test in AAV-*R-CON* and AAV-*R1SR13* mice after sham or MCAO 24 h (*n* = 7–8). **D** Western blot analysis of Cleaved caspase-3, Cleaved caspase-9, and FIS1 in R1SR13 and negative control SH-SY5Y cell lines after OGD 0 or 6 h (*n* = 3). **E** R1SR13 and their control cell lines were subjected to OGD 0 or 6 h and then biochemically fractionated into cytoplasmic (Cyto) and mitochondrial (Mito) fractions for analysis of Cyt c levels by Western blotting. Protein levels were quantified using ImageJ (*n* = 3). **F** Representative TEM images showed the ultrastructure of neuronal mitochondria in the penumbra region of AAV-*R-CON* or AAV-*R1SR13* mice after Sham or MCAO 24 h. Yellow arrows indicate neuronal mitochondria (*n* = 6–7). Scale bar: 1 μm. **G** SH-SY5Y cells were transfected with pU6-R-CON or pU6-R1SR13, subjected to OGD for 0 or 6 h, and incubated in JC-1 working solution to detect JC-1 signal using fluorescence microscopy. Fluorescence intensity was measured using ImageJ. Scale bar: 20 μm (*n* = 3). **H** R1SR13 and negative control SH-SY5Y cell lines were treated with OGD for 0 or 6 h. Cells were harvested to detect ROS levels using flow cytometry. Mean fluorescence intensity of ROS signal was quantified using the FlowJo software (*n* = 3). **I** R1SR13 and negative control SH-SY5Y cell lines were treated with OGD for 0 or 6 h. Cells were harvested to detect mtND1 levels using RT-qPCR (*n* = 4). **J** RNA pull-down assay showed that R1SR13 and *ATF2* mRNA competitively bind to RCAN1.1L-56-158. The recombinant protein RCAN1.1L-56-158-6His was incubated with R1SR13 or its mutant (at varying concentrations), followed by pull-down using biotin-labeled *ATF2* mRNA A3-B2-C3 (2915-2935 nt). Western blotting was used to detect RCAN1.1L-56-158 using anti-His antibody. **K** EMSA revealed competitive binding between R1SR13 and *ATF2* mRNA for the RCAN1.1L-56-158 domain. **L** RNA scope in situ hybridization revealed the localization of *Atf2* mRNA on mitochondria in mice following microinjection of AAV-*R-CON* or AAV-*R1SR13* after Sham or MCAO for 24 h. Mitochondrial *Atf2* mRNA fluorescence intensity was measured using ImageJ (*n* = 7–8). Scale bar: 20 μm. **M** R1SR13 and negative control SH-SY5Y cells were treated with OGD for 0 or 6 h. Cells harvested and biochemically fractionated into mitochondrial fractions. *ATF2* mRNA levels were detected using RT-qPCR (*n* = 4). **N** R1SR13 and negative control SH-SY5Y cells were harvested for WC or Mito fractions after OGD for 0 or 6 h. ATF2 protein levels were detected using Western blotting. Protein quantification was conducted using ImageJ (*n* = 3). **O** For immunofluorescence, R1SR13 and negative control SH-SY5Y cells were treated with OGD for 0 or 6 h. Colocalization between MitoTracker and ATF2 protein was examined and intensity profiles of the indicated proteins were analyzed using line scan analysis in ImageJ. Images were captured using an LSM980 microscope. Scale bar: 5 μm. Data presented as mean ± SEM. *P < 0.05, **P < 0.01, ***P < 0.001.

R1SR13 is an in vitro high-affinity aptamer selected via the SELEX assay, capable of specifically binding to the RCAN1.1L protein and inhibiting its biological function [15]. Due to its specificity and minimal off-target effects, R1SR13 was selected to competitively disrupt the RCAN1.1L-*ATF2* mRNA interaction. Such aptamers offer distinct advantages over conventional therapeutic agents, including ease of synthesis, favorable tissue penetration, thermal stability, and adaptability to chemical modifications for targeted delivery [63, 64]. AAV9-*R1SR13*-GFP (AAV-*R1SR13*) was constructed by inserting the R1SR13 fragment into the AAV9 vector, and the expression efficiency of both AAV-*R1SR13* and the control virus (AAV-*R-CON*) was confirmed following microinjection into the mouse cerebral cortex. RCAN1.1L levels remained unchanged in R1SR13-treated mice (Fig. S7A). MRI showed that R1SR13 significantly reduced cerebral infarction volume after MCAO 24 h (Figs. 7A and S7B). In the mouse MCAO model, R1SR3 significantly improved neurological score and motor function compared to the control group (Figs. 7B, C and S7C). Immunofluorescence revealed decreased cleaved caspase-3, and cleaved caspase-9 levels in the penumbra of MCAO mice treated with R1SR13 (Fig. S7D, E). In stably-infected SH-SY5Y cell lines overexpressing R1SR13 and control cell lines, R1SR13 did not alter *RCAN1.1L* mRNA or protein levels (Fig. S7F, G). The results showed that R1SR13 reduced the release of Cyt c from mitochondria into the cytoplasm, and decreased the protein levels of FIS1, cleaved caspase-3, and cleaved caspase-9 after OGD (Fig. 7D, E).

TEM showed that R1SR13 mitigated mitochondrial morphological impairment 24 h post-MCAO in both mouse brain tissue (Fig. 7F) and SH-SY5Y cells (Fig. S7H). Under OGD conditions, Seahorse analysis revealed that R1SR13 treatment significantly rescued impaired cellular oxidative respiration capacity compared to the control group (Fig. S7I). Western blotting analysis indicated that R1SR13 treatment significantly attenuated the loss of mitochondrial complex I-IV proteins after OGD 6 h compared to the control group (Fig. S7J). In addition, the improvement in Δψ_m_ was corroborated by JC-1 immunofluorescence (Fig. 7G). Mitochondrial Ca²⁺ fluorescence imaging showed that R1SR13 treatment markedly attenuated mitochondrial Ca²⁺ accumulation following OGD 6 h compared to the control group (Fig. S7K). Furthermore, R1SR13 treatment reduced ROS production (Fig. 7H) and increased mtDNA levels (Fig. 7I) in SH-SY5Y cells subjected OGD 6 h.

**Fig. 8:**
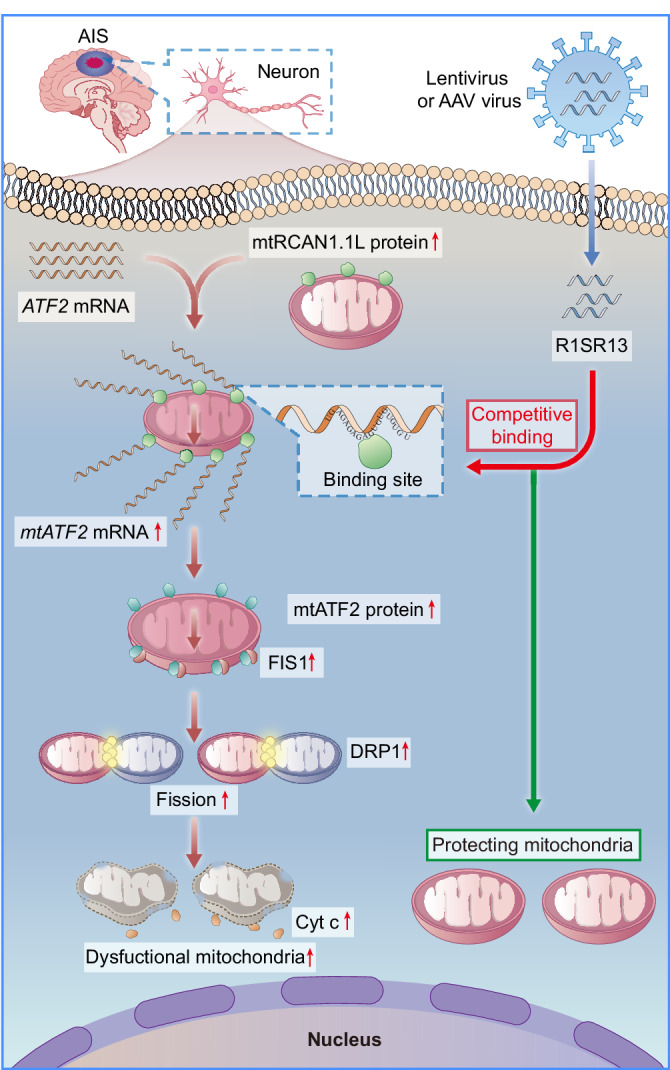
RBP RCAN1.1 L modulates *ATF2* mRNA stability to promote mitochondrial fission in AIS. In an AIS animal model, RCAN1.1L was upregulated in the ischemic penumbra promoted cerebral infarct enlargement and exacerbated neurological deficits.‌ Under ischemic conditions, RCAN1.1L binds and stabilizes *ATF2* mRNA translocation to the OMM in penumbra neurons. The pathogenic RCAN1.1L-mtATF2-FIS1 axis drives mitochondrial fission and apoptosis. R1SR13 improves neurological outcomes by interfering with RCAN1.1L-*mtATF2* mRNA stabilizing activity in preclinical AIS models.

Mechanistically, RNA pull-down assays demonstrated that increased R1SR13 levels competitively reduced the ability of biotin-labeled *ATF2* mRNA to pull down RCAN1.1L 56-158, while mutant R1SR13 did not affect this interaction (Fig. 7J). The competitive binding results were further validated by RNA EMSA (Fig. 7K). RNA scope in situ hybridization revealed colocalization of *Atf2* mRNA with mitochondria in the penumbra of AAV-*R-CON* mouse cerebral cortex, while, R1SR13 significantly diminished this colocalization (Fig. 7L). Furthermore, R1SR13 treatment decreased mitochondrial *ATF2* mRNA levels in SH-SY5Y cells after OGD 6 h (Fig. 7M). MtATF2 protein levels were significantly decreased in R1SR13-treated SH-SY5Y cells after OGD 6 h (Fig. 7N). Additionally, ATF2 colocalized with MitoTracker at OGD 6 h, but not in R1SR13-treated cells (Fig. 7O). Therefore, R1SR13 protects mitochondrial function in AIS, thereby reducing intrinsic apoptosis and adverse outcomes. Based on the above research results, R1SR13 protective mechanism may involve competitive binding of *ATF2* mRNA to RCAN1.1L protein, hindering ATF2-mediated RCAN1.1L promotion of the intrinsic apoptotic pathway (Fig. 8).

## Discussion

This study demonstrates for the first time that mtRCAN1.1L protein expression is significantly elevated in AIS models. Moreover, we identified that RCAN1.1L specifically binds to 2915-2935 nt in the 3’-UTR of *ATF2* mRNA, thereby stabilizing the transcript and promoting mtATF2 protein accumulation. Furthermore, our data reveal that mtATF2 physically interacts with FIS1, driving pathological mitochondrial fission. Most significantly, we established that the RCAN1.1L-mtATF2-FIS1 signaling activates intrinsic apoptosis and exacerbates cerebral infarction progression in AIS. These findings provide novel molecular insights into AIS pathogenesis through characterization of this previously unrecognized regulatory pathway.

RCAN1.1 is a multifunctional regulatory protein critically involved in diverse cellular processes, including mitochondrial dysfunction, proliferation, apoptosis, inflammation, synaptic expression and plasticity, and transcriptional regulation [65–67]. Our previous investigations revealed significant upregulation of *RCAN1.1L* mRNA and protein in ischemic brain tissue [12]. In the current study, we employed high-resolution MRI coupled with Nissl staining to precisely map mtRCAN1.1L protein upregulation within the cortical ischemic penumbra. We also identified elevated plasma RCAN1.1 levels in AIS patients firstly, likely reflecting increased brain tissue expression due to blood-brain barrier disruption during ischemia [68, 69]. ROC analysis indicating RCAN1.1 as a potential biomarker for the early diagnosis of AIS. This molecularly and clinically innovative approach provides unprecedented spatial resolution and robust experimental evidence supporting the pivotal role of RCAN1.1L in AIS pathogenesis.

Mitochondrial dysfunction is recognized as an initial event and marker of neuronal death caused by AIS, and preserving mitochondrial homeostasis is crucial for the survival of neurons and the enhancement of neurological function [30, 70]. Our preliminary research has demonstrated that HIF-1α can specifically combine and activate the functional hypoxia-responsive element (HRE) site (−325 to −322 bp) in the *RCAN1.1* gene promoter. Furthermore, we found that increased expression of RCAN1.1L exacerbates neuronal apoptosis, as indicated by TUNEL, reduced ATP levels, increased caspase3/7 activities in AIS [12]. ‌This study demonstrates that RCAN1.1L exacerbates MCAO-induced brain injury by promoting mitochondria dysfunction, as manifested by decreased Δψ_m_, reduced mtDNA copy number, increased ROS production, and ultimately initiates the intrinsic apoptotic pathway. Notably, inhibition of RCAN1.1L significantly attenuated the cerebral infarct size by mitigating this mitochondria-dependent apoptotic pathway.‌

RBPs regulate virtually every aspect of RNA metabolism, from transcriptional processing and nuclear export to cytoplasmic localization, translational control, and ultimately RNA degradation [19, 20, 71, 72]. To date, nearly 1000 mammalian RBPs have been reported to bind RNA through specialized globular RBDs [21]. In this study, we found that the RBP RCAN1.1L binds to human *ATF2* mRNA by RNA pull-down assay, RNA EMSA, and SPR analysis. In AIS models, RNA scope and RT-PCR assay following mitochondrial purification showed that upregulation of mtRCAN1.1L is accompanied by increased levels of *mtATF2* mRNA. ATF2 is a nuclear gene-encoded protein whose mRNA is transcribed in the nucleus and subsequently exported to the cytoplasm for translation [26, 73]. Accumulating evidence indicates that ATF2 can localize to mitochondria, where it accumulates and promotes mitochondrial apoptosis [26, 74]. This study represents the first demonstration of this process in models of AIS. Meanwhile, the molecular mechanisms underlying the specific targeting of ATF2 to mitochondria remain poorly understood, particularly whether such localization is regulated through post-transcriptional or post-translational pathways, warranting comprehensive investigation. Currently, research has shown that RBP not only regulates the mRNA responsible for encoding mitochondrial proteins and aids in their transport and translation, but also facilitates the transportation and anchoring of mRNA to the surface of mitochondria through direct interaction or by binding to TOM complex components [75]. In this process, mitochondrial targeting signals (MTS), derived from both the coding region and the 3’ UTR of mRNAs, have been demonstrated to play a crucial role in directing mitochondrial localization [76, 77]. RBPs play a critical role in this process by ensuring proper mRNA localization for efficient translation [18, 78]. Based on our data, we propose that RCAN1.1L may form RNA granules or transport machinery to deliver *ATF2* mRNAs to mitochondria, thereby providing novel evidence for the post-transcriptional regulation of mtATF2 localization. This reveals a post-transcriptional network where RCAN1.1L influences mitochondrial homeostasis and metabolism [79, 80]. Although our understanding of regulatory mechanisms controlling directed transport and spatio-selective stabilization of transcripts is still limited. However, this study, similar to investigations of localized neuronal mRNAs such as CamK2A and Bassoon, sharpens our understanding of how RBP-mRNA interactions determine specific localization patterns [78].

RBPs undergo dynamic remodeling within cellular environments and exhibit dual functionality in mRNA homeostasis [21]. Beyond stabilizing target transcripts, they actively direct aberrant or mislocalized mRNAs to lysosomes for degradation, thereby executing critical quality control [81]. In this study, we discovered that RCAN1.1L significantly enhances the stability of *ATF2* mRNA‌, consistent with the regulatory roles of established RBPs such as ‌TDP43, HuR, and hnRNPD [82, 83]. More precisely, we mapped the ‌RCAN1.1L protein-binding site‌ to ‌nucleotides 2915-2935 within the 3′ UTR of *ATF2* mRNA using AlphaFold 3 prediction and RNA EMSA‌. The 3′-UTR regulates targeting, translational efficiency, and stability of mRNA [84]. Extended 3′-UTRs have been documented in neuronal tissue in both mouse and human samples [85]. Consistent with previous findings, the 3’-UTR represents the predominant regulatory region for ATF2 mRNA binding. The RNA-binding protein HuR, along with specific microRNAs such as miR-26a/b and miR-204, interacts with the 3’-UTR of *ATF2* mRNA to modulate its stability and degradation [86]. The interaction between the RNA-binding protein RCAN1.1L and *ATF2* mRNA prolongs the half-life of *ATF2* mRNA, revealing a novel post-transcriptional regulatory mechanism of ATF2 in AIS.

Neurons rely on the transport of mRNA and local translation to enable rapid protein synthesis in cellular compartments distant from the soma [87]. With the exception of a few mitochondrial proteins and RNAs encoded by mitochondrial DNA, the majority of mitochondrial proteins are synthesized from mRNAs transcribed from nuclear DNA. For a subset of these mRNAs, co-translational import across the OMM occurs through interactions with TOM70 [88]. RBPs recruit ribosomes to the OMM and binds to mRNAs on the mitochondrial surface, thereby initiating the translation process [89]. Previous research reported that PINK1 interacts with Pumilio (Pum) and the TOM/TIM complex to regulate the localization and translation of *nuclear-encoded respiratory chain component (nRCC)* mRNAs to mitochondria [90]. Our results reveal RCAN1.1L overexpression drives pronounced ATF2 protein enrichment within mitochondria across in vitro and in vivo. Conversely, ‌RCAN1.1L knockdown concomitantly abolishes this mitochondrial accumulation‌, establishing a causal role for RCAN1.1L in regulating ATF2 subcellular localization. Based on the findings of this study, *ATF2* mRNA carried by RCAN1.1L is stabilized and anchored on mitochondria. Given that mRNAs can undergo direct translation on the OMM, newly synthesized proteins derived from these transcripts are capable of interacting with specific OMM proteins, thereby facilitating co-translational import and the completion of translation at the mitochondrial surface [91]. We therefore propose that the upregulation of mitochondrial ATF2 protein results from its local translation at the OMM [78, 92]. Our study provides evidence supporting the occurrence of local translation of nuclear-encoded proteins within mitochondria. However, further investigation is required to elucidate the underlying molecular mechanisms governing *ATF2* mRNA translation on mitochondria.

Co-IP and LC-MS analyses confirm that mtATF2 and FIS1 co-localize under OGD conditions. Upregulated mtATF2 enhances FIS1 expression, activating DRP1 to promote mitochondrial fission, which represents a novel regulatory mechanism of mitochondrial fission [93, 94]. Our study elucidates the multifaceted regulation of ATF2 on mitochondrial function. Following genotoxic stress, ATF2 translocates to the OMM, disrupts HK1/VDAC1 complexes, compromises mitochondrial integrity, and sensitizes cells to apoptosis [85]. Similar to DRP1, FIS1 also serves as a marker protein in mitochondrial fission [95, 96]. Although initially thought to be the OMM receptor recruiting cytoplasmic DRP1, recent studies have redefined its role, showing that FIS1 predominantly mediates pathological peripheral fission [31, 36, 97]. Mitochondrial fission causes mitochondria to fragment shortly before apoptosis, and inhibiting fission prevents Cyt c release and delays intrinsic apoptosis [98, 99]. Overall, we provided evidences for the first time that ATF2 could directly interact with FIS1, induce mitochondrial fission and promote Cyt c release, increased cleaved caspase-9 and cleaved caspase-3, which finally led to mitochondrial dysfunction and apoptosis in AIS. Thus, our results suggest that RCAN1.1L played a pathogenic role on mitochondria by regulating ATF2 in AIS.

R1SR13 competitively inhibits RCAN1.1L-*ATF2* mRNA interactions in vivo, thereby disrupting the formation of functional complexes and providing mitochondrial neuroprotection. As a precision medicine approach, R1SR13 packaged with blood-brain barrier-penetrating carriers administered pre-perfusion represents a promising strategy. Future trials will explore single or combined neuroprotective strategies to widen the therapeutic window [100]. Nerinetide, a peptide inhibiting PSD-95 cytotoxicity, is an example currently tested in ambulance-based trials [7, 101]. Despite promising preclinical data, clinical translation remains challenging [8, 100]. Future studies should focus on developing R1SR13 into a small molecule drug suitable for immediate post-stroke administration, thereby maximizing neuroprotection and extending the recanalization time.

Although this study is the first to demonstrate that the RBP RCAN1.1L promotes mitochondrial fission and induces apoptosis through post-transcriptional regulation of *ATF2* mRNA, and to elucidate the mitochondrial protective effects of its inhibitory aptamer R1SR13, certain limitations remain. Limitations include exclusive use of male mice, necessitating exploration of sex-specific differences [102, 103]. Moreover, cognitive function could not be reliably assessed in the pMCAO model due to motor impairment confounding standard behavioral tests such as the Morris water maze and Y-maze [104]. Additionally, the limited size of our human cohort precluded establishing a causal relationship between plasma RCAN1.1L levels and penumbra volume, underscoring the importance of larger studies. The mechanism of RCAN1.1L-mediated mRNA transport to mitochondria and local ATF2 protein translation remains unclear, representing a key area for future research.

In conclusion, this work redefines RCAN1.1L-*mtATF2* mRNA interaction as both a pathogenic driver and therapeutic target in AIS by bridging mitochondrial biology and post-transcriptional regulation. The RCAN1.1L-mtATF2-FIS1 axis expands understanding of mitochondrial fission and apoptosis, providing a blueprint for developing R1SR13 as neuroprotectants.

## Supplementary information

Supplementary Materials

Figure S1

Figure S2

Figure S3

Figure S4

Figure S5

Figure S6

Figure S7

Data S1 LC-MS data

Full and uncropped western blots

## Data Availability

The data that support the findings of this study are available from the corresponding author upon reasonable request.
