# Supplementary material for: RNA-binding protein RCAN1.1L modulates ATF2 mRNA stability to promote mitochondrial fission in acute ischemic stroke
Source: Cell Death Dis. 2026 May 13;17(1):621. doi: 10.1038/s41419-026-08809-8 (PMC13342642; doi:10.1038/s41419-026-08809-8)
Supplement: Supplementary file 1 — Supplementary Materials [file 41419_2026_8809_MOESM1_ESM.docx]

Supplemental Material for

**RNA-binding protein RCAN1.1L modulates *ATF2* mRNA stability to promote mitochondrial fission in acute ischemic stroke**

**CONTENT**

Figures S1-7

Tables S1-6 and Legends for Data S1.

**Fig. S1 Establishment of mouse pMCAO model and sample collection from AIS patients and healthy controls.**

(**A**) Representative images of MRI angiography and T2WI from sham and MCAO mice. (**B, C**) *RCAN1.1L* mRNA (B) and protein (C) levels were detected in the shRCAN1.1 and corresponding control cell lines via RT-qPCR and Western blotting (n=3-4). (**D**) Experimental design for identifying RCAN1.1 protein in AIS patients. (**E**) A total of 34 HCs and 72 AIS patients were recruited for this study. The AIS cohort was stratified based on the time from symptom onset into three subgroups: <6 h, 6-24 h, and 24 h to 2 weeks. Data presented as mean ± SEM. **P*<0.05, ***P*<0.01, ****P*<0.001.

**Fig. S2 AAV9-mediated overexpression in mouse cortex: induction of neurological dysfunction and apoptosis by RCAN1.1L after MCAO.**

(**A**) Representative image showed efficient infection of mouse brain by AAV9-*RCAN1.1L* virus. (**B**) Western blotting revealed that RCAN1.1L protein was detected in the cerebral cortex of mice with stereotaxic injection of AAV-*CON*, AAV-*RCAN1.1L*, AAV-*shCON* and AAV-*shRCAN1.1* viruses (n=8-9). (**C**) Real-time CBF was assessed by LSCI in control and AAV-*RCAN1.1L* mice following sham or MCAO surgery. (**D**) Representative T2WI data from AAV-*RCAN1.1L* and control mice subjected to sham for 24 h (n=6). (**E, F**) The percentage of left turn was measured using Corner test in AAV-*RCAN1.1L* (E), AAV-*shRCAN1.1* (F), and their control mice after sham or MCAO (n=7-8). (**G, H**) Nissl staining of penumbral and core infarct tissues after sham or MCAO for 24 h in AAV-*CON*, AAV-*RCAN1.1L*, AAV-*shCON* and AAV-*shRCAN1.1* mice. Scale bars: 100 μm and 20 μm. (**I**) Immunofluorescent staining of the penumbra in the brain cortex of AAV-*CON* or AAV-*RCAN1.1L* mice subjected to sham or MCAO for 24 h with antibodies against cleaved caspase-3, and cleaved caspase-9. Images captured with LSM980. Fluorescence intensity of cleaved caspase-3, and cleaved caspase-9 proteins measured using ImageJ (n=6-7). Scale bar: 20 μm. (**J**) Representative image of GFP fluorescence in the RCAN1.1L cell lines. (**K, L**) *RCAN1.1L* mRNA (K) and protein (L) levels were detected in the RCAN1.1L and corresponding control cell lines via RT-qPCR and Western blotting (n=3-4). (**M**) shRCAN1.1 and shCON cell lines were harvested after OGD 0 or 6 h to detect cleaved caspase-3, and cleaved caspase-9 proteins via Western blotting analysis (n=3). Data presented as mean ± SEM. **P*<0.05, ***P*<0.01, ****P*<0.001.

**Fig.S3 The impact of RCAN1.1L on mitochondrial function in an AIS cellular model.**

(**A, B**) SH-SY5Y cell lines expressing RCAN1.1L- (A), shRCAN1.1- (B), and control cell lines were treated with or without OGD. Ultrastructure of mitochondria observed via TEM. Yellow arrows indicate neuronal mitochondria (n=4). Scale bar: 1 μm. (**C-E**) The OCR of RCAN1.1L- (C), shRCAN1.1- (E), and control cell lines, with or without OGD treatment, was analyzed using a Seahorse analyzer under metabolic stress induced by sequential injection of oligomycin, FCCP, antimycin A, and rotenone (n=3). (**F, G**) Western blotting analysis of mitochondrial respiratory complex subunits (UQCRC1, SDHB, MTCO2, and NDUFB8) in RCAN1.1L- (F), shRCAN1.1- (G), and control cell lines with or without OGD exposure (n=3). (**H, I**) Mitochondrial Ca²⁺ levels in RCAN1.1L- (H), shRCAN1.1- (I), and control cell lines, with or without OGD treatment, were measured using the fluorescent probe Rhod-2 AM and quantified as mean fluorescence intensity with ImageJ software (n=4). (**J**) SH-SY5Y cells were harvested after OGD 0 or 6 h to detect ROS levels via flow cytometry (n=4). Data presented as mean ± SEM. **P*<0.05, ***P*<0.01, ****P*<0.001.

**Fig. S4 Role of RCAN1.1L in the regulation of mitochondrial biogenesis in an AIS cellular model.**

(**A-B**) PGC-1α protein levels were determined by Western blotting in RCAN1.1L- (A), shRCAN1.1- (B), and control cell lines with or without OGD 6 h exposure (n=3). (**C**) SH-SY5Y cells were treated with OGD for indicated times (0-12 h), followed by harvesting to detect mtND1 levels via RT-qPCR (n=3). Data presented as mean ± SEM. **P*<0.05, ***P*<0.01, ****P*<0.001.

**Fig. S5 Experimental design for RNA Sequencing and selection of Motif CM-1 from CLIP-seq data.**

(**A**) Detailed experimental process to identify *ATF2* mRNA binding to RCAN1.1L protein based on CLIP-seq, mitochondrial transcriptomics data, and RIP-seq. Created in BioRender. ji, Y. (2026) https://BioRender.com/hwxfxil. (**B**) Motif CM1 derived from HOMER de novo motif analysis of the RCAN1.1L protein binding peak based on CLIP-seq data.

**Fig. S6 Verification of ATF2 overexpression or knockdown in SH-SY5Y cells and RCAN1.1L on mitochondrial fusion under OGD condition.**

(**A**) Representative image of RFP fluorescence in the ATF2 cell lines. (**B, C**) *ATF2* mRNA (B) and protein (C) levels were detected in ATF2 and corresponding control cell lines via RT-qPCR and Western blotting (n=3-4). (**D**) The ATF2 and negative control cell lines were collected for fractionation. WC or Mito fractions analyzed for ATF2 protein levels via Western blotting (n=3). (**E**) Cycloheximide (CHX) was added to ATF2 and control cell lines at a final concentration of 100 μg/mL. Cells were then collected at 0, 12, 24, and 36 h post-treatment, and FIS1 protein levels were analyzed by Western blotting (n=3). (**F, G**) After transient transfection with *siCON* or *siATF2* for 48 h, *ATF2* mRNA (F) and protein (G) levels were detected in SH-SY5Y cells via RT-qPCR and Western blotting (n=3-4). (**H**) OPA1 and MFN1 protein levels were determined by Western blotting in RCAN1.1L and control cell lines, both with and without OGD treatment (n=3). (**I**) RCAN1.1L stable cell lines were transfected with control siRNA (siCON) or ATF2-targeting siRNA (siATF2) for 48 h, followed by OGD 6 h. Protein levels of PGC-1α, OPA1, and MFN1 were assessed by Western blotting (n=3). (**J**) RCAN1.1L stable cell lines were transfected with control siRNA (siCON) or ATF2-targeting siRNA (siATF2) for 48 h, followed by OGD 6 h. Cells were harvested and analyzed for mtND1 levels via RT-qPCR (n=4). Data presented as mean ± SEM. **P*<0.05, ***P*<0.01, ****P*<0.001.

**Fig. S7 R1SR13 attenuates mitochondrial impairment and apoptosis in an AIS Model.**

(**A**) The RCAN1.1L protein was detected in the cerebral cortex of mice via Western blotting after stereotaxic injection of AAV-*R-CON* or AAV-*R1SR13* viruses (n=9). (**B**) Representative T2WI data from AAV-*R-CON* and AAV-*R1SR13* mice subjected to Sham 24 h. (**C**) The percentage of left turn was measured using Corner test in AAV-*R-CON* and AAV-*R1SR13* mice after sham or MCAO (n=7-8). (**D, E**) Representative images of Cleaved caspase-3 and Cleaved caspase-9 protein in the penumbra from AAV-*R-CON* and AAV-*R1SR13* mice after Sham or MCAO (n=4). Scale bar: 5 μm. (**F, G**) *RCAN1.1L* mRNA (F) and protein (G) levels were detected in the R1SR13 and corresponding control cell lines via RT-qPCR and Western blotting (n=3-4). (**H**) R1SR13 and negative control SH-SY5Y cells were treated with OGD for 0 or 6 h. The ultrastructure of mitochondria was observed using TEM. Yellow arrows indicate neuronal mitochondria (n=4). Scale bar: 1 μm. (**I**) The OCR of R1SR13 and control cell lines, with or without OGD treatment, was analyzed using a Seahorse analyzer under metabolic stress induced by sequential injection of oligomycin, FCCP, antimycin A, and rotenone (n=3). (**J**) Mitochondrial Ca^2+^ levels in R1SR13 and control cell lines, with or without OGD treatment, were measured using the fluorescent probe Rhod-2 AM and quantified as mean fluorescence intensity with ImageJ software (n=4). (**K**) Western blotting analysis of mitochondrial respiratory complex subunits (UQCRC1, SDHB, MTCO2, and NDUFB8) in R1SR13 and control cell lines with or without OGD exposure (n=3). Data presented as mean ± SEM. **P*<0.05, ***P*<0.01, ****P*<0.001.

**Table S1 Demographic and clinical characteristics of** **HCs and AIS patients.**

|  | HCs group (n=34) | AIS group (n=77) | P-value |
| --- | --- | --- | --- |
| Male, n (%) | 22(64.7) | 48(62.3) | 0.493 |
| Age (years, Mean±SEM) | 67.12±1.45 | 67.35±1.29 | 0.915^‡^ |
| NIHSS score, median (IQR) | 0(0-0) | 9(5-12) | <0.001^†^ |
| Total cholesterol | 4.28±0.17 | 4.42±0.15 | 0.471^‡^ |
| High-density lipoprotein | 1.26(1.03-1.47) | 1.09(1.01-1.39) | 0.246^†^ |
| Low-density lipoprotein | 2.53±0.14 | 2.70±0.12 | 0.373^‡^ |
| Triglycerides | 0.94(0.72-1.44) | 1.12(0.90-1.52) | 0.113^†^ |
| **Medical history, n (%)** |  |  |  |
| Ischemic stroke | 1(2.9) | 17(22.1) | 0.008 |
| Hypertension | 11(32.4) | 44(57.1) | 0.013 |
| Diabetes | 12(35.3) | 25(32.5) | 0.510 |
| Coronary heart disease | 7(20.6) | 14(18.2) | 0.477 |
| Dyslipidemia | 1(2.9) | 3(3.9) | 0.641 |
| Others | 9(26.5) | 22(28.6) | 0.506 |

^†^ Mann-Whitney test was used. ^‡^ Student’s t test was used. The remaining data were analyzed utilizing the adjusted chi-squared test. NIHSS, National Institutes of Health Stroke Scale. IQR, interquartile range. Others, Trauma and orthopedic surgery greater than 3 months, chronic gastritis, rheumatoid arthritis and other diseases.

**Table S2 ﻿Primer sequences for RT-PCR.**

| Gene | Primer sequences |
| --- | --- |
| Homo-ATF2 | Forward: CCAGCGTTTTACCAACGAGG |
|  | Reverse: TGTCATTACGTGCTGGACCAA |
| Homo-RCAN1.1L | Forward: TGGAGCTTCATTGACTGCGA |
|  | Reverse: CAGGTGACAGGCGATGGTG |
| Human ANT1 | Forward: ACGGGCTACTACAACCCTTC |
|  | Reverse: ATGGTAGATGTGGCGGGTTT |
| Human mtND1 | Forward: ACGGGCTACTACAACCCTTC |
|  | Reverse: ATGGTAGATGTGGCGGGTTT |
| Human D-Loop | Forward: ATCCCGCACAAGAGTGCTAC |
|  | Reverse: GGGGAACGTGTGGGCTATTT |
| Human S14 | Forward: GGCAGACCGAGATGAATCCTC |
|  | Reverse: CAGGTCCAGGGGTCTTGGTCC |
| Human U2 | Forward: CATCGCTTCTCGGCCTTTTG |
|  | Reverse: TGGAGGTACTGCAATACCAGG |
| Human β-actin | Forward: GACAGGATGCAGAAGGAGATTACT |
|  | Reverse: TGATCCACATCTGCTGGAAGGT |

**Table S3 Primer sequences for PCR in vitro RNA transcription.**

| Gene | Primer sequences |
| --- | --- |
| Homo ATF2-5UTR 1-296 | Forward: ATTTAGGTGACACTATAGAAGT  CAGTCCGATCTCGCGAGAGAGGA |
|  | Reverse: ATTCCACAGGTCCTTGTATTGCCGA |
| Homo ATF2-CDS 708-1067 | Forward: ATTTAGGTGACACTATAGAAGN  GTTACCTCACCCAGAGTCTAC |
|  | Reverse: CTAGGCACCATGGTGACTGGT |
| Homo ATF2-3UTR 2389-3221 | Forward: ATTTAGGTGACACTATAGAAGG  TAAAGACATACAGTGCTTTTATG |
|  | Reverse: GTACTGTATTTTGAACATAACTGCAG |
| Homo ATF2-3UTR 3693-4146 | Forward: ATTTAGGTGACACTATAGAAGN  GATTTTGATATCATTATTCTAAGG |
|  | Reverse: TAATTTAAAGATAGAATTTATTC |
| Homo ATF2-3UTR 2765-2864 | Forward: ATTTAGGTGACACTATAGAAGN  GCCCATTGTCTTGTACTTGCGA |
|  | Reverse: ATTTTTTTTTCCACGAGAAA |
| Homo ATF2-3UTR 2865-2963 | Forward: ATTTAGGTGACACTATAGAAGN  GCAGTATCTATCTTTAGAAC |
|  | Reverse: CACACACACACGCGCACACA |
| Homo ATF2-3UTR 2964-3077 | Forward: ATTTAGGTGACACTATAGAAGN  GTCTTTTAATAGTTTATGCCA |
|  | Reverse: TCCAAGCCTGCACAATAATG |

**Table S4 Cy3-labeled *ATF2* RNA specific sequences are employed in EMSA assays.**

| Name | Sequences |
| --- | --- |
| Homo ATF2-3UTR 2865-2891 | cy3-CAGUAUCUAUCUUUAGAACAAUGUAAU |
| Homo ATF2-3UTR 2892-2914 | cy3-UAUAAUGUGGGAAGUGUGCAUGA |
| Homo ATF2-3UTR 2915-2935 | cy3-AUGAGAGAGAGUGUGUGUGUA |
| Homo ATF2-3UTR 2936-2963 | cy3-UCUGUGUGUGUGUGCGCGUGUGUGUGUG |

**Table S5 Biotin-labeled *ATF2* RNA specific sequences and oligonucleotides are employed in RNA pull-down assays.**

| Name | Sequences |
| --- | --- |
| Biotin-*ATF2* RNA | Biotin-ACUCUCUCUCACACACACA |
| Biotin-*ATF2* RNA mutant | Biotin-UGAGAGAGAGUGUGUGUGU |
| R1SR13 | AUACAACAAAAACAAAAACAAGAAA |
| R1SR13mt | AUCACCACCCCCACCCCCACCUCAA |

**Table S6 ﻿Antibodies for immunoblot and immunofluorescence.**

| Antibodies | Category | Source |
| --- | --- | --- |
| Rabbit Polyclonal anti-RCAN1.1 (DCT3) |  | The antibodies produced by our research group |
| Rabbit Monoclonal anti-ATF2 | 35031S | Cell Signaling Technology |
| Mouse Monoclonal anti-Lamin B1 | 66095-1 | Proteintech |
| Rabbit Monoclonal anti- COX IV | 4850S | Cell Signaling Technology |
| Mouse Monoclonal anti- COX IV | 11967S | Cell Signaling Technology |
| Rabbit Monoclonal anti-β Tubulin | 2128S | Cell Signaling Technology |
| Mouse Monoclonal anti-TOM20 | 612278 | BD Transduction Laboratories |
| Human oxidative phosphorylation immunoblotting kit | PK30006 | Proteintech |
| Rabbit Polyclonal anti-Cytochrome c | 10993-1 | Proteintech |
| Rabbit anti-Cleaved caspase-3 | 9661T | Cell Signaling Technology |
| Rabbit Monoclonal anti-Cleaved caspase-3 | GB11532-100 | Servicebio |
| Rabbit Monoclonal anti-Cleaved caspase 9 | 20750S | Cell Signaling Technology |
| Rabbit Polyclonal anti-FIS1 | 10951-1 | Proteintech |
| Mouse Monoclonal anti-FIS1 | 66635-1 | Proteintech |
| Rabbit Polyclonal anti-DRP1 | 12957-1 | Proteintech |
| Rabbit Polyclonal anti-MFN1 | 13798-1-AP | Proteintech |
| Rabbit Polyclonal anti-OPA1 | 27733-1-AP | Proteintech |
| Rabbit Monoclonal anti-PGC-1 alpha | 2178T | Cell Signaling Technology |
| Mouse Monoclonal anti-flag | F1804 | Sigma Aldrich |
| Mouse IgG | A7082 | Beyotime |
| Mouse Monoclonal anti-myc | 60003-2 | Proteintech |
| Mouse Monoclonal anti-his | 66005-1 | Proteintech |
| Mouse Monoclonal anti-β actin | A5441 | Sigma Aldrich |
| Goat Anti-Mouse IgG H&L (Alexa Fluor® 647) | Ab150115 | Abcam |
| CoraLite488-conjugated Goat Anti-Rabbit IgG | SA00013-2 | Proteintech |
| CoraLite488-conjugated Goat Anti-Mouse IgG | SA00013-1 | Proteintech |
| CoraLite594-conjugated Goat Anti-Rabbit IgG | SA00013-4 | Proteintech |
| CoraLite594-conjugated Goat Anti-Mouse IgG | SA00013-3 | Proteintech |

**Data S1 158 mitochondrial proteins were identified as proteins that specifically interacted with mtATF2 under OGD 6 h, whereas no such interactions were observed under normoxic conditions by LC-MS data.**
