# Supplementary figures and images for: RNA-binding protein RCAN1.1L modulates ATF2 mRNA stability to promote mitochondrial fission in acute ischemic stroke
Source: Cell Death Dis. 2026 May 13;17(1):621. doi: 10.1038/s41419-026-08809-8 (PMC13342642; doi:10.1038/s41419-026-08809-8)

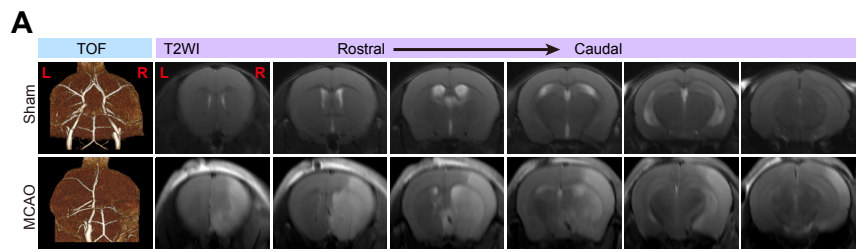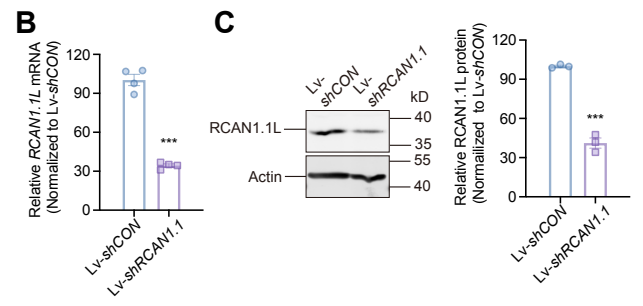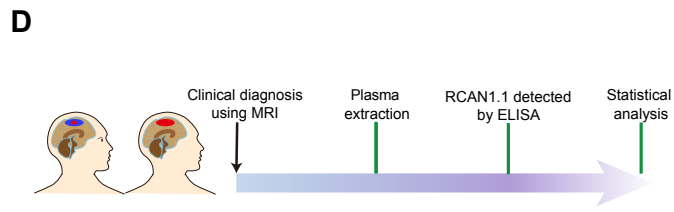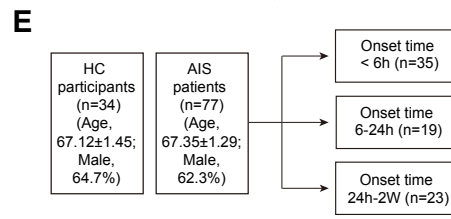

Supplement: Supplementary file 2 — Figure S1 [file 41419_2026_8809_MOESM2_ESM.pdf]

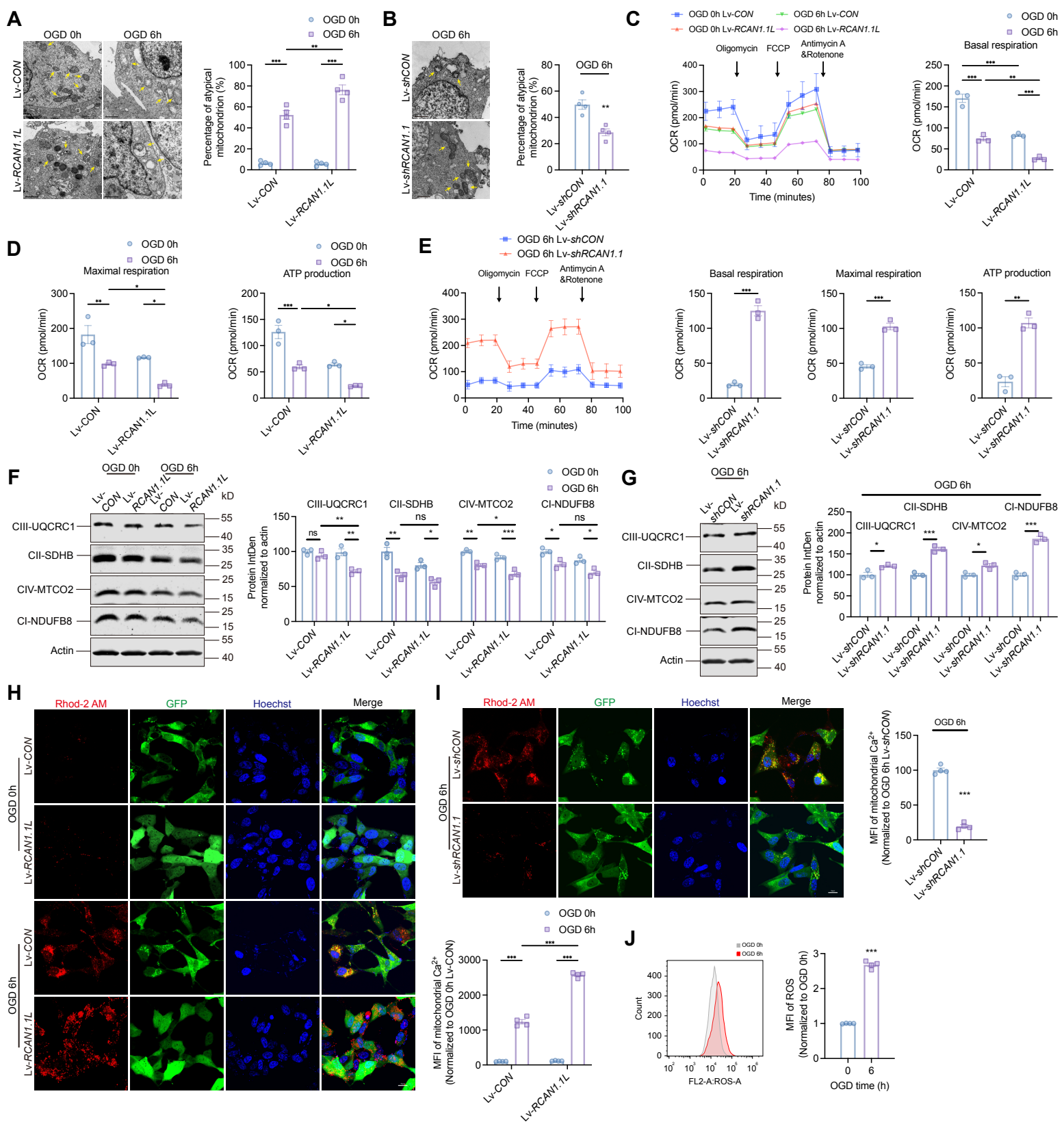

Supplement: Supplementary file 4 — Figure S3 [file 41419_2026_8809_MOESM4_ESM.pdf]

**A**

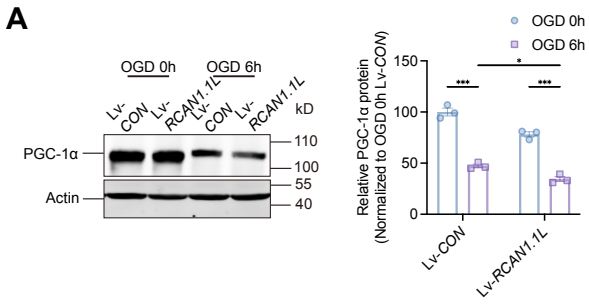

**B**

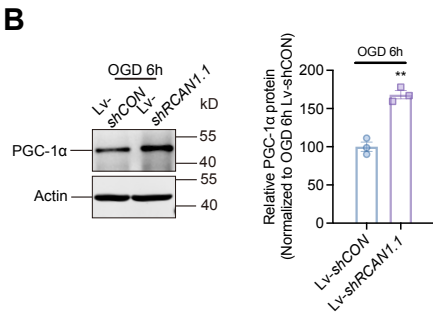

**C**

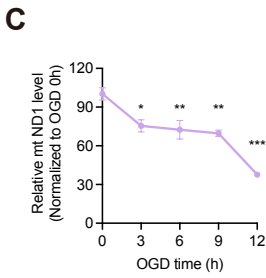

Supplement: Supplementary file 5 — Figure S4 [file 41419_2026_8809_MOESM5_ESM.pdf]

**A**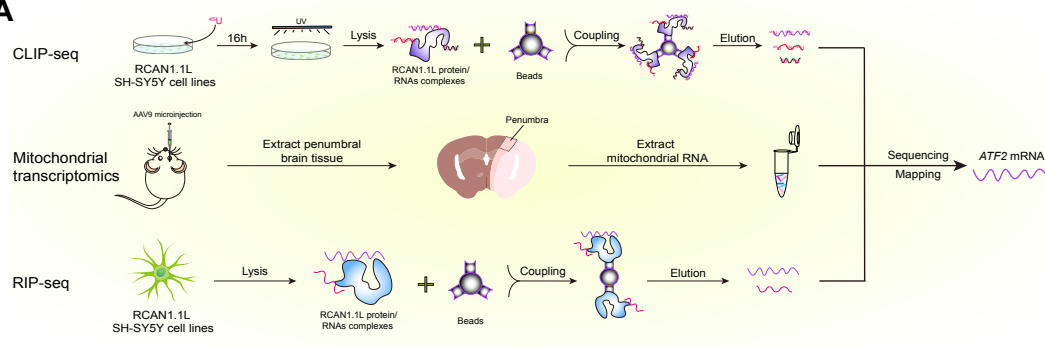**B**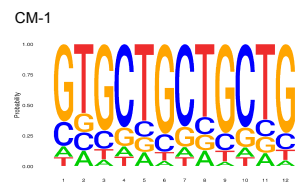

Supplement: Supplementary file 6 — Figure S5 [file 41419_2026_8809_MOESM6_ESM.pdf]

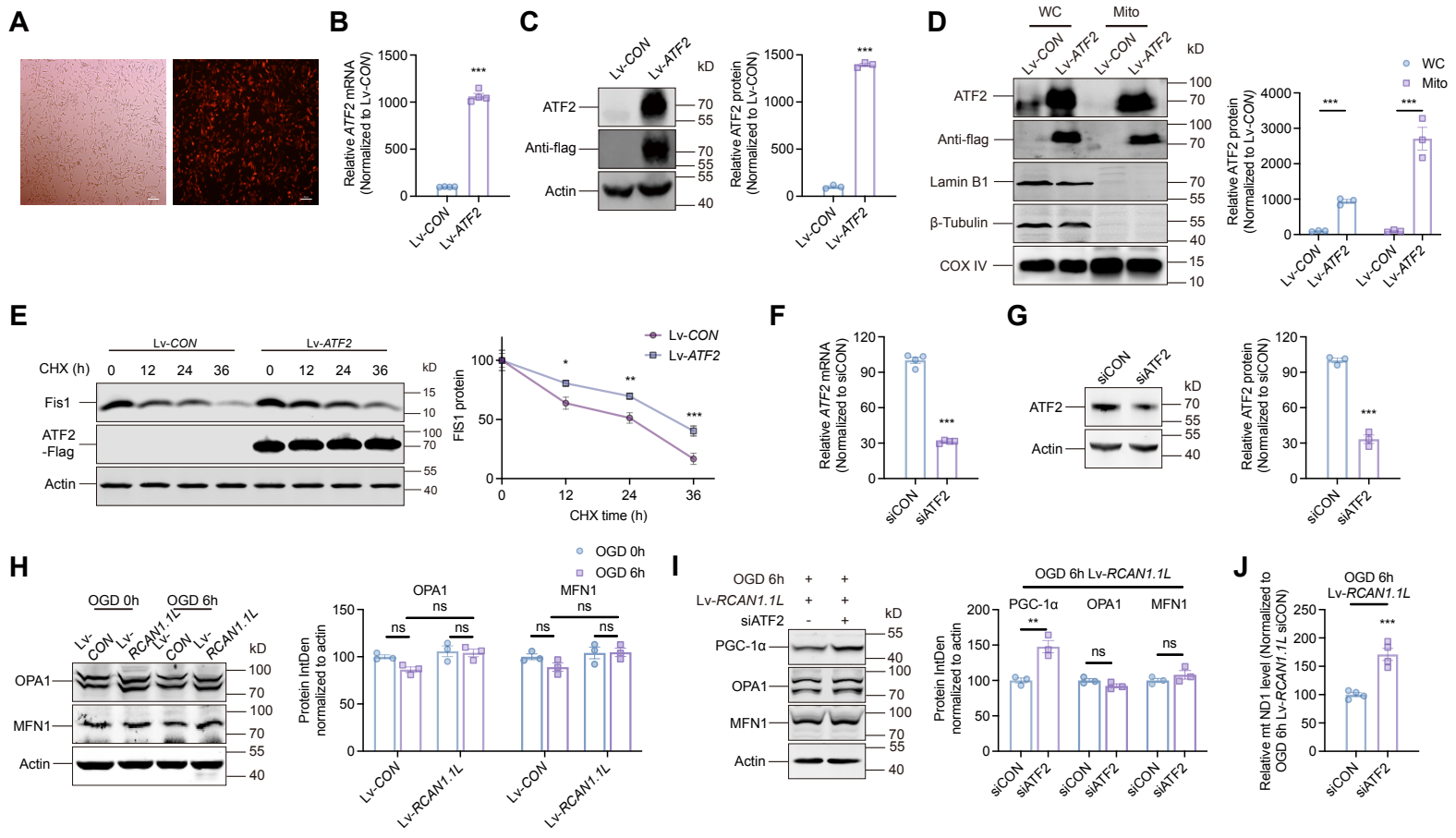

Supplement: Supplementary file 7 — Figure S6 [file 41419_2026_8809_MOESM7_ESM.pdf]
