# Supplementary material for: RNA-binding protein RCAN1.1L modulates ATF2 mRNA stability to promote mitochondrial fission in acute ischemic stroke
Source: Cell Death Dis. 2026 May 13;17(1):621. doi: 10.1038/s41419-026-08809-8 (PMC13342642; doi:10.1038/s41419-026-08809-8)

Full unedited blot for Fig. 1A

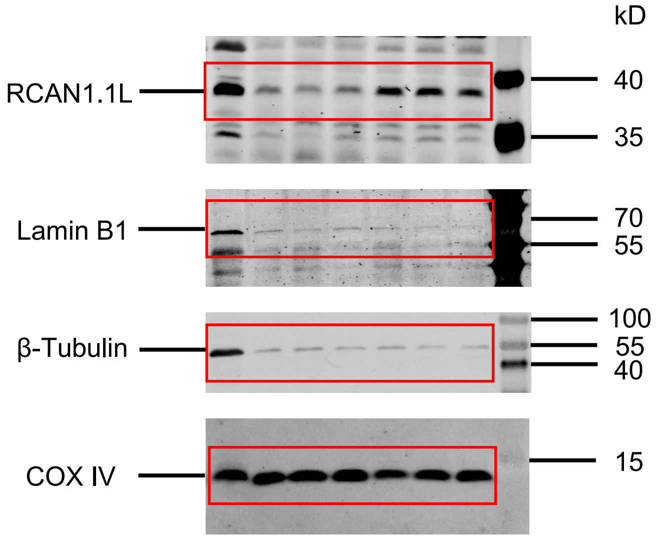

Full unedited blot for Fig. 1F

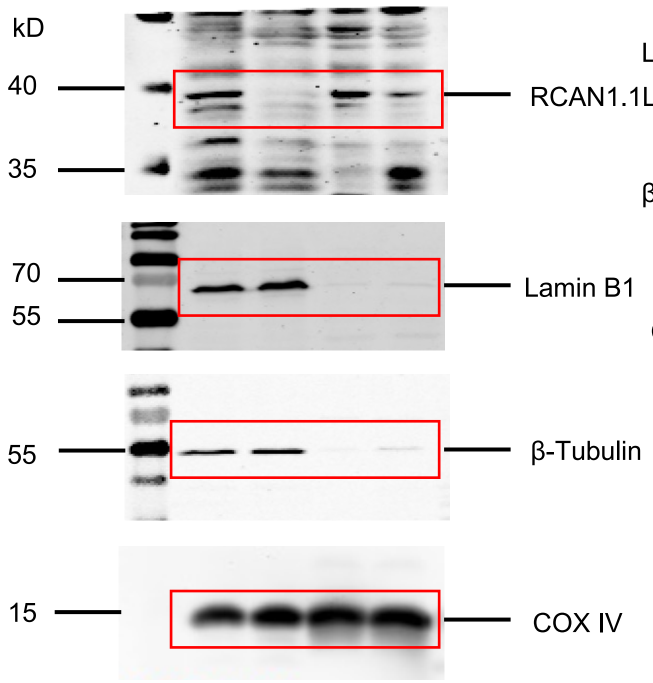

Full unedited blot for Fig. 1C

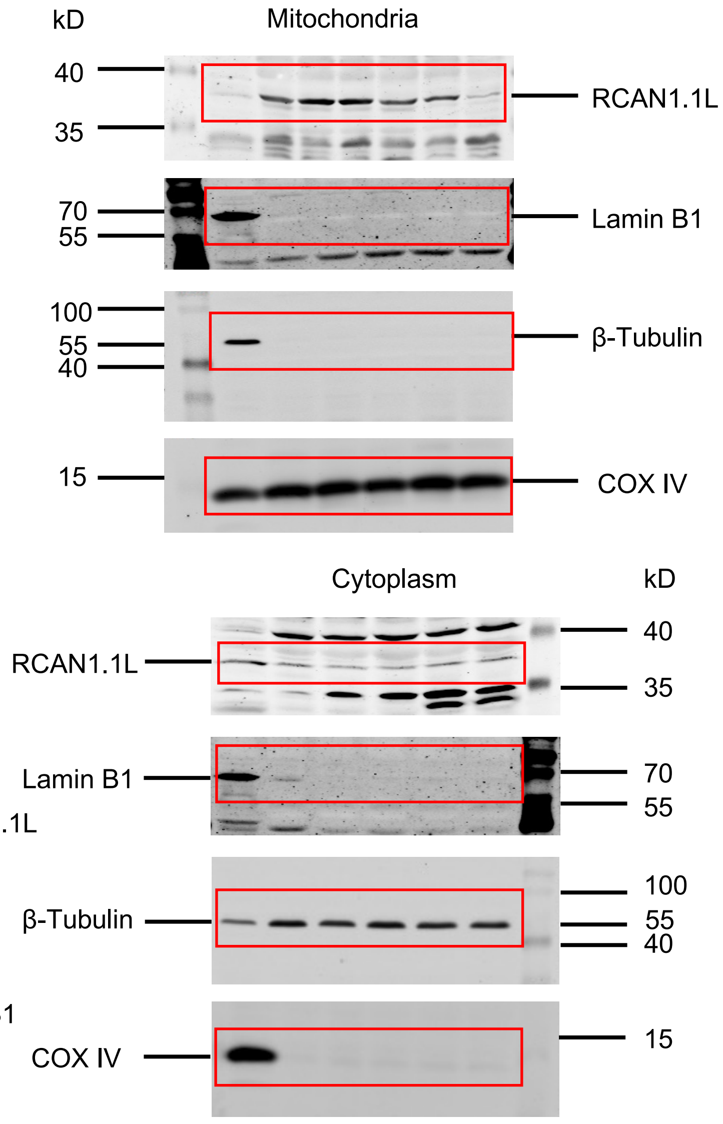

Full unedited blot for Fig. 2N

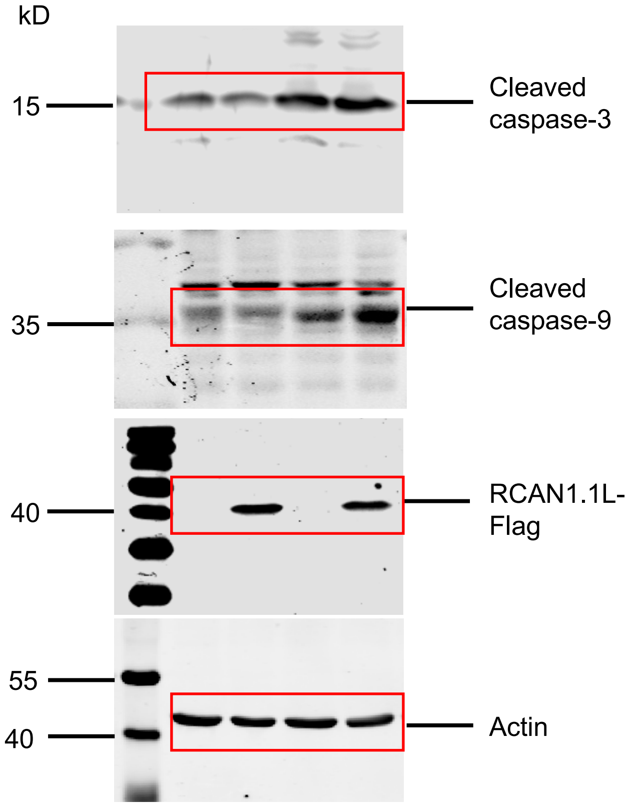

Full unedited blot for Fig. 2O

Left mitochondria

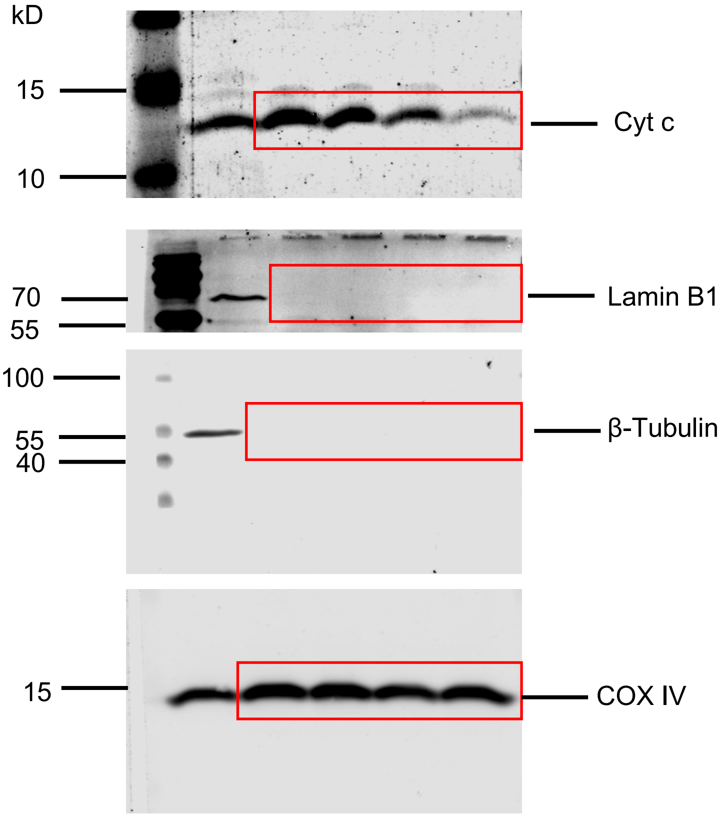

Full unedited blot for Fig. 2O

Left cytoplasm

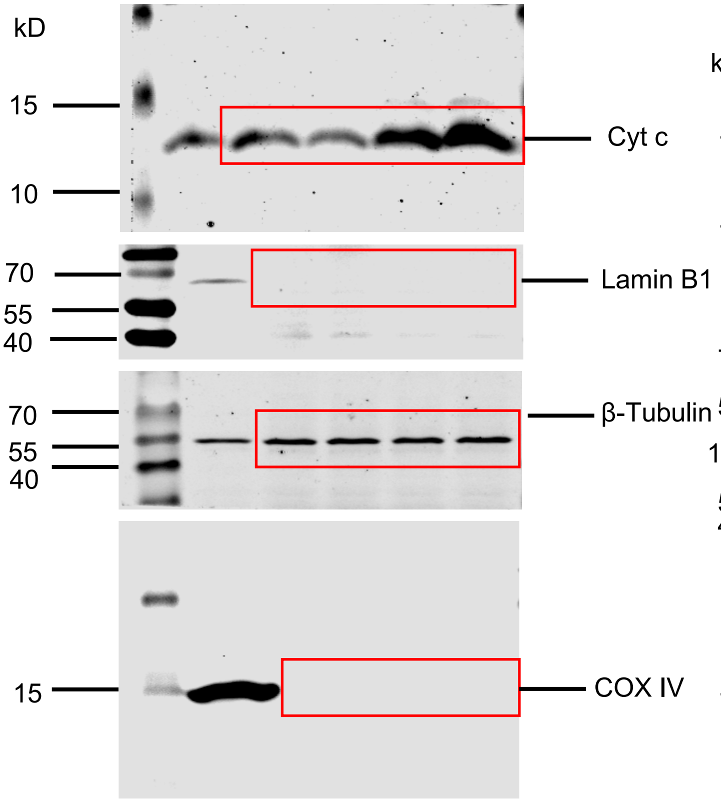

Full unedited blot for Fig. 2O

Right mitochondria

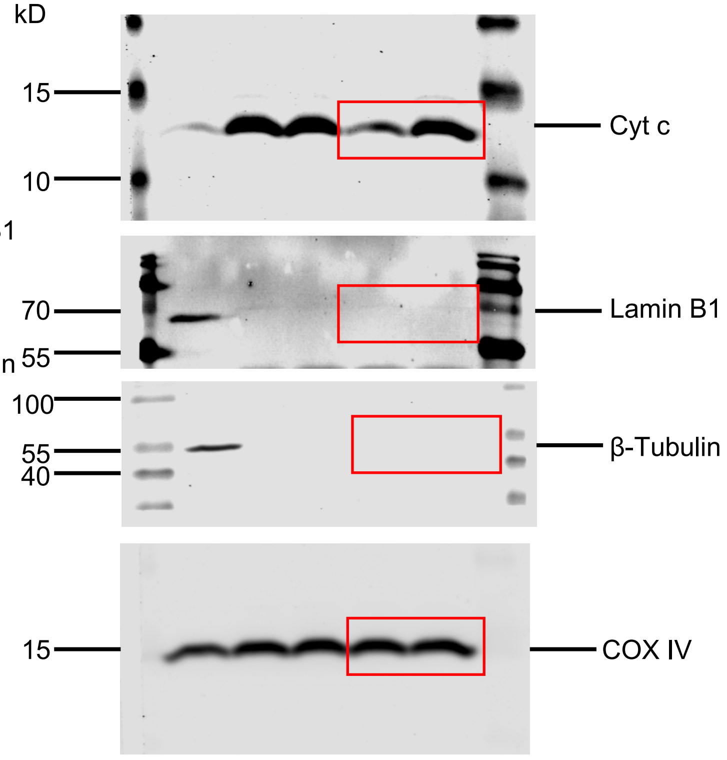

Full unedited blot for Fig. 2O

Right cytoplasm

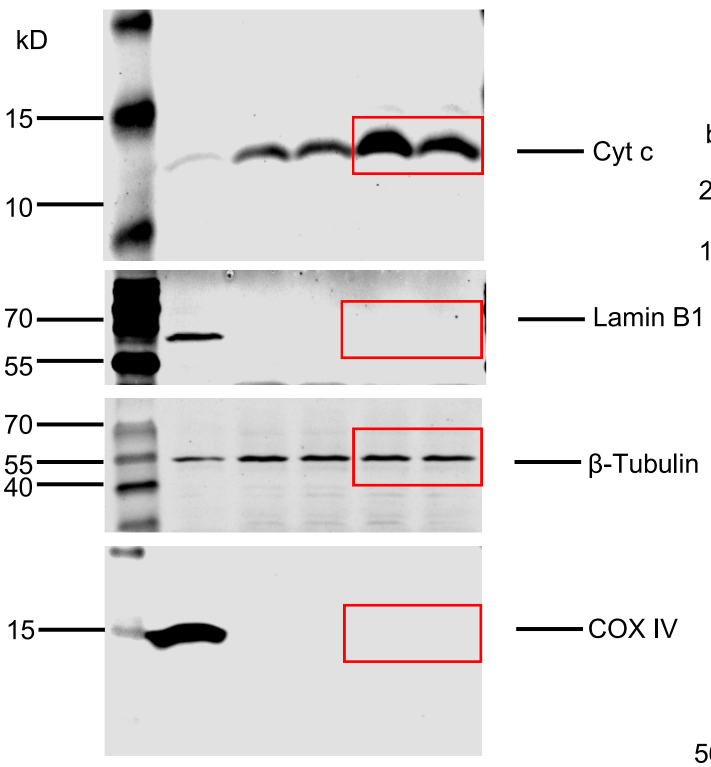

Full unedited blot and gel for Fig. 3J

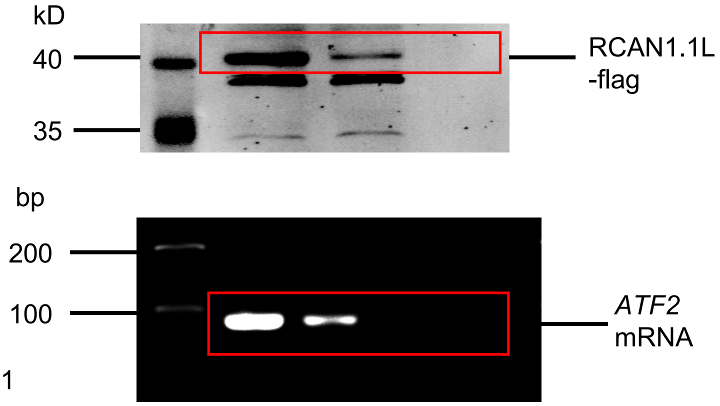

Full unedited blot for Fig. 3K

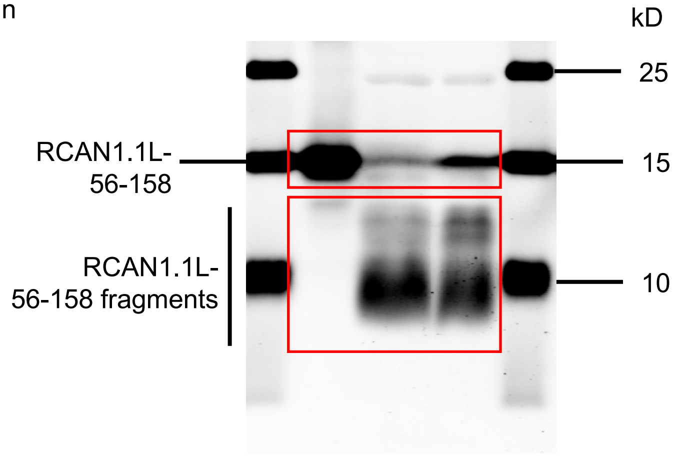

Full unedited gel for Fig. 4F

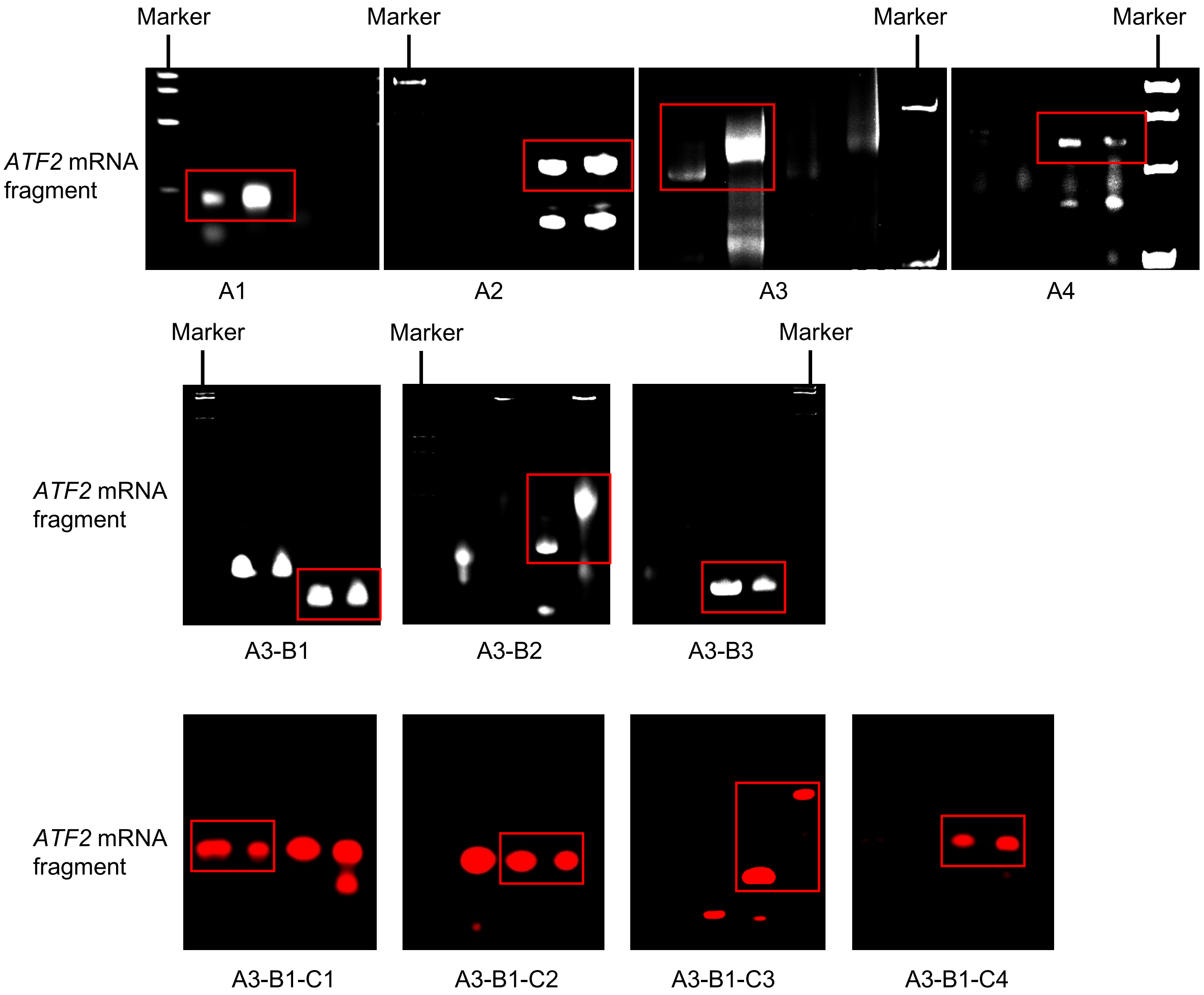

Full unedited blot for Fig. 4G

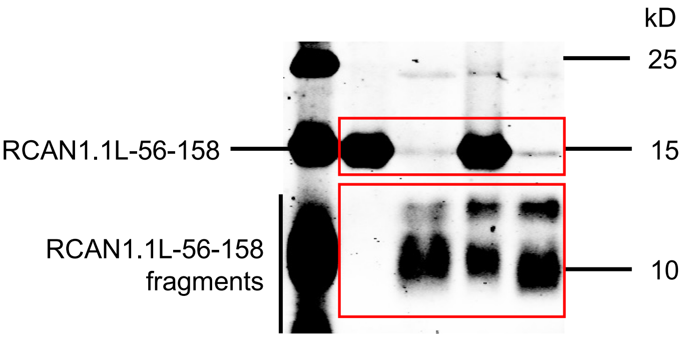

Full unedited blot for Fig. 4H

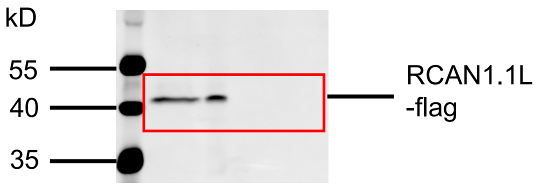

Full unedited blot for Fig. 4I

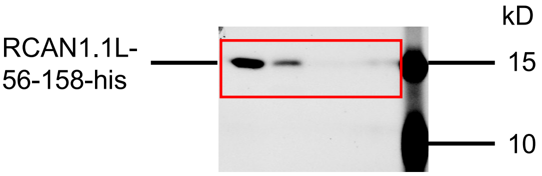

Full unedited blot for Fig. 5A

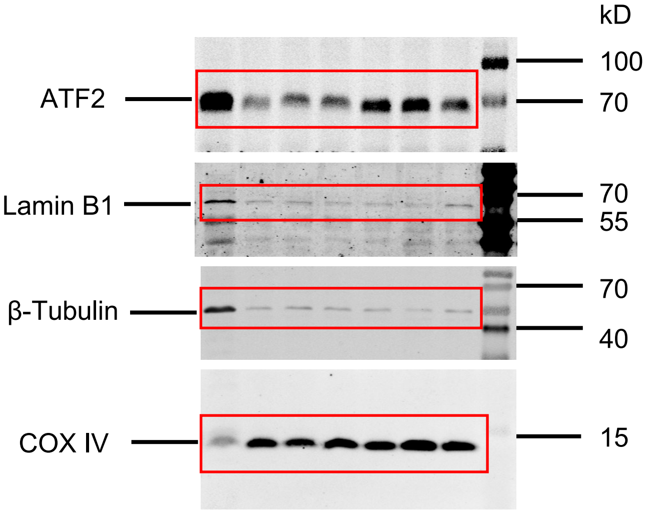

Full unedited blot for Fig.5C

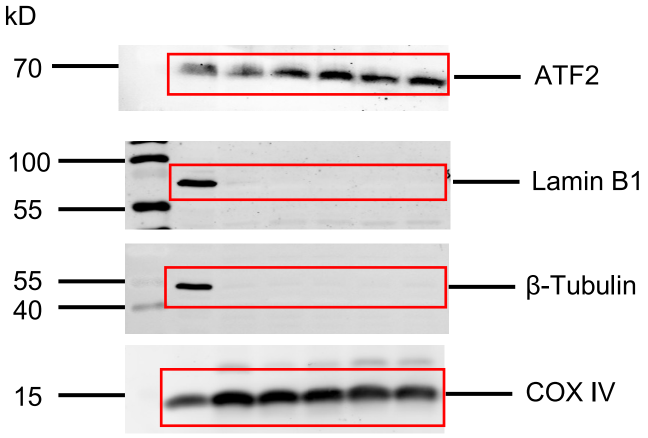

Full unedited blot for Fig. 5D

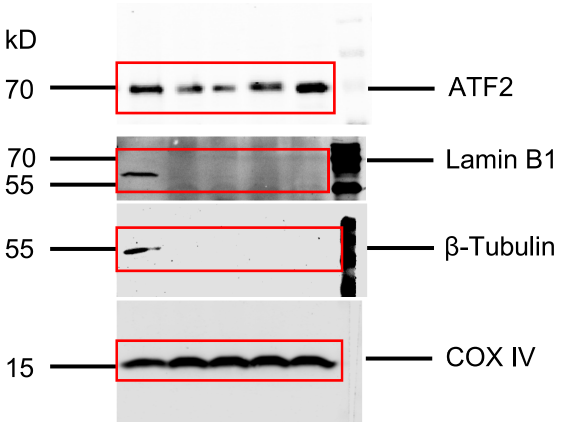

Full unedited blot for Fig. 5G

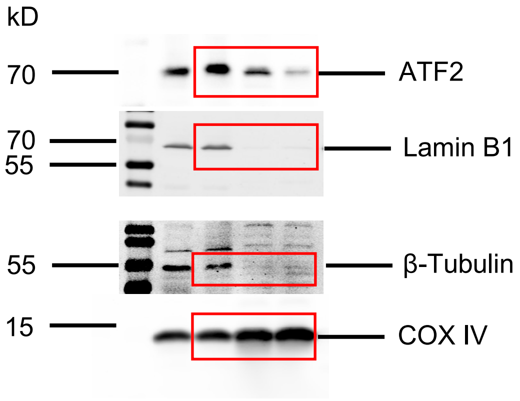

Full unedited blot for Fig. 6D

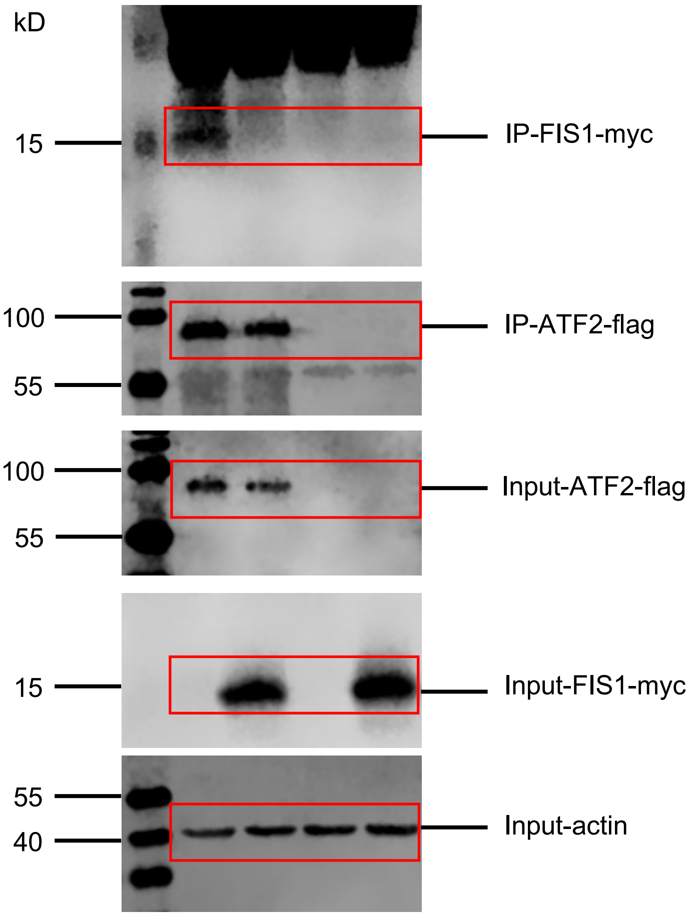

Full unedited blot for Fig. 6E

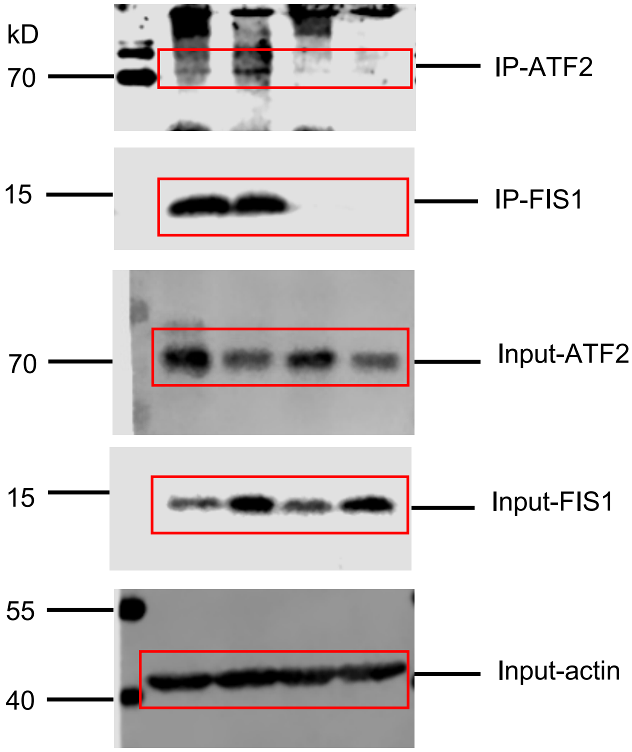

Full unedited blot for Fig. 6G

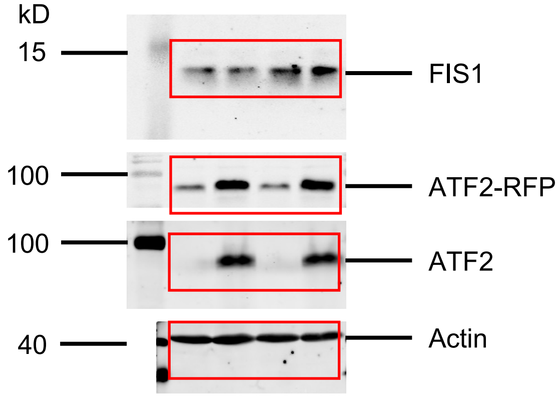

Full unedited blot for Fig. 6H

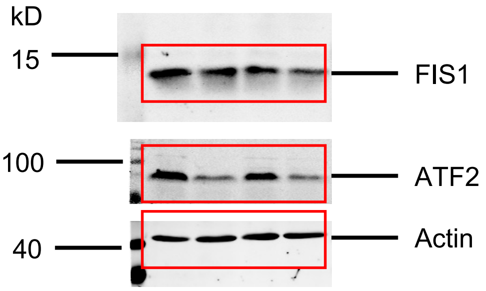

Full unedited blot for Fig. 6I

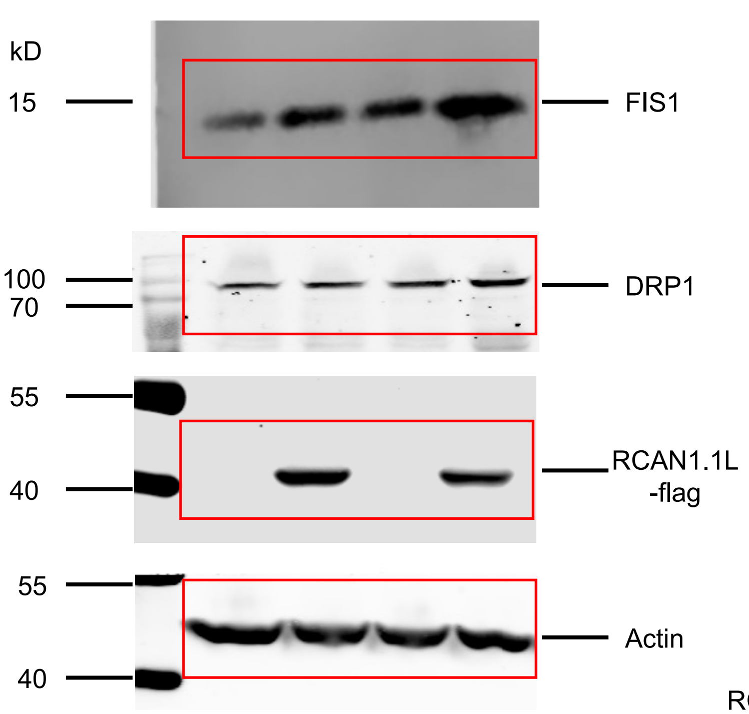

Full unedited blot for Fig. 6J

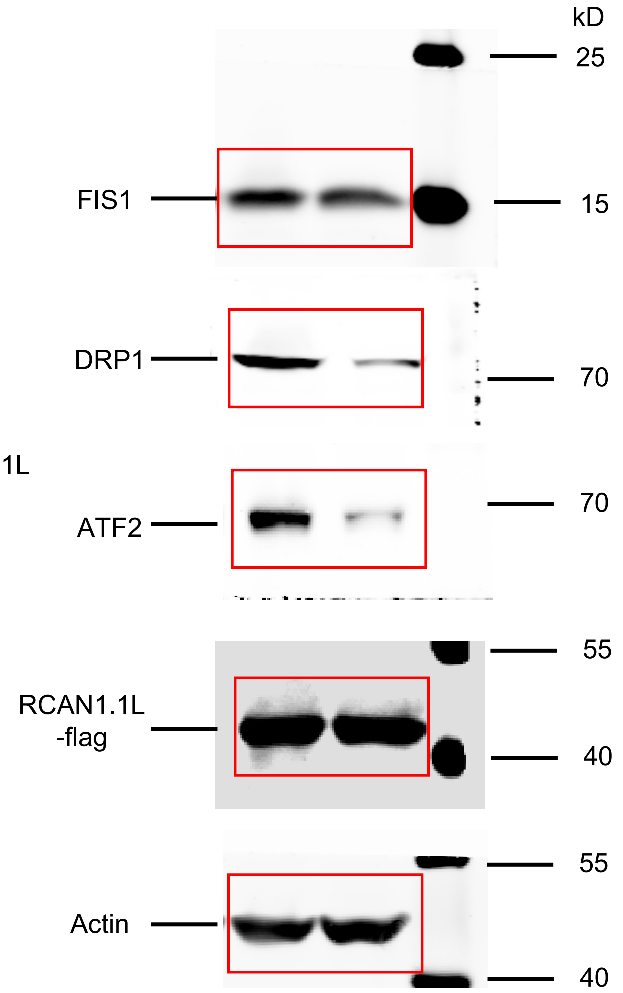

Full unedited blot for Fig. 6L

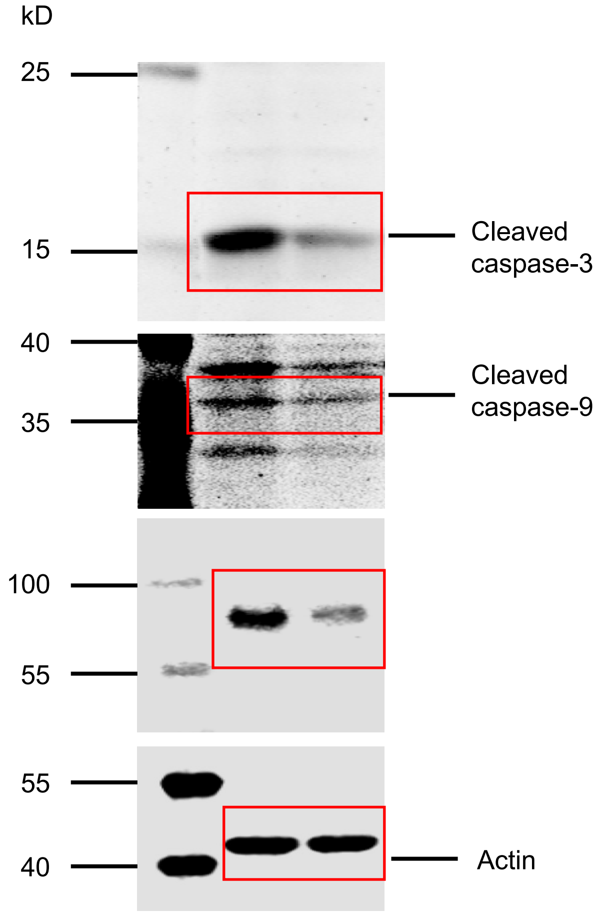

Full unedited blot for Fig. 7D

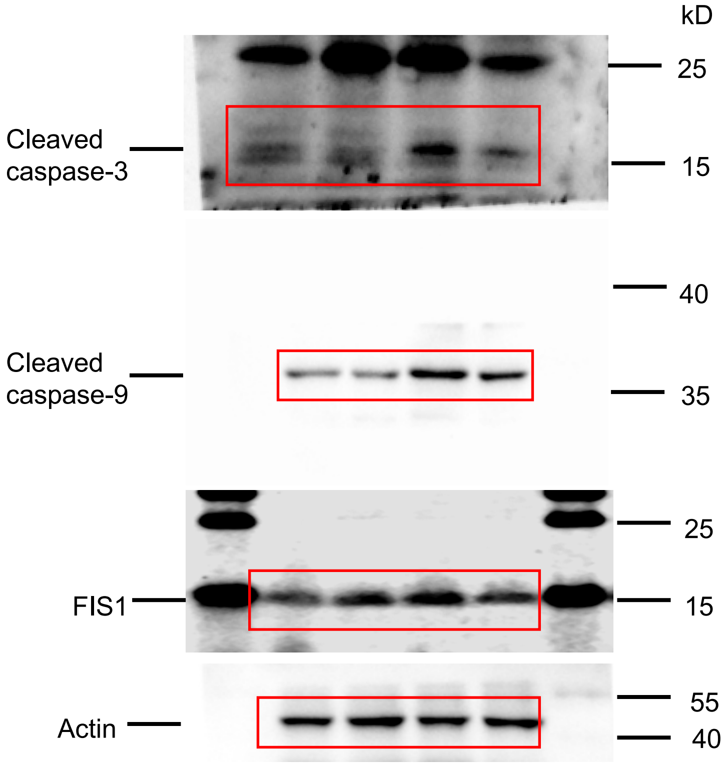

Full unedited blot for Fig. 7E

Mitochondria

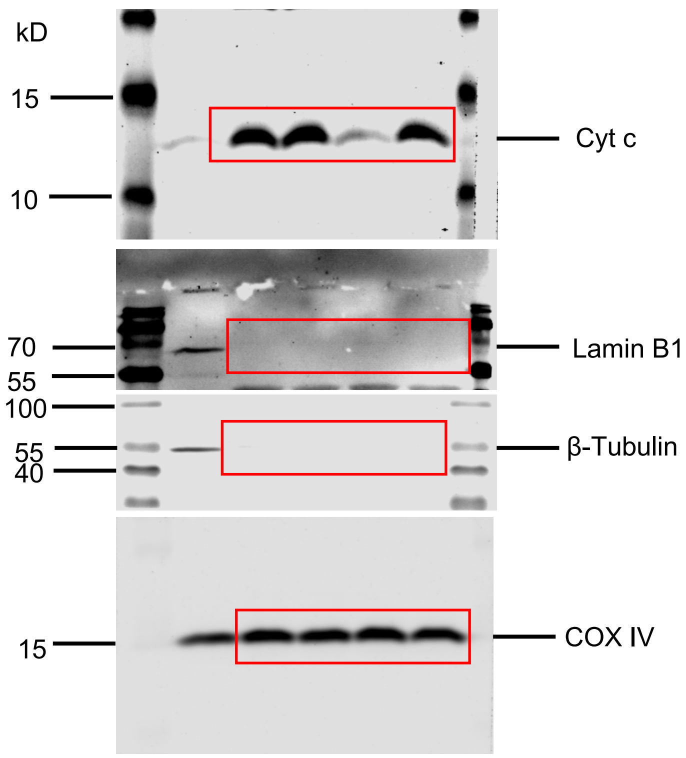

Full unedited blot for Fig. 7E

Cytoplasm

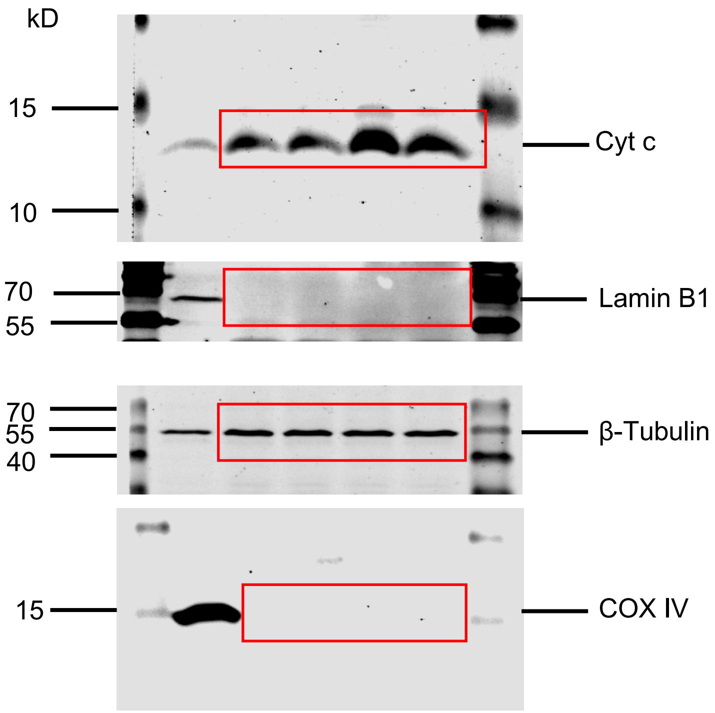

Full unedited blot for Fig. 7J

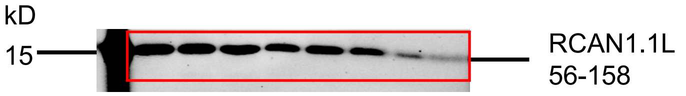

Full unedited gel for Fig. 7K

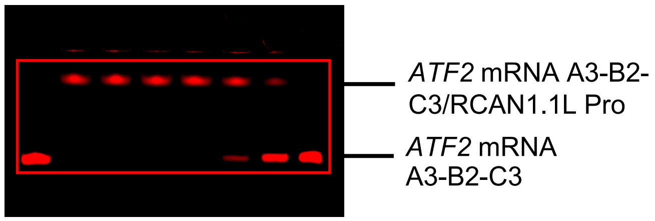

Full unedited blot for Fig. 7N

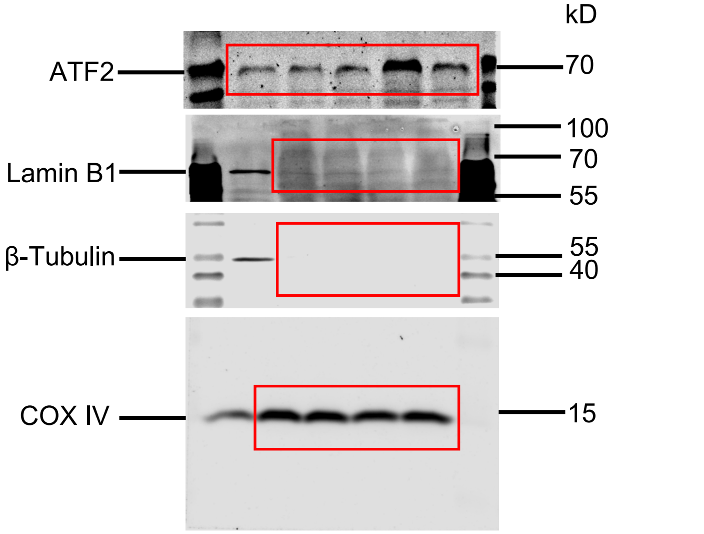

Full unedited gels for Fig. S1C

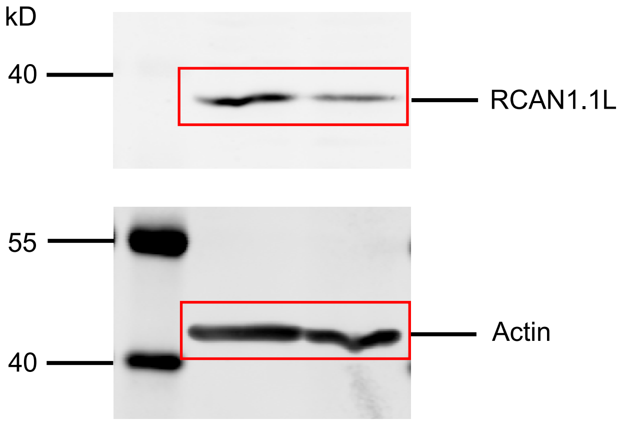

Full unedited gels for Fig. S2B

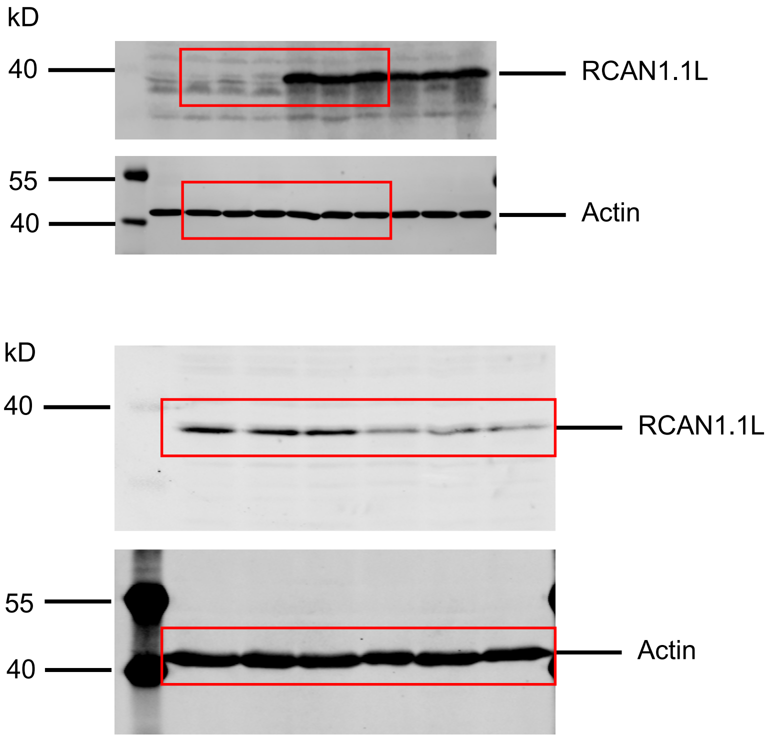

Full unedited gels for Fig. S2L

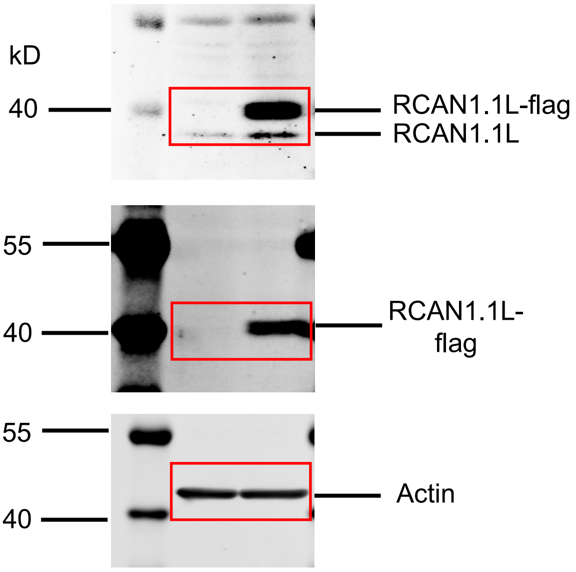

Full unedited gels for Fig. S2M

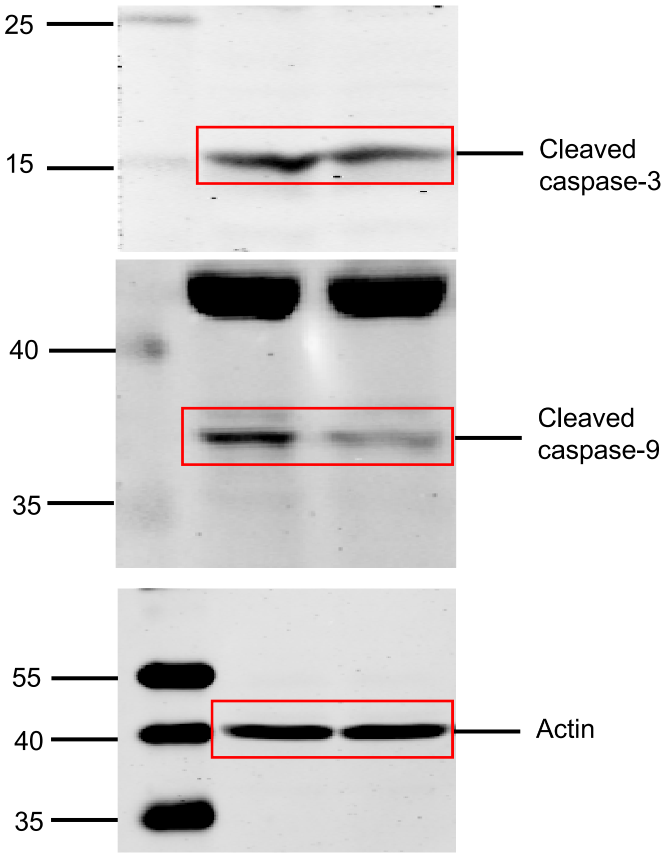

Full unedited gels for Fig. S3F

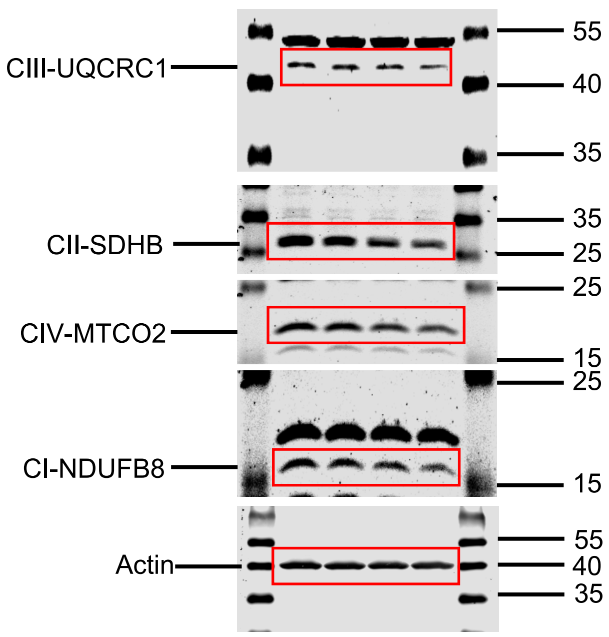

Full unedited gels for Fig. S3G

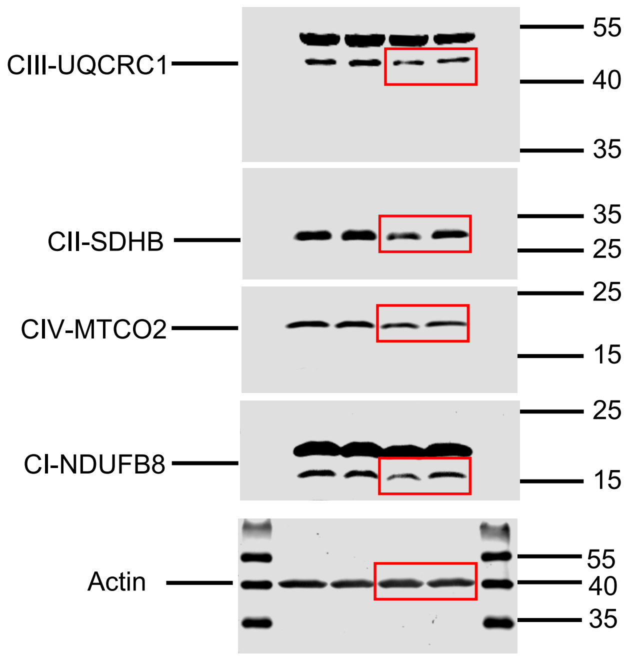

Full unedited gels for Fig. S4A

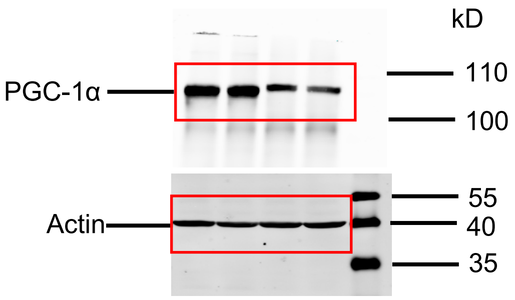

Full unedited gels for Fig. S4B

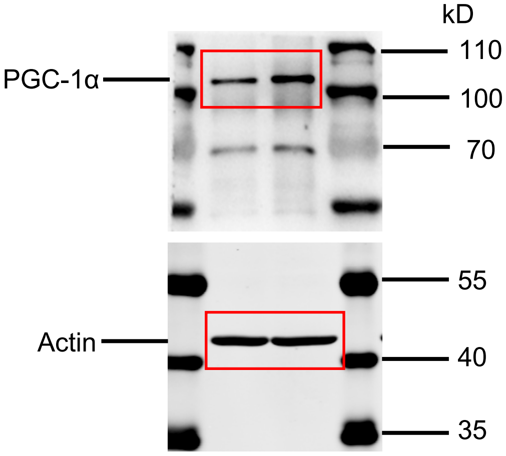

Full unedited gels for Fig. S6C

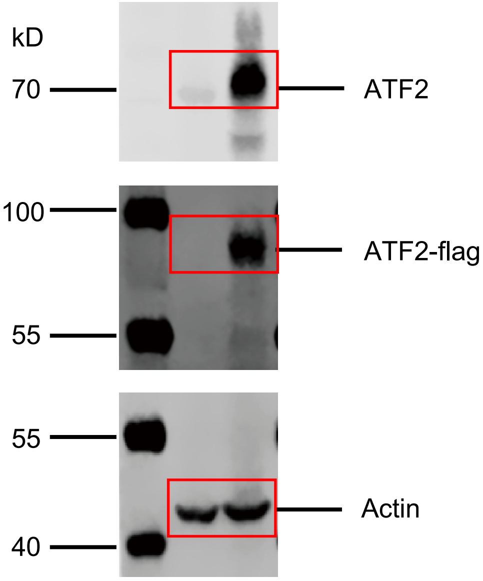

Full unedited gels for Fig. S6D

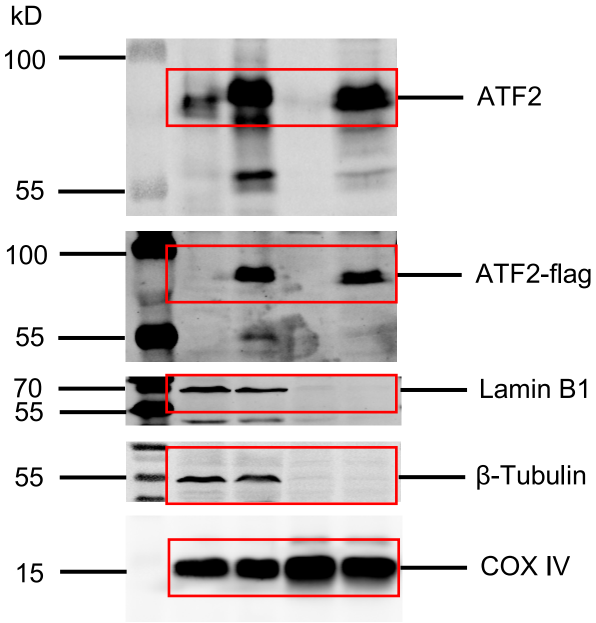

Full unedited gels for Fig. S6E

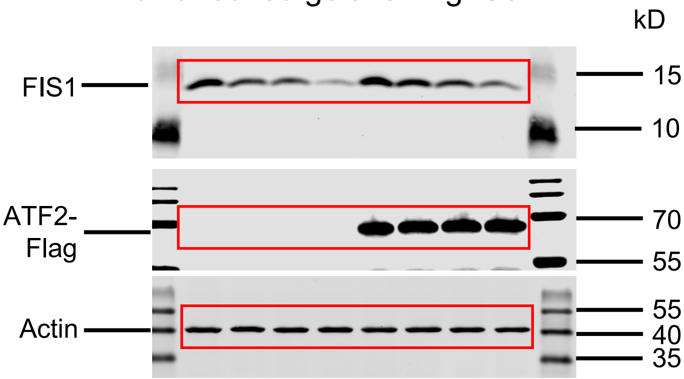

Full unedited gels for Fig. S6G

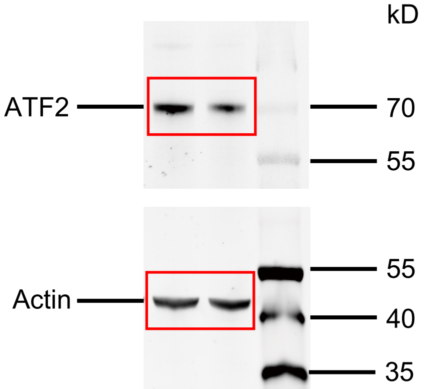

Full unedited gels for Fig. S6H

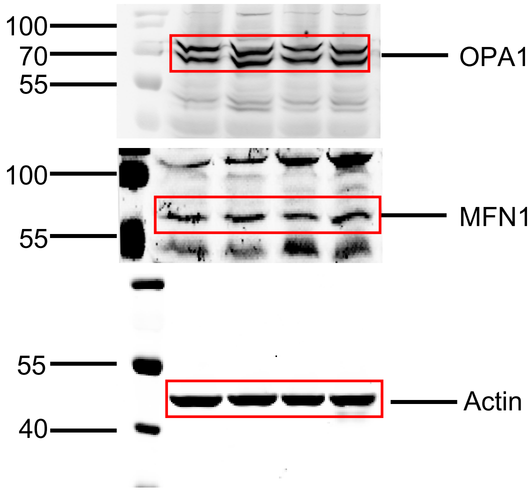

Full unedited gels for Fig. S6I

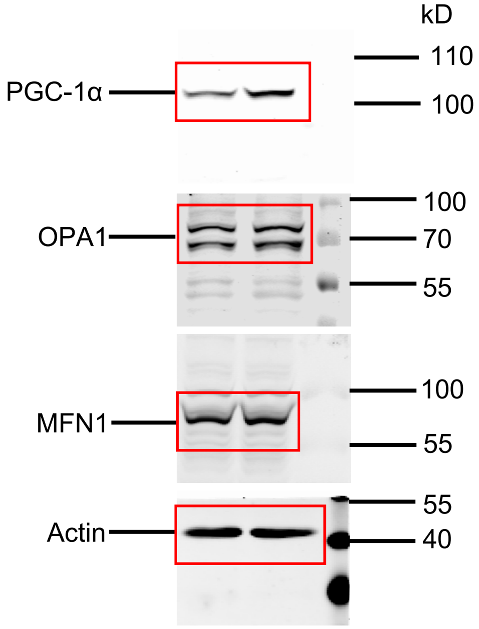

Full unedited gels for Fig. S7A

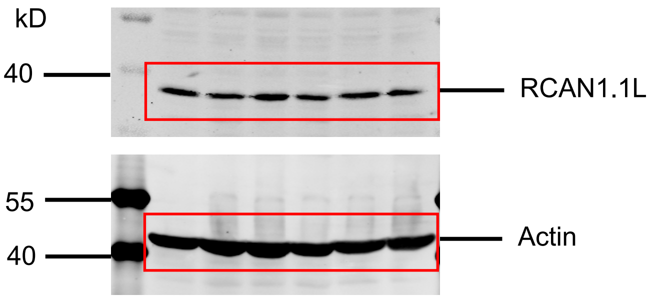

Full unedited gels for Fig. S7G

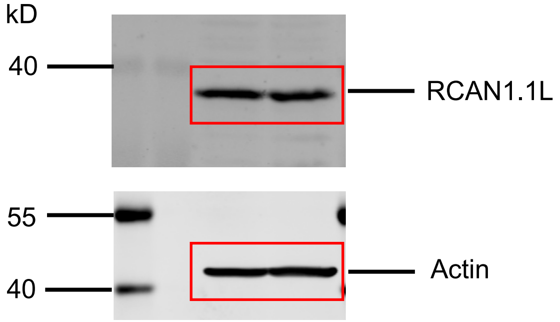

Full unedited gels for Fig. S7J

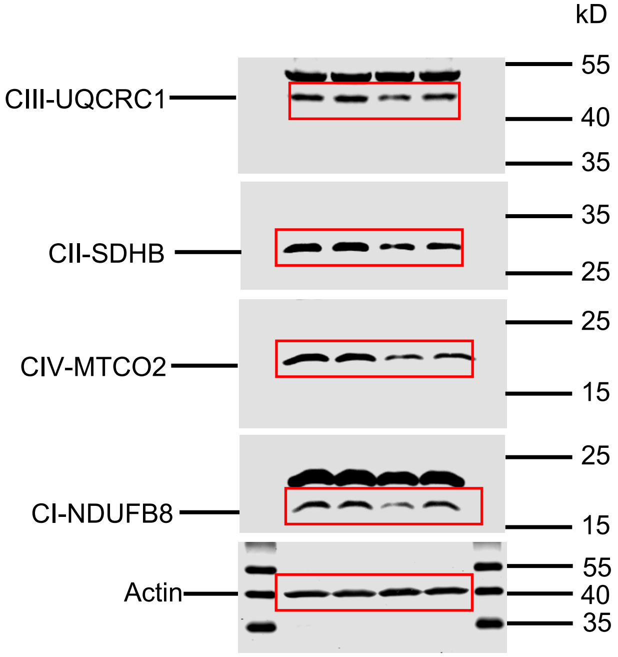

Supplement: Supplementary file 10 — Full and uncropped western blots [file 41419_2026_8809_MOESM10_ESM.pdf]
