# Supplementary material for: RNA-binding protein RCAN1.1L modulates ATF2 mRNA stability to promote mitochondrial fission in acute ischemic stroke
Source: Cell Death Dis. 2026 May 13;17(1):621. doi: 10.1038/s41419-026-08809-8 (PMC13342642; doi:10.1038/s41419-026-08809-8)
Supplement: Supplementary file 3 — Figure S2 [file 41419_2026_8809_MOESM3_ESM.pdf]

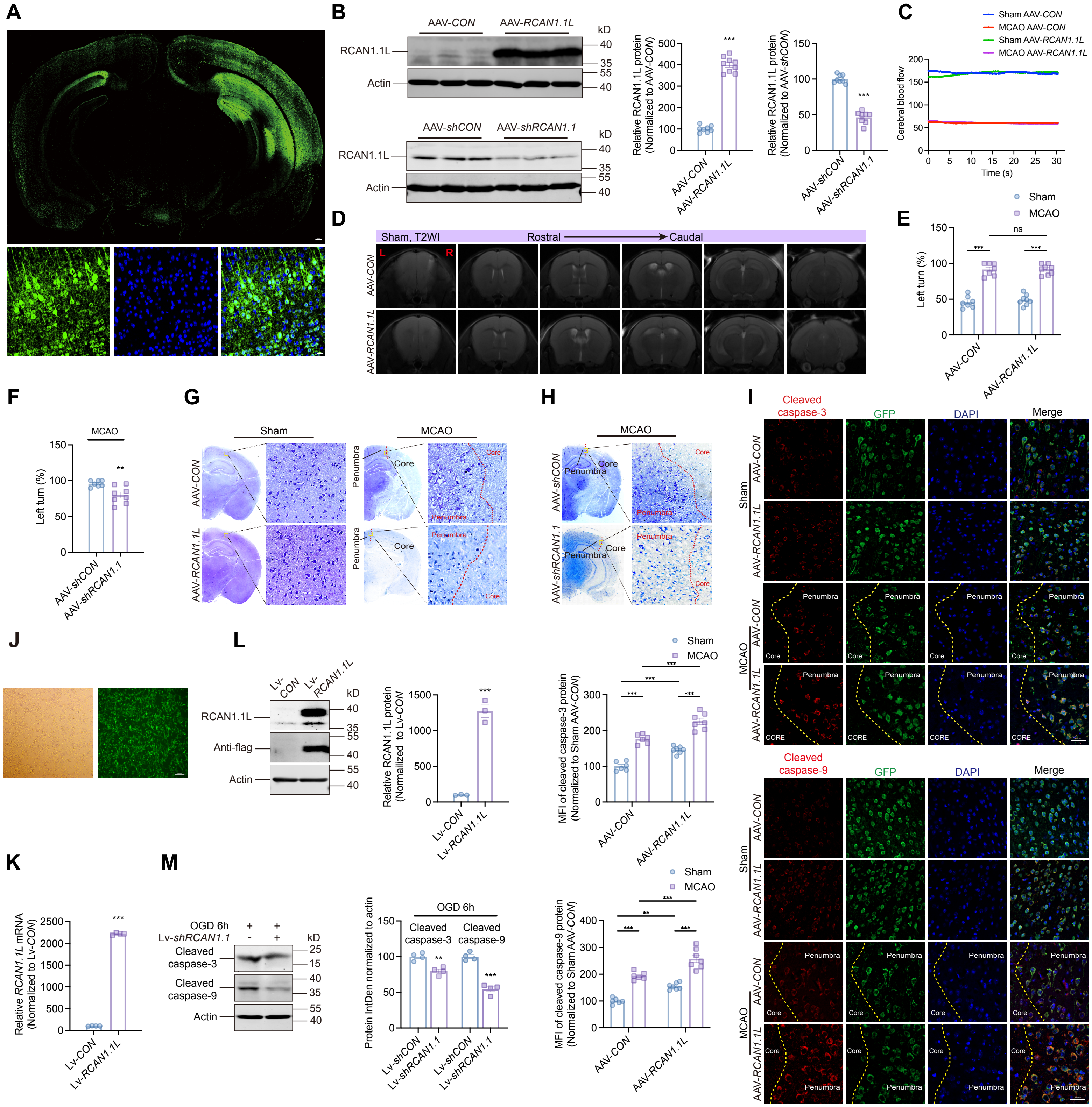
A
B
AAV-CON
AAV-RCAN1.1L
RCAN1.1L
Actin
AAV-shCON
AAV-shRCAN1.1
kD
Relative RCAN1.1L protein (Normalized to AAV-CON)
Relative RCAN1.1L protein (Normalized to AAV-shCON)
C
Sham AAV-CON
MCAO AAV-CON
Sham AAV-RCAN1.1L
MCAO AAV-RCAN1.1L
Cerebral blood flow
Time (s)
D
Sham, T2WI
Rostral
Caudal
E
Sham
MCAO
Left turn (%)
F
MCAO
G
Sham
MCAO
Penumbra
Core
H
I
Cleaved caspase-3
Cleaved caspase-9
GFP
DAPI
Merge
J
K
Relative RCAN1.1L mRNA (Normalized to Lv-CON)
Lv-CON
Lv-RCAN1.1L
L
Anti-flag
Relative RCAN1.1L protein (Normalized to Lv-CON)
MFI of cleaved caspase-3 protein (Normalized to Sham AAV-CON)
MFI of cleaved caspase-9 protein (Normalized to Sham AAV-CON)
M
OGD 6h
Lv-shRCAN1.1
Protein IntDen normalized to actin
Lv-shCON
