# Supplementary material for: RNA-binding protein RCAN1.1L modulates ATF2 mRNA stability to promote mitochondrial fission in acute ischemic stroke
Source: Cell Death Dis. 2026 May 13;17(1):621. doi: 10.1038/s41419-026-08809-8 (PMC13342642; doi:10.1038/s41419-026-08809-8)
Supplement: Supplementary file 8 — Figure S7 [file 41419_2026_8809_MOESM8_ESM.pdf]

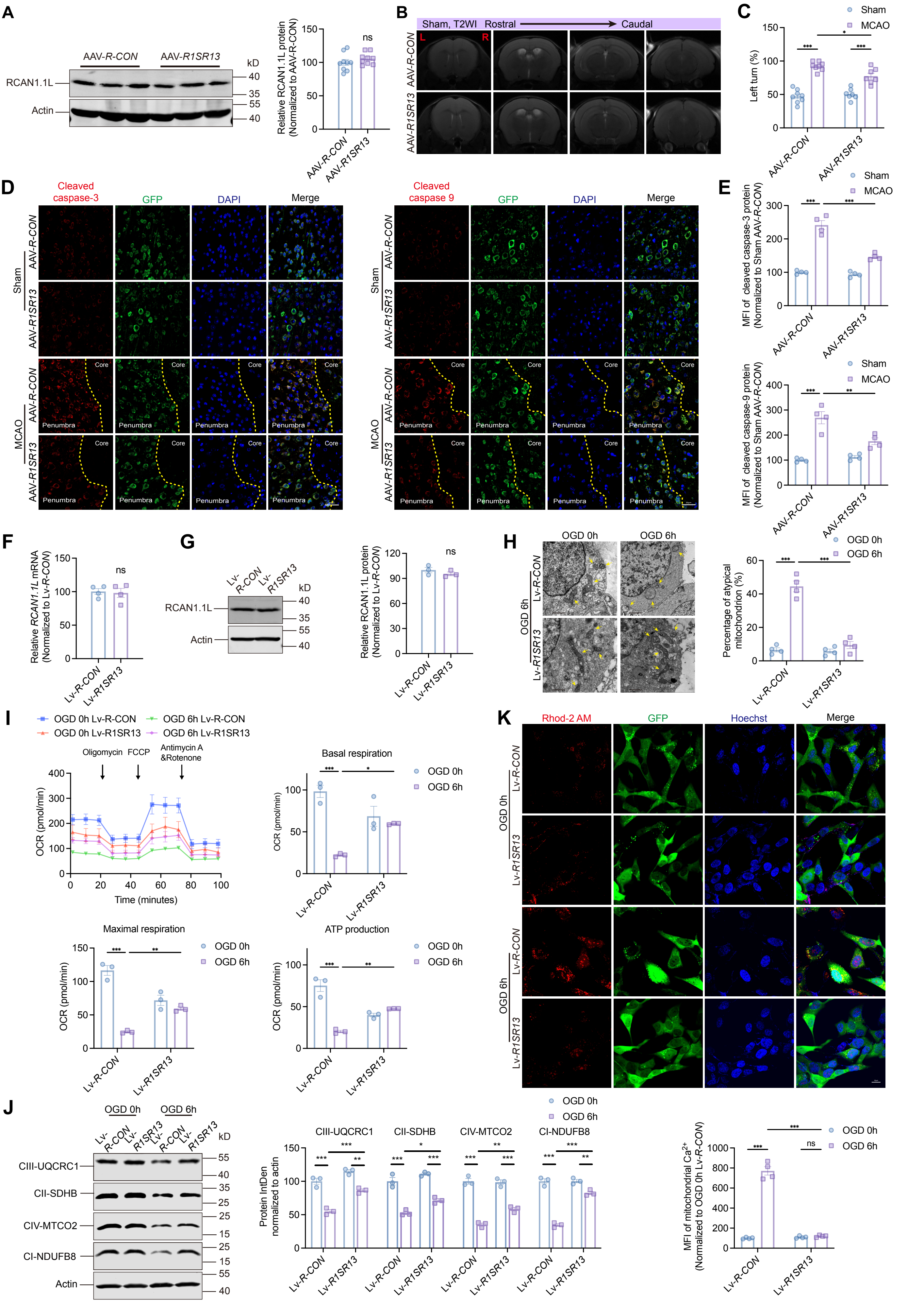

A
AAV-R-CON
AAV-R1SR13
kD
RCAN1.1L
Actin
Relative RCAN1.1L protein (Normalized to AAV-R-CON)
ns
B
Sham, T2WI Rostral
Caudal
L
R
C
Sham
MCAO
Left turn (%)
D
Cleaved caspase-3
GFP
DAPI
Merge
Cleaved caspase 9
Sham
MCAO
Core
Penumbra
E
MFI of cleaved caspase-3 protein (Normalized to Sham AAV-R-CON)
MFI of cleaved caspase-9 protein (Normalized to Sham AAV-R-CON)
F
Relative RCAN1.1L mRNA (Normalized to Lv-R-CON)
Lv-R-CON
Lv-R1SR13
G
Relative RCAN1.1L protein (Normalized to Lv-R-CON)
H
OGD 0h
OGD 6h
Percentage of atypical mitochondrion (%)
I
OGD 0h Lv-R-CON
OGD 0h Lv-R1SR13
OGD 6h Lv-R-CON
OGD 6h Lv-R1SR13
Oligomycin
FCCP
Antimycin A &Rotenone
OCR (pmol/min)
Time (minutes)
Basal respiration
Maximal respiration
ATP production
J
CIII-UQCRC1
CII-SDHB
CIV-MTCO2
CI-NDUFB8
Protein IntDen normalized to actin
K
Rhod-2 AM
GFP
Hoechst
Merge
MFI of mitochondrial Ca^{2+} (Normalized to OGD 0h Lv-R-CON)
